# Insight into phase-sensitive optical coherence tomography displacement measurement

**DOI:** 10.1038/s41467-026-77549-2

**Published:** 2026-09-16

**Authors:** Andrea Mazzolani, Jiayue Li, Balázs Dura-Kovács, Brendan F. Kennedy, Peter R. T. Munro

**Affiliations:** 1 Department of Medical Physics and Biomedical Engineering, University College London, London, UK; 2 BRITElab, Harry Perkins Institute of Medical Research, QEII Medical Centre Nedlands and Centre for Medical Research, The University of Western Australia, Crawley, WA Australia; 3 Department of Electrical, Electronic & Computer Engineering, School of Engineering, The University of Western Australia, Perth, WA Australia; 4 Institute of Physics, Faculty of Physics, Astronomy and Informatics, Nicolaus Copernicus University in Toruń, Toruń, Poland; 5 Present Address: Max Planck Institute of Quantum Optics, Garching, Germany

**Keywords:** Biomedical engineering, Applied mathematics

## Abstract

Phase-sensitive optical coherence tomography (OCT) measures motion from phase differences between repeated acquisitions, supporting techniques such as Doppler OCT and optical coherence elastography (OCE). A recent numerical and experimental study showed that, even without noise, displacement estimation is less accurate in dark speckles. Here, we demonstrate analytically why displacement is not proportional to phase difference in speckled OCT images. We derive an expansion for compression OCE containing higher-order terms beyond the conventional linear term and show that neglecting them introduces errors that increase with depth and strain-discontinuity magnitude. We also identify two phenomena that perturb the phase linearity, one producing features resembling phase-wrapping events that may be mitigated by phase-unwrapping techniques. Finally, we introduce a displacement-estimation method that overcomes these limitations and achieves errors two orders of magnitude lower than conventional approaches. These phase-instability findings may reshape motion measurement beyond compression OCE, including dynamic OCT.

## Abstract (web summary)

The authors provide a fundamental explanation of why phase difference in speckled optical coherence tomography can misrepresent motion and introduce a more accurate method for estimating displacement and strain.

## Introduction

Optical coherence tomography (OCT) is increasingly being used to perform functional and label-free imaging. For example, Doppler OCT is an important extension to OCT as it allows the measurement of blood velocity and flow^1,2^. Optical coherence elastography (OCE)^3–8^ is another powerful label-free imaging technique that obtains three-dimensional maps of the mechanical properties of biological tissues. Doppler OCT and OCE images are formed from the acquisition of at least two optical coherence tomography (OCT)^9,10^ images of the sample, each acquired at different times. Dynamic OCT has emerged recently as a powerful label-free approach for observing cell physiology, most commonly by analyzing temporal fluctuations in the intensity or amplitude of the OCT signal^11^. Although conventional dynamic OCT metrics are generally not based on phase-difference displacement estimation, recent studies have begun to investigate phase-based dynamic OCT analyses. For example, Schulz-Hildebrandt et al.^12^ introduced a phase-sensitive dynamic OCT approach to quantify intracellular motion from phase changes between repeated complex OCT measurements, while Bellesini et al.^13^ included phase variance among the methods used to visualize three-dimensional dynamic OCT contrast for breast tumor margin assessment.

The implementation of these techniques leverages the sub-nanometer sensitivity to sample axial displacement^14^.

We show in this paper, however, why this high sensitivity is significantly compromised in noise-free OCT images containing speckle. For clarity, in the remainder of this paper we consider the application of quasi-static OCE, since considering both local strain and bulk displacement allows a range of other OCT modalities to be analyzed also. For example, to first approximation, Doppler OCT acquires one image, followed by another image after a time delay, during which the sample has undergone bulk displacement due to flow.

In phase-sensitive OCE^5,6^, the phase difference between OCT signals is calculated to estimate the axial displacement, *d*(*z*), of tissue at axial depth *z*, by using the following equation^3,5,15–17^, which we denote as the phase-difference equation (PHDE):

1 $$d(z)\approx \frac{\Delta \phi (z)}{2\eta {k}_{0}},$$

where *k*_0_ is the central wavenumber, *η* is the background refractive index and Δ*ϕ*(*z*) ≔ *ϕ*^*U*^(*z*) − *ϕ*^*L*^(*z*) is the difference in phase of unloaded and loaded A-scans and we use the superscripts *U*, *L* to denote the unloaded and loaded signals, respectively. In order to simplify the calculations, for the remainder of this article we assume without loss of generality that *η* = 1.

Although strain is dimensionless, for clarity throughout the manuscript we use the symbol *ϵ* as a strain marker, while $\hat{\epsilon }$ denotes the strain value used in the model; the hat is used only to distinguish the strain variable from the dimensionless unit *ϵ*. For example, $\hat{\epsilon }=-3\,m\epsilon$ corresponds to a compressive strain of −0.3%, where *m* denotes the metric prefix milli. Under the assumption of constant local axial strain, the axial displacement within the sample can be written as $d(z):=D+\hat{\epsilon }z$, where *D* is called bulk displacement, with the same units as *z*, and $\hat{\epsilon }$ is the local axial strain at depth *z*. The bulk displacement occurs as a result of axial strain discontinuities across tissue.

In this local description, *D* accounts for the difference between the actual local displacement at depth *z* and the displacement that would be obtained in a hypothetical loaded configuration where the same strain $\hat{\epsilon }$ is applied uniformly throughout the entire sample. Therefore, *D* > 0 means that the actual loaded position is shifted towards larger *z* compared with this hypothetical uniformly strained loaded configuration, whereas *D* < 0 means that it is shifted towards smaller *z* (see Supplementary Note 3 for further details).

Conventional models estimate $\hat{\epsilon }$ from experimental data by performing a linear fit to Δ*ϕ*(*z*), using methods such as weighted least squares (WLS)^5,14,18^, or by applying finite difference methods to complex-valued optical coherence tomography signals to obtain $\Delta {\phi }^{{\prime} }(z)$, such as the vector method (VM)^6,19,20^. Recently, a deep learning-based model was demonstrated to reconstruct the unwrapped Δ*ϕ* from its wrapped version^21,22^, in order to improve fitting-based strain retrieval. In the literature, equation (1) is frequently assumed to be a mathematical identity, meaning that differences between *d*(*z*), calculated using equation (1), and the actual sample displacement are usually attributed to noise or artifacts of the unwrapping algorithm^3,23^.

Makita et al.^24^ conducted a statistical analysis of Δ*ϕ* under the assumption of random scatterer displacement, demonstrating how it varies with noise. In this context, equation (1) was described as an approximation, and a quantitative error analysis was performed. In a deterministic displacement of scatterers approach, which is the assumption we make in this article, Hepburn et al.^25^ explained that the assumption that equation (1) is a mathematical identity is valid in some cases, such as for specular reflections. The same paper, however, also shows that in low-intensity regions known as dark speckles, where the intensity of the OCT signal is low, estimations of displacement based on the PHDE are less accurate than in regions of high OCT signal intensity, called bright speckles. However, a comprehensive and complete explanation of this inaccuracy has, to the best of our knowledge, not yet been provided. Furthermore, previous works have not proposed a method to recover accurate displacement or strain. An understanding of this is important as it reveals why conventional models of strain retrieval can be inaccurate when applied in turbid tissue, which generally results in speckled OCT images. In contrast, our study introduces a displacement-estimation method specifically designed to address this limitation, distinguishing it from prior approaches.

In this article, we derive an equation that shows why Δ*ϕ*(*z*) is, in general, a non-linear function of *z* in regions of constant strain, in contradiction with the prevailing understanding. We do this by using a one-dimensional (1D) model of OCT image formation ^9^ to derive an equation connecting OCT signals under different amounts of constant strain. We then use this equation to derive an analytic approximation for Δ*ϕ* which includes terms additional to equation (1). We then use this analytical approximation to show that Δ*ϕ* exhibits intrinsic oscillations in both dark and bright speckles.

An important result of this paper is an explanation of two perturbations of Δ*ϕ*, which occur in the vicinity of dark speckles, that we will call *oscillation peaks* and 2*π*-jumps. Both of these perturbations reduce the accuracy of conventional OCE models based on the PHDE. We will also show that 2*π*-jumps are often misinterpreted as phase-wrapping artifacts, which misdirect research towards improving phase-unwrapping algorithms, rather than developing models that account for these 2*π*-jumps. We then analyze, qualitatively and quantitatively, the accuracy of conventional strain-retrieval methods employed in OCE. In particular, we show that the true relationship between *d*(*z*) and Δ*ϕ* includes non-linear terms neglected by equation (1). Furthermore, we show that these non-linear terms are non-negligible in the case of large sample displacements (commonly occurring in deep regions of the sample) or large strain discontinuities across the sample. In these cases, the accuracy of conventional models in OCE is significantly reduced. Finally, we introduce a displacement-estimation method for OCE that addresses the limitations of existing approaches and improves the accuracy of strain retrieval in OCE. This method achieves an error two orders of magnitude lower than traditional techniques and demonstrates a markedly higher strain SNR, making it a robust solution for overcoming the challenges posed by noise and inaccuracies in conventional OCE methods.

## Results

### Mapping between loaded and unloaded A-scans

By assuming a 1D model of OCT image formation, a Gaussian power spectral density, and by neglecting the auto-correlation terms and the mirror image, an OCT A-scan can be written as ^9^:

2 $$A(z):=\sum_{j=1}^{N}{\rho }_{j}{e}^{i2{k}_{0}\left(z-{z}_{j}\right)}{e}^{-{W}^{2}{\left(z-{z}_{j}\right)}^{2}}$$

where *z*_*j*_ and *ρ*_*j*_ are the axial coordinate and parameter representing the scattering cross-section of the *j*th scatterer, respectively. The choice of the parameter *W* controls the width of the Gaussian terms in Eq. (2), and *W* has inverse units of *z*, typically m^−1^. Specifically, let *ξ* denote the spatial-frequency variable Fourier-conjugate to *z*, and let ${\xi }_{0}=\frac{{k}_{0}}{2\pi }$ be the central spatial frequency. In Eq. (2) and in the rest of this article we assume a Gaussian power spectral density ${S}_{G}(\xi ):=\frac{1}{\sqrt{\pi }W}{e}^{-{\left(\frac{2\pi (\xi -{\xi }_{0})}{W}\right)}^{2}}$. In practice, starting from a finite full spectral interval *B* in spatial-frequency space, *W* is chosen by prescribing the relative value *τ* of *S*_*G*_(*ξ*) at the interval edges, yielding $W=\frac{\pi B}{\sqrt{\log (1/\tau )}}$. In the simulations, we use *τ *≤ 10^−2^, so that the Gaussian power spectral density is negligible at the edges of the spectral interval. We also verified that the method works for larger values of *τ*, for which *S*_*G*_(*ξ*) has a non-negligible value at the interval edges. For general experimental data, this Gaussian-spectrum assumption can be satisfied in practice by reshaping the measured power spectral density to a Gaussian profile before applying the algorithm; further details are provided in Supplementary Notes 7.1 and 7.2.

Under the assumption of piecewise constant strain, upon loading, each scatterer coordinate is mapped to ${z}_{j}^{L}:=D+\left(1+\hat{\epsilon }\right){z}_{j}^{U}$. The unloaded and loaded A-scans are then given as:

3a $${A}_{scan}^{U}(z):=\sum_{j=1}^{N}{\rho }_{j}{e}^{i2{k}_{0}(z-{z}_{j}^{U})}{e}^{-{W}^{2}{(z-{z}_{j}^{U})}^{2}}$$

3b $${A}_{scan}^{L}(z):=\sum_{j=1}^{N}{\rho }_{j}{e}^{i2{k}_{0}(z-{z}_{j}^{L})}{e}^{-{W}^{2}{(z-{z}_{j}^{L})}^{2}}$$

3c $$\qquad=\sum_{j=1}^{N}{\rho }_{j}{e}^{i2{k}_{0}(z-(D+(1+\hat{\epsilon }){z}_{j}^{U}))}{e}^{-{W}^{2}{(z-(D+(1+\hat{\epsilon }){z}_{j}^{U}))}^{2}}$$

To simplify the calculations and notation, it is convenient to factor out the main phase term ${e}^{i2{k}_{0}z}$ from each A-scan, by defining:

4a $${A}^{U}(z):={e}^{-i2{k}_{0}z}{A}_{scan}^{U}(z)=\sum_{j=1}^{N}{\rho }_{j}{e}^{-i2{k}_{0}{z}_{j}^{U}}{e}^{-{W}^{2}{\left(z-{z}_{j}^{U}\right)}^{2}}$$

4b $${A}^{L}(z):={e}^{-i2{k}_{0}z}{A}_{scan}^{L}(z)=\sum_{j=1}^{N}{\rho }_{j}{e}^{-i2{k}_{0}{z}_{j}^{L}}{e}^{-{W}^{2}{\left(z-{z}_{j}^{L}\right)}^{2}}$$

In particular, when applying equations (4) to OCT signals computed with equations (3), the phases are demodulated by eliminating the linear phase term 2*k*_0_*z*, which is common in both unloaded and loaded OCT signals. Consequently, the OCT phase difference Δ*ϕ*, which is the focus of this paper, remains unaffected.

**Fig. 1:**
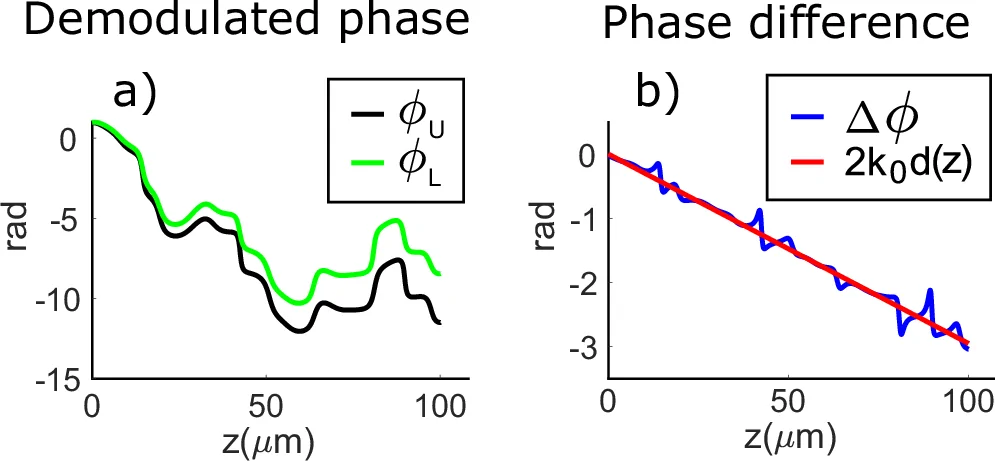
OCT Phase-difference oscillations. **a** Phases of the unloaded (*ϕ*^*U*^, black) and loaded (*ϕ*^*L*^, green) OCT A-scans. Each A-scan was pre-multiplied by ${e}^{-2i{k}_{0}z}$ (see equation (4)). **b** Phase difference Δ*ϕ*(*z*) = *ϕ*^*U*^(*z*) − *ϕ*^*L*^(*z*) (blue) between the two OCT A-scans, containing oscillations that make strain retrieval difficult. The red line shows the linear prediction 2*k*_0_*d*(*z*), where *k*_0_ is the central wavenumber and *d*(*z*) is the axial displacement, as defined in equation (1).

Figure 1 shows an example of noise-free simulated unloaded and loaded OCT signals, where *D* = 0 and $\hat{\epsilon }=-3m\epsilon$, calculated with equation (4). In plot a, the phases of the unloaded and loaded OCT signals are plotted in black and green, respectively. Plot b shows how Δ*ϕ*(*z*) (blue line) possesses intrinsic oscillations, causing it to deviate from the expected linear function, given the known constant strain (red line).

Now we derive a series expansion that allows us to express the loaded A-scan as a function of only terms related to the unloaded A-scan. We start by defining a generalization, ${A}_{n}^{U}(z)$, of *A*^*U*^(*z*), as:

5 $${A}_{n}^{U}(z):=\sum_{j=1}^{N}{\rho }_{j}{\left(z-{z}_{j}^{U}\right)}^{n}{e}^{-i2{k}_{0}{z}_{j}^{U}}{e}^{-{W}^{2}{\left(z-{z}_{j}^{U}\right)}^{2}},\quad \forall {{\rm{n}}}\in {\mathbb{N}},$$

It is easy to verify that, in the case of *n* = 0, equation (5) reproduces the unloaded A-scan, namely, ${A}_{0}^{U}(z)\equiv {A}^{U}(z)$. After making some algebraic manipulations and using the Taylor expansion, we express *A*^*L*^(*z*) as a series of the terms ${A}_{n}^{U}(z)$ (see Supplementary Note 1 for further details):

6 $${A}^{L}(z):=\sum_{n=0}^{\infty }{\alpha }_{n}(z){A}_{n}^{U}(z),$$

where the terms ${\alpha }_{n}$ are a function of $z,W,\hat{\epsilon }$ and *D*, but do not depend on the scatterer locations *z*_*j*_.

The explicit expressions of the first two terms are ${\alpha }_{0}={e}^{i\psi -{W}^{2}{d}_{0}}$ and ${\alpha }_{1}={e}^{i\psi -{W}^{2}{d}_{0}}\left[i\,2{k}_{0}\hat{\epsilon }+2{W}^{2}(1+\hat{\epsilon })(\hat{\epsilon }z+D)\right]$, where $\psi=-2{k}_{0}(D+\hat{\epsilon }z)$ and ${d}_{0}={(\hat{\epsilon }z+D)}^{2}$. Higher-order coefficients are not reported explicitly here, but can be derived using the procedure described in Supplementary Note 1.1.

**Fig. 2:**
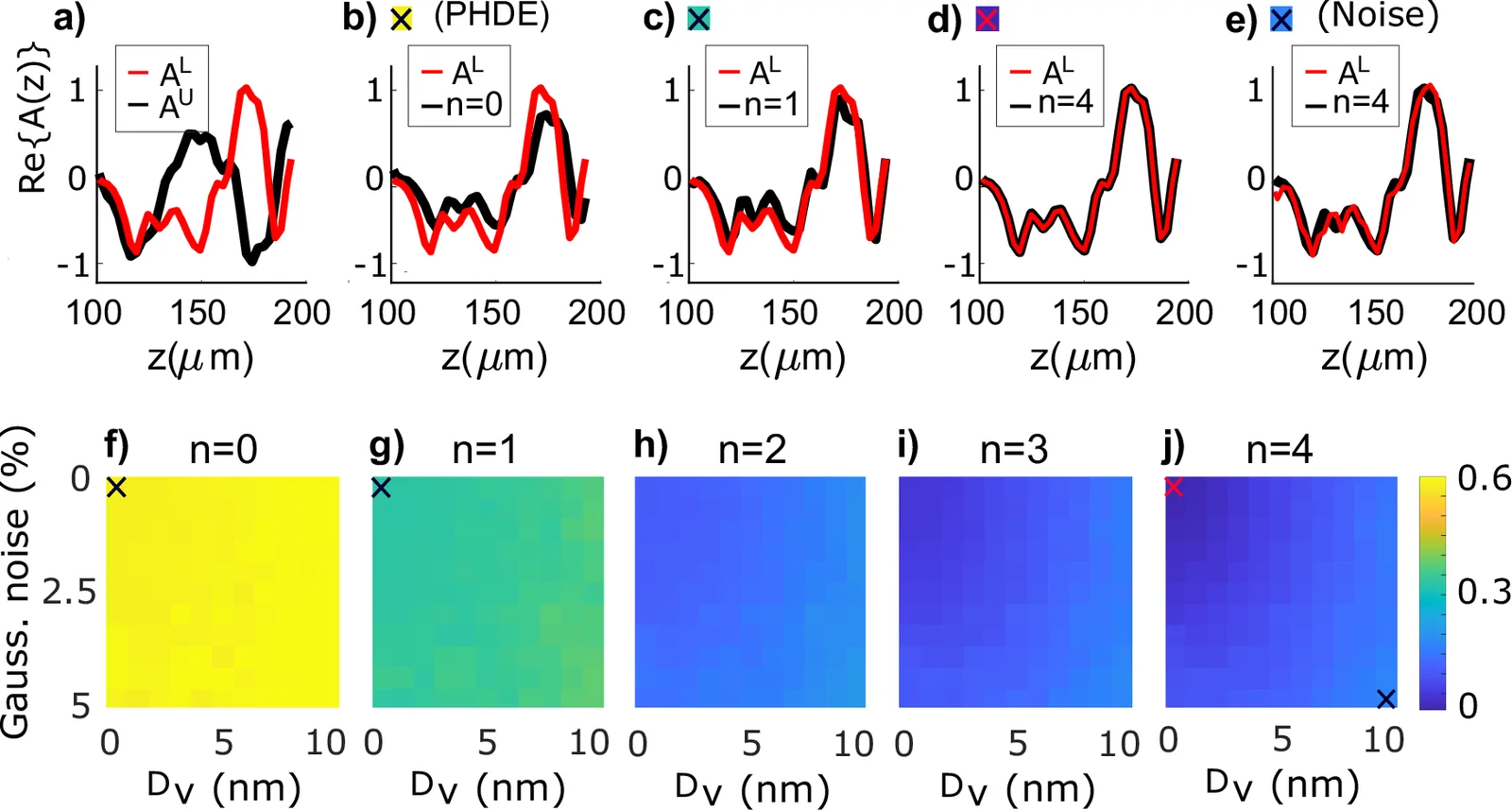
Convergence of the loaded A-scan expansion under noise and vibration. Accuracy of the truncated expansion in Eq. (6) for reconstructing the loaded A-scan, where *A*^*U*^ and *A*^*L*^ denote the unloaded and loaded A-scans, respectively. Each pixel in **f**–**j** shows the integral relative error ${{{\mathcal{E}}}}_{2}$ between *A*^*L*^ and the approximation obtained from Eq. (6) when the series is truncated at *n* = 0, 1, 2, 3, and 4, respectively. In these panels, additive complex Gaussian noise ranging from 0 to 5% was added to both the real and imaginary parts of the loaded A-scans, while an additional vibrational displacement ranging from 0 to 10 nm was added to the loaded scatterer positions. **a**–**e** show representative A-scan examples. (**a**) compares the real parts of *A*^*L*^ and *A*^*U*^, while (**b**), (**c**), and (**d**) compare *A*^*L*^ with the approximation obtained from Eq. (6) without noise, truncated at *n* = 0, 1, and 4, respectively. **e** shows the corresponding noisy and vibrational case for *n* = 4, where the maximum levels of additive complex Gaussian noise and vibrational displacement with standard deviation *D*_*V*_ were applied. The black crosses in (**f**) and (**g**) correspond to the examples shown in (**b**) and (**c**), respectively; the red cross in (**j**) corresponds to (**d**), and the black cross in (**j**) corresponds to (**e**).

Plots a–d of Fig. 2 show, respectively, the real part of the unloaded A-scan and the approximations of the loaded A-scan obtained by truncating the sum in Eq. (6) at *n* = 0, *n* = 1, and *n* = 4, each compared with the actual loaded A-scan. The accuracy of the estimation degrades as the magnitude of $\hat{\epsilon }$ and *D* increases, because additional terms of the truncated Taylor series must be calculated in these cases. However, when performing OCE, we can typically assume $| \hat{\epsilon }| \le 10m\epsilon$, ∣*D*∣ ≤ 10 μm, where the first two terms (i.e., *n* = 1) generate a good estimation of the loaded A-scan.

To assess the robustness of Eq. (6) under more realistic conditions, we performed additional simulations in which the loaded A-scan was corrupted with additive complex Gaussian noise. Specifically, for each clean complex A-scan *A*(*z*), we generated a noisy signal as ${A}_{{{\rm{noise}}}}(z)=A(z)+r\left({n}_{R}(z)+i{n}_{I}(z)\right){{\max }_{z}}| A(z)|$, where *n*_*R*_(*z*) and *n*_*I*_(*z*) are independent standard Gaussian random variables at each axial position *z*. With this convention, the real and imaginary parts of the complex Gaussian perturbation each have standard deviation $r {{\max }_{z}}| A(z)|$. Therefore, the root-mean-square amplitude of the complex perturbation is $\sqrt{2}\,r {{\max }_{z}}| A(z)|$. For example, a noise level *r* = 0.05 means that the real and imaginary components are each scaled by 5% of the maximum magnitude of the clean A-scan, corresponding to a complex root-mean-square perturbation amplitude of approximately 7.1% of ${{\max }_{z}}| A(z)|$. We also modeled vibrational scatterer motion by adding an independent Gaussian displacement to each scatterer after compression, so that the position of the *j*th scatterer became ${\tilde{z}}_{j}^{L}:={z}_{j}^{L}+\delta {z}_{j}^{vibr}$, where $\delta {z}_{j}^{vibr} \sim N(0,{D}_{v}^{2})$, and *D*_*v*_ is the standard deviation of the vibrational displacement. Panels f–j of Fig. 2 show the integral relative error ${{{\mathcal{E}}}}_{2}$, defined as ${{{\mathcal{E}}}}_{2}(X,Y):=\sqrt{\frac{\int_{{\mathbb{R}}}| X(z)-Y(z){| }^{2}dz}{\int_{{\mathbb{R}}}| X(z){| }^{2}dz}}$, between the reference loaded A-scan and the approximation given by Eq. (6) when the series is truncated at *n* = 0, *n* = 1, 2, 3, and 4, respectively. Here, *X*(*z*) and *Y*(*z*) denote the full complex-valued OCT A-scans, rather than their magnitudes, and ∣⋅∣ denotes the complex modulus. In these maps, the level of additive Gaussian noise added to the loaded A-scans varies from 0 to 5%, while the vibrational displacement applied after loading varies from 0 to 10 nm. The black crosses in the top-left corners of panels f and g and the red cross in panel j indicate the noise-free cases and correspond to the A-scans shown in plots b–d, with relative errors of 58%, 33%, and 1.7%, respectively. Likewise, the black cross in the bottom-right corner of panel j corresponds to plot e, where 5% additive complex Gaussian noise is added to the truncated *A*^*L*^ with *n* = 4, together with vibrational scatterer displacement of standard deviation *D*_*v*_ = 10 nm, with relative errors around 17.4%.

It is worth noting that the PHDE assumption is equivalent to approximating the loaded A-scan by the *n* = 0 truncation of Eq. (6), namely ${A}^{L}(z)\approx {\alpha }_{0}(z){A}_{0}^{U}(z)$. In fact, under this assumption, we get $\Delta \phi (z)={{\rm{Arg}}}\{{A}^{U}(z)\overline{{A}^{L}(z)}\}\approx {{\rm{Arg}}}\{{A}^{U}(z)\overline{{\alpha }_{0}(z){A}_{0}^{U}(z)}\}={{\rm{Arg}}}\{\overline{{\alpha }_{0}(z)}\,| {A}^{U}(z){| }^{2}\}=4\pi {\xi }_{0}(D+\hat{\epsilon }z)$, since ∣*A*^*U*^(*z*)∣^2^ is real and non-negative. Therefore, by dividing both sides by 2*k*_0_, we obtain Eq. (1).

We emphasize that equation (6) is theoretically significant and validates the essential building block required by the displacement-estimation method applied to experimental data, as discussed in the “Methods” section.

### Phase-difference approximation in bright speckles

In this section, we derive an approximation of Δ*ϕ* valid in bright speckles by using the definition of ${\phi }^{{\prime} }(z)$ in Eq. (7). Both the numerator and denominator of the resulting ratio are expressed independently through the series representation in Eq. (6), but with different truncation orders: *n* = 1 in the numerator and *n* = 0 in the denominator. This low-order truncation was chosen because it is sufficient to reproduce the leading non-linear terms of Δ*ϕ* beyond the PHDE while keeping the derivation analytically tractable (see Supplementary Note 2 for further details). Higher-order terms would improve the accuracy of the approximation, but they lead to substantially more complicated phase expressions and were not required to demonstrate, theoretically, the existence of these non-linear terms. Nevertheless, higher truncation orders of Eq. (6) are considered later in the manuscript.

In order to calculate Δ*ϕ*, we need to evaluate the derivative of the phase and the magnitude of a complex OCT signal *A*(*z*). Let us consider a complex signal *A*(*z*) ≔ *ρ*(*z*)*e*^*iϕ*(*z*)^, where *ρ*(*z*) and *ϕ*(*z*) are the magnitude and the phase of *A*(*z*), respectively. By assuming that *A*(*z*) is differentiable and *A*(*z*) ≠ 0 (i.e., the region where *ϕ*(*z*) is defined), the derivatives of magnitude and phase are given by^26^:

7 $${\rho }^{{\prime} }(z)=\rho (z)Re\left\{\frac{{A}^{{\prime} }(z)}{A(z)}\right\},\qquad {\phi }^{{\prime} }(z)=Im\left\{\frac{{A}^{{\prime} }(z)}{A(z)}\right\},$$

where $Re\left\{\cdot \right\}$ and $Im\left\{\cdot \right\}$ are the real and imaginary parts, respectively. ${A}^{{\prime} }(z)$ denotes the derivative of the complex OCT signal with respect to the axial coordinate $z\in {\mathbb{R}}$, namely, ${A}^{{\prime} }(z):=\frac{d}{dz}Re\left\{A(z)\right\}+i\frac{d}{dz}Im\left\{A(z)\right\}$.

By integrating the second identity of equation (7), we find an unwrapped and continuous version of *ϕ*(*z*) as:

8 $$\phi (z)=\phi ({z}_{0})+\int^{z}_{{z}_{0}}{\phi }^{{\prime} }(s)ds=\phi ({z}_{0})+\int^{z}_{{z}_{0}}Im\left\{\frac{{A}^{{\prime} }(s)}{A(s)}\right\}ds.$$

Even for experimentally acquired OCT signals, sampled at discrete axial points, the property of *ϕ*(*z*) being continuous and unwrapped is still valid, given that the signal can be arbitrarily sampled in the z-space. This means that equation (8) can be computed with arbitrary accuracy and can be used to calculate an unwrapped phase for experimentally acquired OCT signals. By applying equation (8) for both unloaded and loaded cases we obtain:

9 $$\Delta \phi (z):=\Delta \phi ({z}_{0})+\int^{z}_{{z}_{0}}Im\left\{\frac{\left[{A}^{U}(s)\right]^{\prime} }{{A}^{U}(s)}-\frac{\left[{A}^{L}(s)\right]^{\prime} }{{A}^{L}(s)}\right\}ds,$$

from which we derive an approximation to equation (9) as (see Supplementary Note 2 for further details):

10 $$\Delta \phi (z)\approx C(z)+2{k}_{0}\hat{\epsilon }z+\frac{{k}_{0}\hat{\epsilon }}{{W}^{2}}\frac{\left[{\rho }^{U}(z)\right]^{\prime} }{{\rho }^{U}(z)}+\left(D+\hat{\epsilon }z\right)\left[{\phi }^{U}(z)\right]^{\prime},$$

where *C*(*z*) is a piecewise constant function with discontinuities of ±2*π*, which occur only at the points of minimum amplitude within dark speckles, as explained below.

**Fig. 3:**
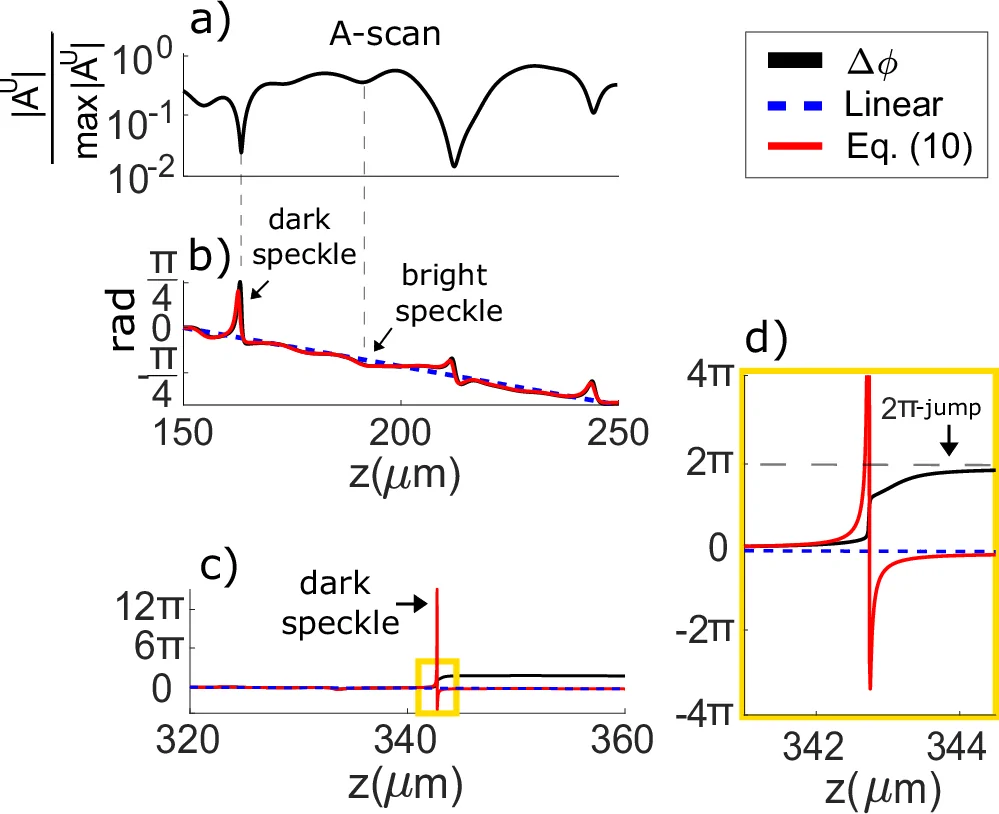
Phase-difference behavior across bright and dark speckles. **a** Normalized magnitude ∣*A*^*U*^∣ of an unloaded simulated A-scan, shown on a log scale. **b** OCT phase difference Δ*ϕ* compared with the linear phase difference predicted for the underlying constant strain using equation (1), and with the approximate form of Δ*ϕ* given by equation (10), along a region containing both dark and bright speckles. **c** Same comparison as in (**b**), shown over an interval containing a dark speckle, illustrating the dark-speckle breakdown of Eq. (10). **d** Magnified view of the boxed region in (**c**).

Figure 3 compares the direct evaluation of Δ*ϕ* with the approximate form of equation (10). Plot b illustrates that the oscillations in Δ*ϕ* are faithfully reproduced by the approximate form in Eq. (10) over regions containing both bright and dark speckles. The corresponding normalized magnitude of the unloaded A-scan is shown on a log scale in plot a. However, plots c and d also show that even this more accurate approximation is less robust in dark speckle, where higher-order non-linear phase behavior may not be faithfully reproduced by Eq. (10). As we clarify below, in dark speckle the non-linear oscillations can reach magnitudes of approximately 2*π* (as shown in plot d). It can be seen from equation (10) that as $\hat{\epsilon }$ and *D* increase, the non-linear terms, which degrade estimations of strain-retrieval methods based on equation (1), also increase.

**Fig. 4:**
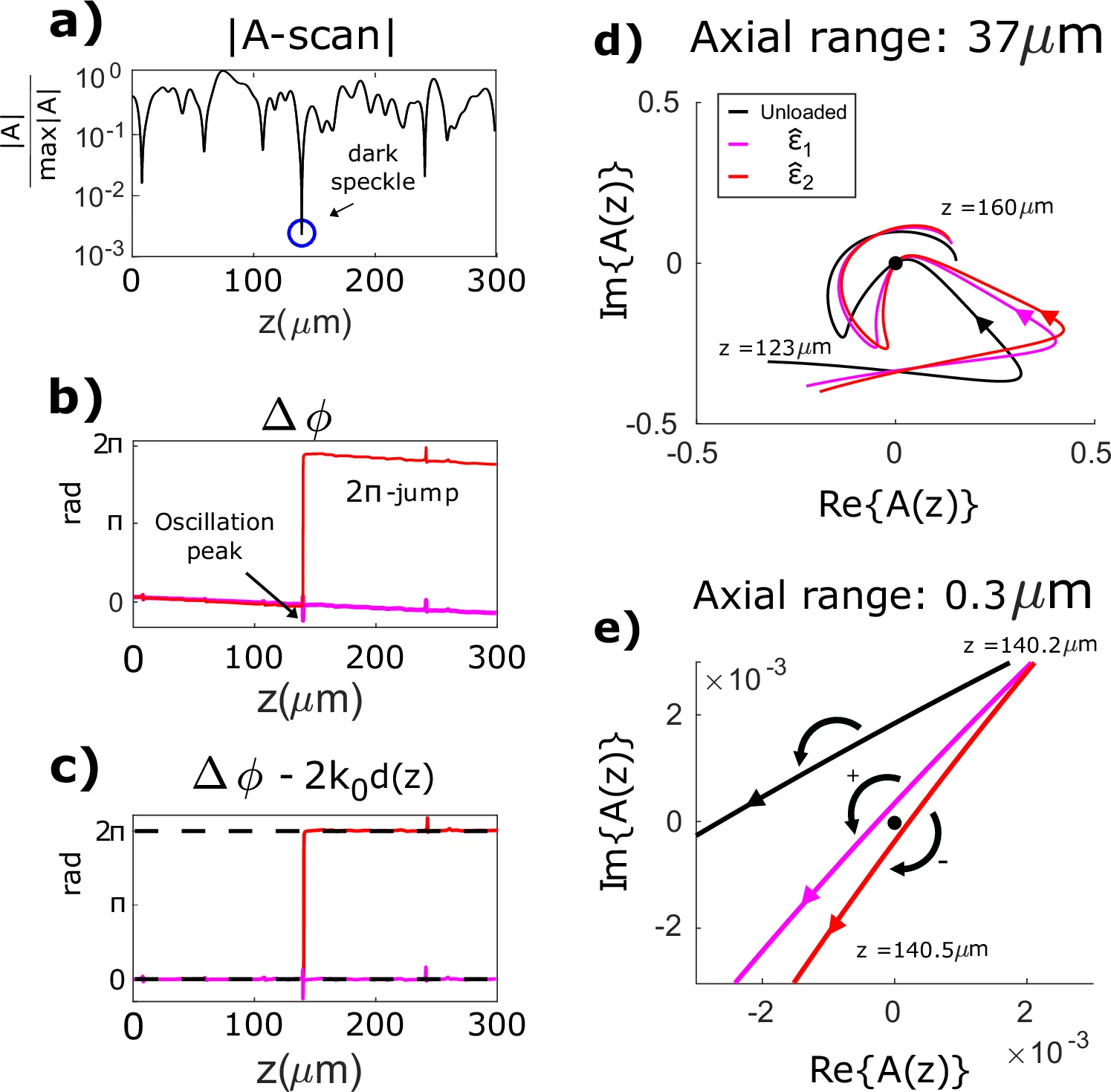
Characterization of oscillation peaks and 2*π*-jumps. Simulated unloaded A-scan *A*^*U*^ and two loaded A-scans, ${A}_{1}^{L}$ and ${A}_{2}^{L}$, corresponding to strains ${\hat{\epsilon }}_{1}=-0.23m\epsilon$ and ${\hat{\epsilon }}_{2}=-0.3m\epsilon$, respectively, with bulk displacements *D*_1_ = *D*_2_ = 0. Here, *mϵ* denotes millistrain. **a** Normalized unloaded A-scan magnitude ∣*A*^*U*^∣ on a log scale, with a dark speckle circled in blue. **b** Plots of Δ*ϕ* for both loaded cases, which show oscillation peaks for ${\hat{\epsilon }}_{1}$ (magenta) and a 2*π*-jump for ${\hat{\epsilon }}_{2}$ (red). **c** Plots of Δ*ϕ* with the expected linear trend in phase difference subtracted, which appears as a piecewise approximately constant function perturbed by the intrinsic oscillations (described in equation (10)), for both the loaded cases. **d** Plot in the complex plane of segments of each A-scan around the dark-speckle region. **e** Magnified representation of plot (**d**) around the origin. The transition from the oscillation peak to the 2*π*-jump occurs because the transition from ${\hat{\epsilon }}_{1}$ to ${\hat{\epsilon }}_{2}$ causes the loaded A-scan trajectory to shift to the other side of the origin.

In the next section, we perform a quantitative analysis of this degradation. The non-linear terms in equation (10) in general perturb OCE strain estimations based on the PHDE. They can be reduced by averaging, as is employed by conventional strain-retrieval methods, such as WLS ^14^ and VM ^6^. Conversely, Δ*ϕ* is chaotic around dark speckles, displaying rapid variation within a span of less than a central wavelength along an A-scan. We now demonstrate that local minima in the magnitude of OCT A-scans correspond to occurrences of what we refer to as oscillation peaks and 2*π*-jumps in the plot of Δ*ϕ*. Figure 4 shows examples of simulated A-scans which exhibit both of these phenomena for one unloaded A-scan and two loaded A-scans, each for different constant strains. The plot in Fig. 4a shows the normalized magnitude of the unloaded A-scan on a log scale. The circled region represents a dark speckle, having a signal ≈3 orders of magnitude lower than that of the average bright speckle. The plot in Fig. 4b shows that Δ*ϕ* is approximately linear for both strains in bright speckle regions, which confirms that the PHDE is a valid approximation in such regions, meaning that perturbations from the PHDE predicted by equation (10) are weak. On the other hand, around dark speckles we see both oscillation peaks (in magenta) and 2*π*-jump perturbations (in red). To better illustrate the 2*π*-jump, Fig. 4c shows Δ*ϕ* plotted with the known underlying linear phase difference, as expected from the PHDE, subtracted. This removes the underlying linear trend in Δ*ϕ*, yielding a roughly constant function with a jump of 2*π* for the second case (red).

To show why this happens, we plotted the three A-scans in the complex plane, parameterized by the axial coordinate *z* in Fig. 4d, e. The plot in Fig. 4d shows the A-scans within the axial interval [123, 160]μm, where the arrows represent the path directions for increasing *z*, and the minimum amplitude in the dark speckle is reached at axial depth *z*_*ds*_ ≈ 140.35 μm. The plot in Fig. 4e shows the 3 A-scans in a narrower axial interval centered at *z*_*ds*_, spanning 300 nm in the axial range [140.2, 140.5]μm of Fig. 4d. Since the plot in Fig. 4e is magnified more than 100 times relative to that of Fig. 4d, the curves appear straight. In this narrow axial range, the loaded phases ${\phi }_{1}^{L}(z)$ and ${\phi }_{2}^{L}(z)$ both undergo an approximate *π* change across the axial interval considered, as they exhibit linear behavior and pass close to the origin. However, the second loaded A-scan (red line) passes on the opposite side of the origin to that of the first loaded case (magenta), and its phase changes direction, so that ${\phi }_{2}^{L}\approx {\phi }_{1}^{L}-2\pi$ for *z* > 140.5 μm. To explain why this happens, let us define the family of curves generated by the loaded A-scans with all real-valued strains, $\hat{\epsilon }$, which we will denote as ${A}^{L}(z;\hat{\epsilon })$. In general, ${A}^{L}(z;\hat{\epsilon })$ is a continuous transformation of the unloaded A-scan with respect to $\hat{\epsilon }$. Let us consider $\left({z}_{0},{\hat{\epsilon }}_{0}\right)$ such that ${A}^{L}({z}_{0};{\hat{\epsilon }}_{0})=0$, then, there exists a small axial region ${I}_{\delta }({z}_{0}):=\left({z}_{0}-\delta,{z}_{0}+\delta \right)$ such that ${\phi }^{L}(z;\hat{\epsilon }):=\rm{Arg} \{{A}^{L}(z;\hat{\epsilon })\}$ exhibits a monotonic increase (decrease) as a function of *z* ∈ *I*_*δ*_(*z*_0_), for each parameter $\hat{\epsilon } < {\hat{\epsilon }}_{0}$, whilst ${\phi }^{L}(z;\hat{\epsilon })$ decreases (increases) as a function of *z* ∈ *I*_*δ*_(*z*_0_) for the subsequent stages of the transformation ($\hat{\epsilon } > {\hat{\epsilon }}_{0}$), resulting in a phase difference of 2*π*.

We now provide a formal definition of these non-linear phenomena in the general case, where *A*^(1)^(*z*) is a complex OCT A-scan and *A*^(2)^(*z*) is a perturbed version of *A*^(1)^(*z*). Such a perturbation may arise, for example, from strain, bulk motion, vibration, or noise. Let *z*_*ds*_ be a local minimum of ∣*A*^(1)^(*z*)∣, and let ${\Omega }_{{z}_{ds}}$ denote a local axial interval centered at *z*_*ds*_ over which the corresponding phase perturbation is analyzed. Let ${\Gamma }_{1}:=\{{A}^{(1)}(z):z\in {\Omega }_{{z}_{ds}}\},\qquad {\Gamma }_{2}:=\{{A}^{(2)}(z):z\in {\Omega }_{{z}_{ds}}\}$ be the associated local trajectories in the complex plane. In practice, ${\Omega }_{{z}_{ds}}$ is chosen as the local interval used to isolate the single phase perturbation associated with the dark-speckle region around *z*_*ds*_. We define the perturbation as a 2*π*-jump if 0 ∈ conv(*Γ*_1_ ∪ *Γ*_2_), where conv(⋅) denotes the convex hull. Otherwise, it is defined as an oscillation peak. In the latter case, its magnitude is defined as ${M}_{I}:={{\max }_{z\in {\Omega }_{{z}_{ds}}}}| \Delta {\phi }_{{{\rm{osc}}}}(z)|$, where Δ*ϕ*_osc_(*z*) denotes the phase difference after subtraction of the local linear trend predicted by the PHDE.

#### Generality of oscillation peaks and 2*π*-jumps in speckled OCT signals

The purpose of this section is to show that the phase-instability mechanism described above is not restricted to quasi-static compression OCE, but can arise broadly in phase-sensitive OCT approaches based on speckled complex OCT signals. Let *A*^*U*^(*z*) and *A*^*L*^(*z*) denote two ideal complex OCT signals used to compute a phase difference in a given modality, such as compression OCE, Doppler OCT, phase-sensitive dynamic OCT, or related approaches in which motion is inferred from phase changes. In a realistic measurement, however, the second complex OCT signal will generally be affected by small perturbations, for example due to stochastic scatterer motion, noise, or other changes occurring between repeated acquisitions. As a result, the measured phase difference is written as

$$\Delta \phi={\phi }^{U}-{\phi }_{{{\rm{pert}}}}^{L}=\left[{\phi }^{U}-{\phi }^{L}\right]+\left[{\phi }^{L}-{\phi }_{{{\rm{pert}}}}^{L}\right].$$

The term $\left[{\phi }^{U}-{\phi }^{L}\right]$ is the ideal phase difference associated with the modality-specific motion, while the term $[{\phi }^{L}-{\phi }_{{{\rm{pert}}}}^{L}]$ is the additional phase contribution caused only by the perturbation. Therefore, even if the ideal phase difference *ϕ*^*U*^(*z*) − *ϕ*^*L*^(*z*) were smooth and contained no oscillation peaks or 2*π*-jumps, the perturbation-induced term ${\phi }^{L}(z)-{\phi }_{{{\rm{pert}}}}^{L}(z)$ can introduce non-negligible additional non-linear phase behavior.

To isolate this effect, we analyze the phase contribution generated only by perturbing one of the two complex OCT signals. In particular, let ${A}_{{{\rm{pert}}}}^{L}(z)$ denote a perturbed version of *A*^*L*^(*z*), with phase ${\phi }_{{{\rm{pert}}}}^{L}(z)$. The perturbation-induced phase difference is then $\Delta {\phi }_{{{\rm{pert}}}}^{L}(z)={\phi }^{L}(z)-{\phi }_{{{\rm{pert}}}}^{L}(z)$. This term is independent of the specific physical model that generated the original pair *A*^*U*^(*z*) and *A*^*L*^(*z*). Thus, if $\Delta {\phi }_{{{\rm{pert}}}}^{L}(z)$ contains oscillation peaks or 2*π*-jumps, these events also appear in the measured phase difference of any phase-sensitive OCT modality in which speckled complex signals are perturbed between acquisitions.

**Fig. 5:**
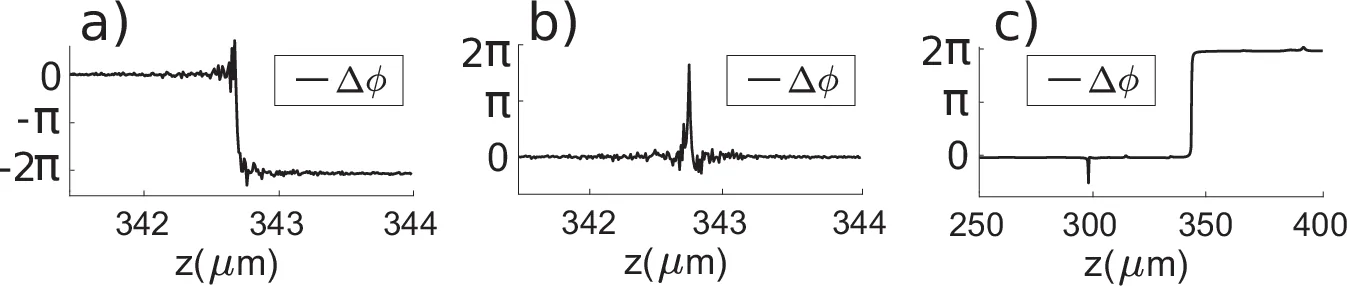
Noise- and vibration-induced oscillation peaks and 2*π*-jumps. Examples of oscillation peaks and 2*π*-jumps generated by small perturbations in the case ${A}_{{{\rm{pert}}}}^{L}$ is a perturbed version of *A*^*L*^. **a** and **b** show Δ*ϕ*_pert_ for two realizations of ${A}_{{{\rm{pert}}}}^{L}$ as 0.1% additive Gaussian noise applied to *A*^*L*^. **c** shows the case of vibrational scatterer motion modeled as a Gaussian displacement with standard deviation *D*_*V*_ = 1 nm. These selected examples show that even very small perturbations can generate non-linear events, including oscillation peaks and 2*π*-jumps.

This is the effect shown in Fig. 5. In plots a and b, we compute $\Delta {\phi }_{{{\rm{pert}}}}^{L}$ for the case ${A}_{{{\rm{pert}}}}^{L}(z)={A}^{L}(z)+n(z)$, where *n*(*z*) is 0.1% additive Gaussian noise. In the absence of this perturbation, $\Delta {\phi }_{{{\rm{pert}}}}^{L}(z)\equiv 0$. However, two different realizations of such a small random perturbation already produce, around the same dark-speckle region at *z* ≈ 342.7 μm, a 2*π*-jump in plot a and an oscillation peak in plot b. Similarly, plot c shows a simulation in which a small vibrational motion, modeled as a stochastic Gaussian displacement of the scatterers ${z}_{j}^{L}$ with standard deviation *D*_*V*_ = 1 nm, was applied. In the same plot, an oscillation peak with *M*_*I*_ ≈ 0.3 × 2*π* is observed at *z* ≈ 298 μm, together with a 2*π*-jump around *z* ≈ 342.7 μm.

These examples show that the perturbation-induced phase term alone can generate oscillation peaks and 2*π*-jumps, even when the perturbation applied to the complex OCT signal is very small. This demonstrates the broad relevance of the mechanism described in the previous section: whenever a speckled OCT signal contains dark speckles, the local complex trajectory can pass close to the origin of the complex plane, where the phase is extremely sensitive to small perturbations. Therefore, these non-linear phase events are not specific to quasi-static compression OCE, but can arise in phase-sensitive OCT modalities in which phase differences are computed from perturbed speckled complex OCT signals. It is important to clarify that, whilst the noise and vibration perturbation analysis performed in this section concerns the broader phase-instability mechanism associated with perturbed speckled complex OCT signals, the series expansion in Eq. (6), the phase-difference approximation in Eq. (10), and the displacement-estimation approach derived below are formulated for OCE-type strain estimation, where local axial motion is represented by axial strain and bulk displacement.

#### Experimental observation of oscillation peaks and 2*π*-jumps

**Fig. 6:**
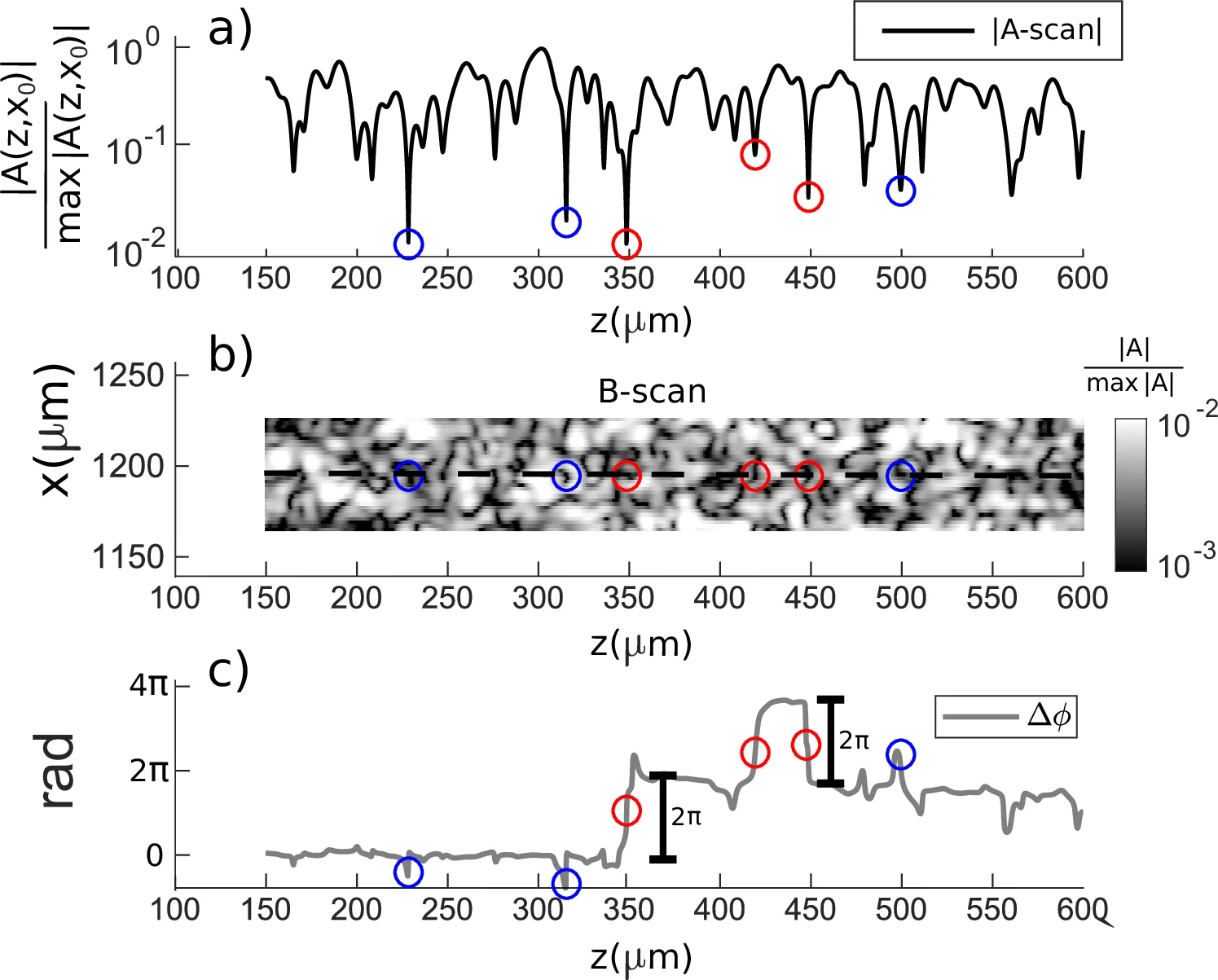
Oscillation peaks and 2*π*-jumps in experimental OCT data. Analysis of unloaded and loaded OCT B-scan of a phantom, normalized with respect to the maximum magnitude of the unloaded B-scan and represented on a log scale. **a** Central unloaded A-scan magnitude, normalized by its own maximum. **b** Unloaded B-scan magnitude, normalized by the global maximum of the B-scan. **c** Corresponding phase difference between the unloaded and loaded central A-scans. Oscillation peaks and 2*π*-jumps are circled in blue and red, respectively.

Figure 6 shows a line plot of experimental data of a homogeneous silicone phantom that exhibits both oscillation peaks and 2*π*-jumps. The OCE experimental data were acquired using the fiber-based spectral-domain OCT system summarized in the “Methods” section and described in detail in ref. ^18^.

The phantom is a 1 mm thick cylinder with a diameter of 10 mm. Titanium dioxide particles with a concentration of 2 mg/mL were evenly mixed with the silicone to enhance OCT SNR. In the experiment, two OCT B-scans were acquired with two levels of mechanical loading, resulting in an average strain estimated as  − 1*mϵ*. Some dark speckles are circled, where blue and red represent oscillation peaks and 2*π*-jumps, respectively. Plot b shows the magnitude of the unloaded OCT B-scan on a log scale, which is composed of 31 A-scans. Plot a in Fig. 6 shows the magnitude of the 16th A-scan, corresponding to the horizontal line of pixels at the center of the B-scan. Plot c shows the phase difference between the two applied loads for the 16th A-scan.

#### Discussion on phase unwrapping in OCE

In this section, we discuss how the PHDE assumption may be impeding research into strain retrieval in OCE by diverting unwarranted attention toward phase unwrapping. This is mainly due to the incorrect assumption that the underlying unwrapped phase is inherently smooth and that only noise makes phase unwrapping unstable. The presence of high oscillation peaks, 2*π*-jumps, and the non-linear terms of Δ*ϕ*, which are all present when the phase is correctly unwrapped, have been considered artifacts of the phase-unwrapping algorithms. This has reinforced the hypothesis that phase unwrapping is one of the main problems facing strain retrieval in OCE. This hypothesis has motivated researchers in the last decade to develop new noise-robust phase-unwrapping algorithms^14,21,22^ or to manipulate Δ*ϕ* to avoid phase unwrapping^6,19^. These approaches have resulted in phase-unwrapping algorithms that tend to smooth Δ*ϕ* in both the axial and lateral directions. This smoothing process has the effect of diminishing the non-linear terms present in equation (10), thus locally linearizing Δ*ϕ* so that it is better represented by equation (1). As a result, strains can be retrieved more accurately by applying equation (1) to the smoothed Δ*ϕ*, compared to applying equation (1) to the correctly unwrapped phase obtained using equation (8). This is because equation (8) causes Δ*ϕ* to be highly non-linear, as has been explained.

This overemphasis on developing algorithms aimed at better smoothing Δ*ϕ* has diverted attention from the fundamental limitations of the PHDE assumption, which we analyze in the next section. To advance OCE strain retrieval, research should prioritize developing algorithms that are more general and make less restrictive assumptions than the PHDE.

### Influence of speckle on VM and WLS strain-retrieval methods

In this section, we qualitatively and quantitatively analyze the accuracy of the VM and WLS strain-retrieval methods and highlight the key limitations of both methods. These limitations derive from the invalidity of the PHDE assumption. A key point to note is that all strain-retrieval methods based on the PHDE share the same limitations.

We simulated 160,801 unloaded A-scans and 180 corresponding loaded A-scans per unloaded signal, covering various strains and displacements, to evaluate strain-retrieval accuracy across depth intervals. Details of the signal generation are provided in the “Methods” section.

From now on, we denote as $B:={A}^{U}\overline{{A}^{L}}$ the complex phase-difference signal. Each axial section of each generated *B*, which will serve as input for both the WLS and VM methods, will be denoted as the OCE signal for simplicity. For the present analysis, OCE signal intervals were divided into three classes: intervals containing at least one 2*π*-jump; intervals without 2*π*-jumps but with oscillation peak magnitude *M*_*I*_ > 0.65 × 2*π*, referred to as the “high peak” class; and intervals without 2*π*-jumps but with *M*_*I*_ < 0.55 × 2*π*, referred to as the “low-peaks” class. Intermediate cases were left unclassified.

**Fig. 7:**
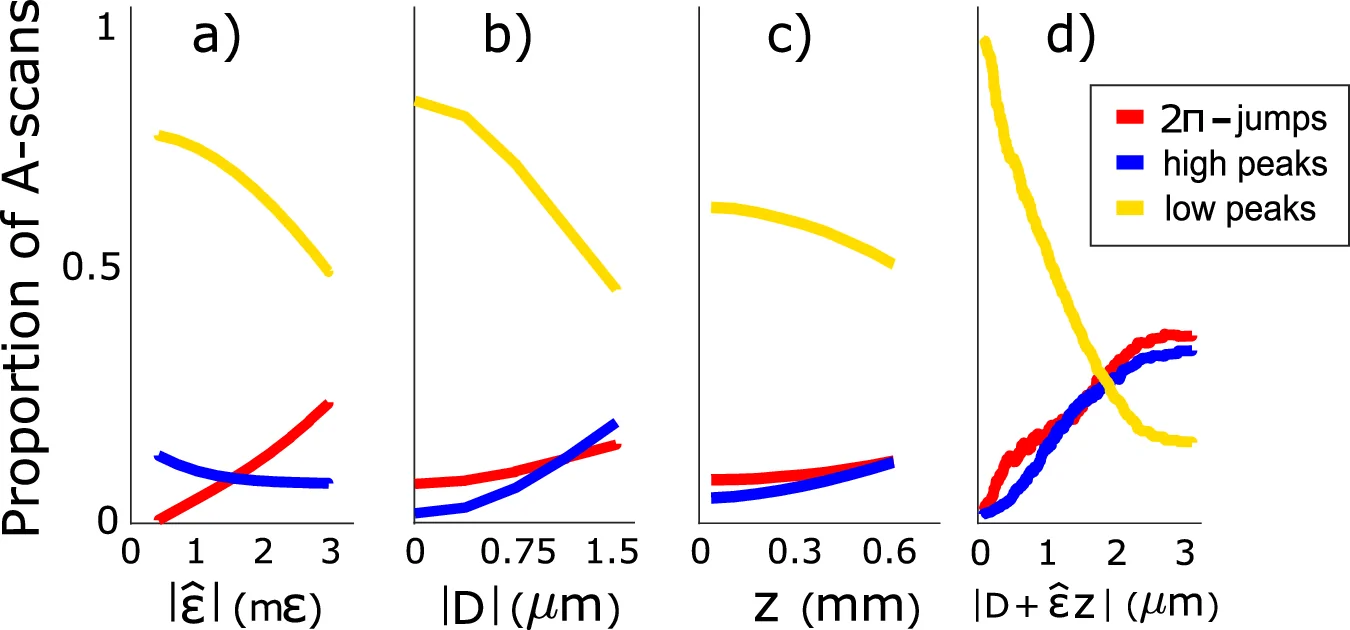
Distribution of OCE signals across displacement parameters. Proportion of OCE signals generated for each class, as a function of $| \hat{\epsilon }|$ (**a**), ∣*D*∣ (**b**), *z* (**c**), and $| D+\hat{\epsilon }z|$ (**d**).

Figure 7 shows plots of the proportion of each OCE signal corresponding to each of the three classes described above, as a function of $\hat{\epsilon }$, *D,* and the depth *z*. For example, all OCE signal intervals related to the compression state $| \hat{\epsilon }|=1m\epsilon$ distribute as 6.5% of 2*π*-jumps, 9.5% of high oscillation peaks, 70% of bright speckle and low oscillation peaks, and the remainder of the OCE signals were unclassified.

Figure 7a reveals a clear trend: as $| \hat{\epsilon }|$ increases, the number of OCE signals containing 2*π*-jumps increases, while the number containing high and low oscillation peaks decreases. This trend is explained by considering the effect of applied strain on *A*^*L*^, plotted in the complex plane (see Fig. 4e). As $| \hat{\epsilon }|$ increases, *A*^*L*^ deviates increasingly from *A*^*U*^, increasing the likelihood of *A*^*L*^ crossing the axis origin, resulting in 2*π*-jumps where previously only oscillation peaks were present.

Figure 7b, however, shows a different trend: as ∣*D*∣ increases, low and high peaks decrease and increase, respectively. However, the number of 2*π*-jumps remains relatively stable and increases only weakly. This is due to the increase in magnitude of non-linear terms of Δ*ϕ* with ∣*D*∣ (see equation (10)), causing low peak OCE signals to be transformed into high peak signals. On the other hand, since there is no change in strain in the plots of Fig. 7b, the curves of *A*^*L*^ in the complex plane (see Fig. 4e) remain unchanged, which means there is no transformation between 2*π*-jumps and oscillation peaks, so the number of OCE signals containing 2*π*-jumps remains roughly constant.

Figure 7c shows a decrease in the number of low peaks with increasing depth *z*, accompanied by an increase in the number of both high peaks and 2*π*-jumps. Figure 7d illustrates that for small displacements, such as $| D+\hat{\epsilon }z| < 0.2\,\mu {{\rm{m}}}$, the majority of OCE signals consist solely of low peaks, without 2*π*-jumps or high oscillation peaks. This strong absence of high oscillation peaks and 2*π*-jumps suggests that non-linear terms of Δ*ϕ* are negligible for small displacements, validating the use of equation (1) as a suitable approximation in such cases. We explore the consequences of this analysis further by considering $| D+\hat{\epsilon }z|=0.1\,{\mathrm{\mu m}}$, with *D* = 0, $\hat{\epsilon }=-1m\epsilon$, and *z* = 100 μm. In this case, 96% of the analyzed OCE signals belong to the low peak class. On the other hand, non-linear terms of equation (10) increase as a function of $| D+\hat{\epsilon }z|$, causing low peaks to transition into high peaks or 2*π*-jumps, which occur in less than 20% of the total number of signals for $| D+\hat{\epsilon }z| > 2\,{\mathrm{\mu m}}$. An example of such a case is given by a uniformly strained sample (*D* = 0) under the strain $\hat{\epsilon }=-3m\epsilon$, at depth *z* = 700 μm, yielding $| D+\hat{\epsilon }z|=2.1\,{\mathrm{\mu m}}$. A second example, for non-uniform strains, is given by $D = -0.5,\mu\mathrm{m}\,, \hat{\epsilon }=-1.5m\epsilon$ and *z* = 1000 μm.

#### Analysis of accuracy of the VM and WLS strain-retrieval methods

We now analyze the accuracy of the WLS and VM strain-retrieval methods. To faithfully represent the optimal implementation of these methods, analysis was performed on OCE C-scans. This allows for adjacent A-scans to be used by each method to enhance the accuracy of retrieved strain, as is done in practice. Details of the signal generation are provided in the “Methods” section.

**Fig. 8:**
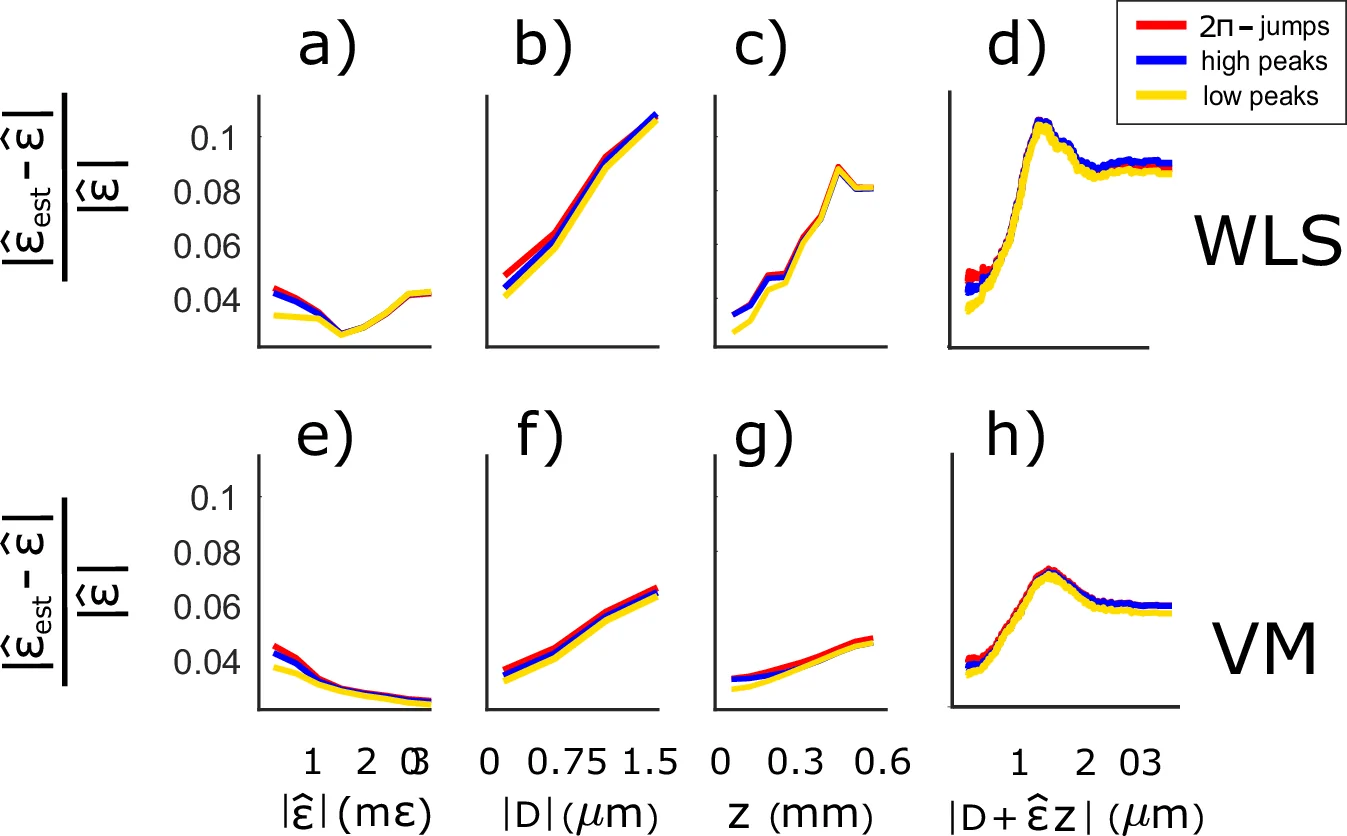
Error trends for WLS and VM strain estimation. Plots of the relative error ${{{\mathcal{E}}}}_{c}$ against $| \hat{\epsilon }|$, ∣*D*∣, *z*, and $| D+\hat{\epsilon }z|$. **a**–**d** show the WLS results for these quantities, respectively, with (**e**)–(**h**) showing the corresponding VM results.

Figure 8 shows a quantitative analysis of the retrieved strains. Each plot illustrates the relative errors of each error array ${{{\mathcal{E}}}}_{c}(\hat{\epsilon },D,z)$, as a function of various parameters. The top plots show the WLS method, while the bottom plots show the VM method. A figure showing relative errors for all values of $\hat{\epsilon }$, *D,* and *z* would be difficult to interpret because several plots would overlap. In particular, several plots exist for each plot of strain error versus $\hat{\epsilon }$, corresponding to different values of *D* and *z*. However, the profile of each of these plots was similar and so for each plot in the first three columns of Fig. 8, we set the non-independent variables as ${\hat{\epsilon }}_{0}=-1m\epsilon$, *D*_0_ = − 0.375 μm and *z*_0_ = 200 μm, so that only one line was plotted for each class. From Fig. 8a, e, we can see that, for $| \hat{\epsilon }| < 1\,m\epsilon$, the error associated with the 2*π*-jumps class slightly exceeds that of the high-peaks class, and both are larger than the error of the low-peaks class. On the other hand, all classes exhibit similar relative errors for $| \hat{\epsilon }| \ge 1\,m\epsilon$. In the low-peaks class, for strains lower than 1 *mϵ* in magnitude, WLS gives a slightly lower minimum error than VM, with minima of approximately 3.5% and 3.7%, respectively. However, for larger strain magnitudes, VM outperforms WLS.

Figure 8b, f shows that ${{{\mathcal{E}}}}_{c}$ increases as a function of ∣*D*∣ for each class, indicating larger strain-estimation errors for samples with non-uniform strain than for uniformly strained samples, corresponding to the case *D* = 0. VM appears more robust for large ∣*D*∣, with maximum errors of approximately 10% and 6.2% for WLS and VM, respectively.

In Fig. 8c, g, both methods perform similarly in the shallow region *z* < 150 μm, and both methods exhibit larger errors for C-scans containing 2*π*-jumps than for the other classes. For *z* > 150 μm, VM outperforms WLS.

Finally, Fig. 8d, h shows that both methods perform well for small local displacement magnitudes $| D+\hat{\epsilon }z|$, with minima of ${{{\mathcal{E}}}}_{c}^{{{\rm{WLS}}}}\approx 0.04$ and ${{{\mathcal{E}}}}_{c}^{{{\rm{VM}}}}\approx 0.0375$. However, for larger values of $| D+\hat{\epsilon }z|$, both methods exhibit increased strain-retrieval error, exceeding 7%. In this regime, VM outperforms WLS, with maximum errors of ${{{\mathcal{E}}}}_{c}^{{{\rm{WLS}}}}\approx 9.4\%$ and ${{{\mathcal{E}}}}_{c}^{{{\rm{VM}}}}\approx 7.2\%$.

Strain-retrieval error increases across all classes, including the low-peaks class, as $| \hat{\epsilon }|$, ∣*D*∣, *z* and $| D+\hat{\epsilon }z|$ increase.

It is worth noting that Fig. 8d, h shows that even when $| \hat{\epsilon }|$ and ∣*D*∣ are individually large, VM and WLS can still perform well if $| D+\hat{\epsilon }z|$ remains small, since $D+\hat{\epsilon }z$ represents the actual displacement at the center of the considered axial interval. The special case $D+\hat{\epsilon }z=0$ also corresponds to zero mean displacement of scatterers randomly drawn from a uniform distribution within a symmetric interval centered at *z*, as shown in Supplementary Note 4. This implies that, around *z*, the unloaded and loaded signals are expected to remain more similar than in cases with larger local mean displacement, which explains why VM and WLS can show higher accuracy in this regime.

Furthermore, in case of large $| \hat{\epsilon }|$, ∣*D*∣, *z* or $| D+\hat{\epsilon }z|$, the low-peaks class does not exhibit a significant reduction in error compared to the high-peaks or 2*π*-jump classes. This underscores that 2*π*-jumps and high-oscillation peaks notably degrade the accuracy of retrieved strains for small displacements, such as small uniform axial strains in shallow regions. In contrast, in scenarios with large displacements or for deep axial regions or large strain discontinuities, the error in both WLS and VM methods is primarily influenced by the non-linear terms of Δ*ϕ*, which are disregarded by conventional methods.

It can be noted that the errors associated with the VM and WLS methods are similar, despite the significant differences between the two methods. This similarity is not surprising, as these errors are primarily due to the shared PHDE assumption. Therefore, to further improve strain-retrieval methods in OCE, it is necessary to take into account the non-linear terms of Δ*ϕ*.

It is important to note that the comparison we made between the two methods is contingent upon the parameters we set for the moving averages and the phase unwrapping. Moreover, this comparison is not exhaustive as it is conducted on ideal, noise-free OCE signals generated using a simplified 1D model of OCT image formation, which neglects some phenomena that could influence strain prediction, such as multiple scattering and signal attenuation. Furthermore, there are additional parameters that warrant consideration when comparing the two OCE models, including image resolution and potential artifacts introduced by the OCE models, such as streak-like artifacts observed in certain WLS images^18^.

### Displacement-estimation approach accounting for phase nonlinearity

In this section, we use Eq. (6) to develop a displacement-estimation method that overcomes the limitations described in the previous section. Here, we summarize key principles of the method, while a full derivation is provided in Supplementary Note 6. At a high level, the method operates directly on the complex OCT signals reconstructed from the measured spectral data, and compares locally deformed versions of the unloaded and loaded A-scans over an axial fitting window.

The displacement estimation is performed locally over axial fitting windows centered at *z*, namely ${\Omega }_{z}=[z-{{\mathcal{T}}}/2,z+{{\mathcal{T}}}/2]$, where ${{\mathcal{T}}}$ denotes the fitting-window width. One strain value and one bulk-displacement value are retrieved for each sampled axial position.

The algorithm starts from the measured OCT spectral data, which are Fourier transformed A-scan by A-scan to obtain the corresponding depth-dependent OCT signals. The Fourier transform is written in terms of the spatial-frequency variable *ξ*, which is Fourier-conjugate to *z*, rather than wavenumber *k*. This choice is consistent with the FFT conventions used in common numerical software, such as MATLAB and Python. In practice, the raw signal is recorded in the spectral domain *I*(*ξ*). In this formulation, *I*(*ξ*) is assumed to have a Gaussian-shaped power spectral density. This assumption is not overly restrictive, as the power spectral density can be reshaped to a Gaussian profile (see Supplementary Note 7.2 for further details), and we have observed heuristically that the proposed displacement-estimation model is robust to this reshaping step. The derivatives of *A*_scan_(*z*) (defined in Eq. (3)) are then computed from *I*(*ξ*) as:

11 $${\partial }_{z}^{n}{A}_{{{\rm{scan}}}}(z)={{{\mathcal{F}}}}^{-1}\left\{{(i4\pi \xi )}^{n}I(\xi )\right\}(2z),\quad n=0,\ldots,4.$$

The Fourier transforms are evaluated at 2*z* because OCT A-scans map the round-trip optical path between the scatterers and the reference mirror of the OCT system. For both the unloaded and loaded acquisitions, the original A-scan and its four derivatives, ${\partial }_{z}^{n}{A}_{{{\rm{scan}}}}(z)$, *n* = 0, …, 4, are then converted into their carrier-removed form using:

12 $${\partial }_{z}^{n}A(z)={e}^{-i4\pi {\xi }_{0}z}\sum_{m=0}^{n}\left(\begin{array}{l}n\\ m\end{array}\right){(-i4\pi {\xi }_{0})}^{n-m}{\partial }_{z}^{m}{A}_{{{\rm{scan}}}}(z),$$

where ${\xi }_{0}=\frac{{k}_{0}}{2\pi }$. These derivatives are then used to reconstruct the generalized terms *A*_*n*_(*z*) appearing in Eq. (6), so that all quantities required for the fourth-order truncation are computed directly from the experimental data (see Supplementary Note 6.1 for details).

Displacement is estimated as a local grid-search optimization problem. For each axial coordinate *z*, the algorithm evaluates a discrete set of trial physical strain values ${\hat{\epsilon }}^{{\prime} }$ and trial bulk displacements ${D}^{{\prime} }$. For each pair $({\hat{\epsilon }}^{{\prime} },{D}^{{\prime} })$, a synthetic deformation is defined as the transformation of the A-scan given by Eq. (6); in practice, this transformation is approximated by truncating the infinite series at *n* = 4. A natural formulation would therefore be to estimate the optimal parameters $({\hat{\epsilon }}^{opt}(z),{D}^{opt}(z))$ by selecting the trial pair that minimizes the integral error $\int_{{\Omega }_{z}}| {A}^{L}(s)-{\tilde{A}}^{U}(s;{\hat{\epsilon }}^{{\prime} },{D}^{{\prime} }){| }^{2}ds$. However, the implemented algorithm uses a more accurate symmetric formulation. Instead of deforming only *A*^*U*^ toward *A*^*L*^, the physical trial deformation $({\hat{\epsilon }}^{{\prime} },{D}^{{\prime} })$ is represented through symmetric parameters (*γ*, Δ), so that a partial deformation (*γ*, Δ) is applied to *A*^*U*^ and the opposite deformation (−*γ*, −Δ) is applied to *A*^*L*^. The two transformed signals are therefore mapped towards a common intermediate configuration. This reduces the deformation applied to each branch and improves the accuracy and robustness of the fourth-order truncation. The corresponding physical strain and bulk displacement are then recovered from (*γ*, Δ) after optimization, as detailed in Supplementary Note 6.2.

Assuming a negative strain $\hat{\epsilon } < 0$, the method synthetically compresses *A*^*U*^(*z*) and stretches *A*^*L*^(*z*) by applying a trial strain *γ* and bulk displacement Δ to *A*^*U*^(*z*) and the opposite pair ( − *γ*, − Δ) to *A*^*L*^(*z*). We denote by ${\widetilde{A}}^{U}(z;\gamma,\Delta )$ and ${\widetilde{A}}^{L}(z;-\gamma,-\Delta )$ the corresponding trial-transformed unloaded and loaded A-scans, respectively. Thus, both ${\widetilde{A}}^{U}(z;\gamma,\Delta )$ and ${\widetilde{A}}^{L}(z;-\gamma,-\Delta )$ are synthetically transformed signals. For each trial pair (*γ*, Δ), the two transformed signals are defined through Eq. (6) and, in practice, approximated as:

13 $${\widetilde{A}}^{U}(z;\gamma,\Delta )\approx \sum_{n=0}^{4}{\alpha }_{n}(z;\gamma,\Delta ){A}_{n}^{U}(z),$$

14 $${\widetilde{A}}^{L}(z;-\gamma,-\Delta )\approx \sum_{n=0}^{4}{\alpha }_{n}(z;-\gamma,-\Delta ){A}_{n}^{L}(z),$$

where the dependence of the coefficients *α*_*n*_ on the strain and bulk-displacement parameters has been written explicitly for clarity (see Supplementary Note 6.4 for details).

In practice, for each *z* and the corresponding *Ω*_*z*_, the algorithm introduces trial symmetric deformation parameters (*γ*, Δ) and defines the global optimum as

15a $${{{\mathcal{E}}}}^{U,L}(z;\gamma,\Delta )=\sqrt{\frac{\int_{{\Omega }_{z}}{\left\vert {\widetilde{A}}^{U}(s;\gamma,\Delta )-{\widetilde{A}}^{L}(s;-\gamma,-\Delta )\right\vert }^{2}\,ds}{\int_{{\Omega }_{z}}{\left\vert {\widetilde{A}}^{L}(s;-\gamma,-\Delta )\right\vert }^{2}\,ds}},$$

15b $$[{\gamma }^{{{\rm{opt}}}},{\Delta }^{{{\rm{opt}}}}]={{\,{\mbox{argmin}}\,}_{(\gamma,\Delta )\in {{\mathbb{R}}}^{2}}}{{{\mathcal{E}}}}^{U,L}(z;\gamma,\Delta )$$

Under the assumption of locally uniform deformation, the corresponding physical strain and bulk displacement are then recovered as

16 $$\begin{array}{r}{\hat{\epsilon }}_{{{\rm{opt}}}}(z)=\frac{2{\gamma }_{{{\rm{opt}}}}(z)}{1-{\gamma }_{{{\rm{opt}}}}(z)},\qquad {D}_{{{\rm{opt}}}}(z)=\frac{2{\Delta }_{{{\rm{opt}}}}(z)}{1-{\gamma }_{{{\rm{opt}}}}(z)}.\end{array}$$

In this way, the optimization compares two locally deformed OCT signals over the full fitting window, rather than matching them at a single axial position. To improve the accuracy beyond the discrete trial grid, each tested pair (*γ*, Δ) is further adjusted using a local linear correction before the residual error is evaluated. This correction is obtained by approximating, to first order, how the residual between the two transformed signals changes for small variations of *γ* and Δ. The corrected candidates are then compared, and the one giving the smallest residual error is selected as the local optimum (see Supplementary Note 6.5 for details).

The estimated global optimum obtained at one search stage is then used to center a narrower trial grid of candidates for (*γ*, Δ), so that the following stage refines that estimate over a smaller region of parameter space. This procedure is repeated over three successive grids of decreasing size, with each stage refining the global estimate obtained at the previous one. The final stage, performed on the finest grid, yields the final estimated parameters (see Supplementary Note 6.3 for more details). In the case of OCT C-scans, the implementation also accounts for lateral displacement after compression. Although the applied loading is intended to produce axial compression, in practice small lateral motions can also occur. These introduce a spatial misalignment between the unloaded and loaded signals, which can reduce the accuracy of their local comparison and thereby affect strain retrieval. To reduce this effect, the algorithm tests the local error ${{{\mathcal{E}}}}_{2}$ after applying different small shifts to the loaded 3D OCT dataset in the *x* and *y* directions. The shift that gives the minimum ${{{\mathcal{E}}}}_{2}$ is selected as the estimated lateral displacement, and the signals are then laterally realigned before the final estimation step (see Supplementary Note 6.2 for details).

Now we demonstrate that this approach does not suffer from the limitations described in the previous section. In particular, we test the proposed method with the same simulated dataset of OCE C-scans that has been described in the “Methods” section.

**Fig. 9:**
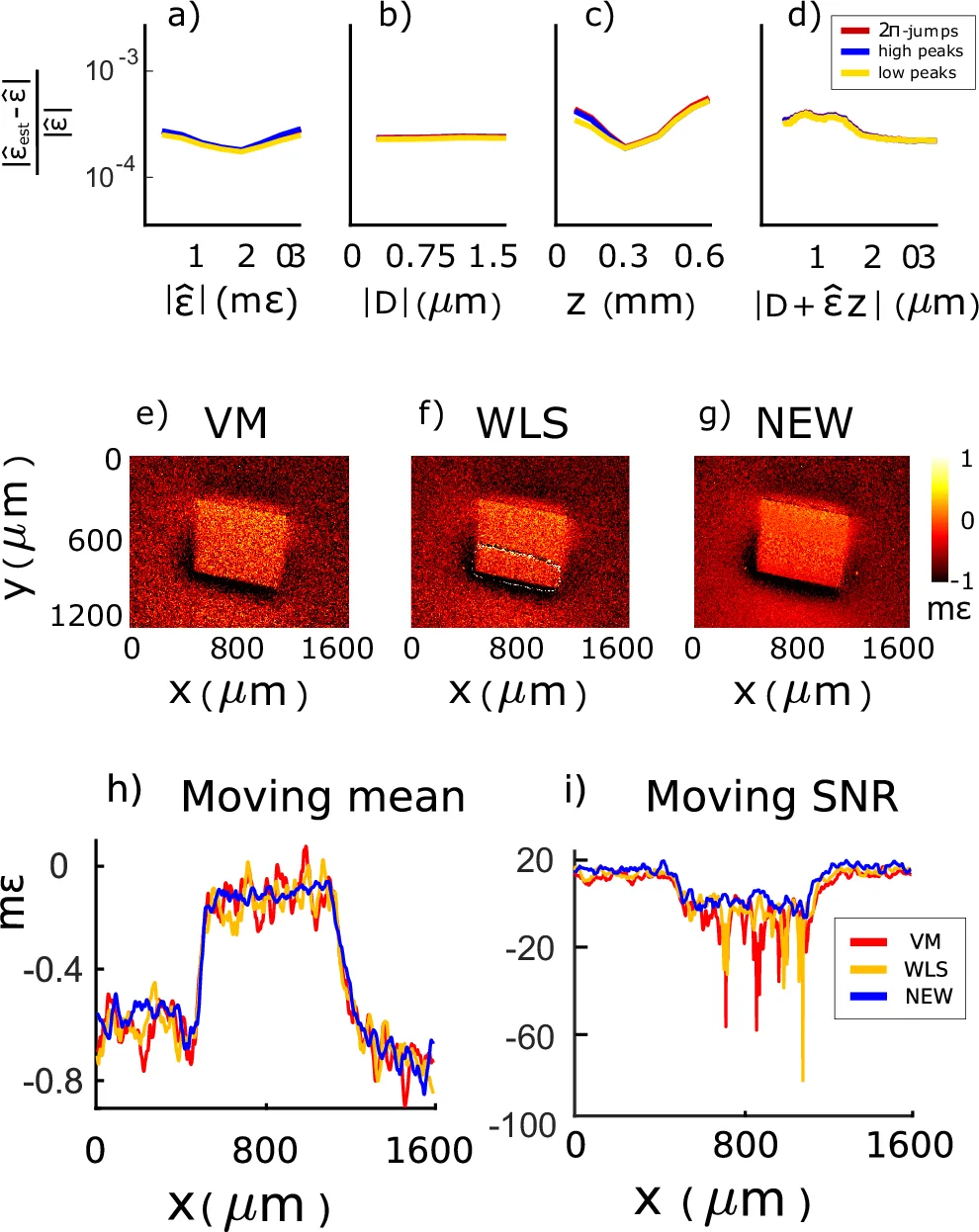
Performance of the proposed displacement-estimation method in simulations and experiments. **a**–**d** Plots of the relative error ${{{\mathcal{E}}}}_{c}$ defined in equation (17), against $| \hat{\epsilon }|,| D|$, *z* and $| D+\hat{\epsilon }z|$, respectively, on a log scale. Strains were estimated by using the proposed displacement-estimation algorithm. **e**–**g** Elastograms of a phantom containing a stiff inclusion, retrieved with VM, WLS, and the proposed method, respectively. Plots **h** and **i** show the moving averages and moving-SNR line profiles, respectively, along the line *y* = 550 μm.

Plots a–d of Fig. 9 show analogous plots to those in Fig. 8, with strains estimated using the proposed displacement-estimation algorithm.

The relative errors between the estimated and actual strains, plotted on a log scale, are approximately two orders of magnitude lower than those calculated with existing methods. To demonstrate its applicability to experimental data, plots e–g of Fig. 9 show en-face elastograms of an inclusion phantom, calculated at a depth of 400 μm from the top surface of the sample. The proposed method reduces noise. To illustrate this more clearly, plots h and i of Fig. 9 show, respectively, the values along the line *y* = 550 μm extracted from the moving-average and moving-SNR maps. In both cases, the underlying maps were computed using a 15 × 15 pixel moving window in the *x*- and *y*-directions on the en-face elastograms shown in panels e–g. Plot i highlights the higher SNR achieved by the proposed method, particularly within the inclusion. Details about the inclusion phantom are provided in the “Methods” section.

It is worth mentioning that the proposed method does not use either lateral or axial averaging to retrieve strain. This may allow it to preserve higher spatial resolution than VM and WLS, for which a 2 × 2 × 2-voxel moving-average filter was applied to each OCE C-scan before strain retrieval.

We emphasize that, although the described method retrieves strain $\hat{\epsilon }$ and bulk displacement *D*, these can be used to estimate the axial displacement *d*(*z*) from the relation $d(z)=D+\hat{\epsilon }z$, which explains why we refer to it as a displacement-estimation method.

These proof-of-principle results show that the proposed approach can provide more accurate and stable strain estimates than conventional phase-difference methods by avoiding their key modeling limitations. However, a detailed analysis of the accuracy, robustness, strengths, and weaknesses of the proposed method is beyond the scope of this article. We intend to present such an analysis in a dedicated future publication.

#### Computational time and scope of the proposed method

The proposed method is computationally more intensive than conventional OCE methods, mainly because it requires repeated fitting of the local deformation parameters within a three-stage coarse-to-fine search. For the implementation used in this work, the computational times required to estimate strain on the inclusion-phantom dataset were 02: 56: 51 for the proposed method, 00: 01: 37 for WLS (phase unwrapping included), and 00: 00: 03 for VM, where the times are reported in hh:mm:ss format, corresponding to hours, minutes, and seconds, respectively.

The raw 3D OCT spectral datasets processed for this timing comparison contained 2048 wavelength samples and 2000 × 1000 lateral positions in the *x* and *y* directions, respectively. Thus, each dataset contained 2000 × 1000 A-scans, each sampled over 2048 spectral points. For visualization only, the images shown in the figures were cropped around the inclusion, displaying a region of 800 × 624 lateral pixels in the *x* and *y* directions, respectively. These results show that the implementation used in this work is considerably slower than the WLS and VM methods, but they also demonstrate that modeling the OCT signals beyond the conventional phase-difference assumption enables more accurate and robust strain estimation.

The main computational bottleneck of the current implementation is the repeated evaluation of the fitting criterion over the trial-parameter grid. Although the current implementation already includes a local linearized correction to improve the accuracy of the tested candidate parameters, this step is not intended to reduce the computational time. The reported runtimes correspond to the current vectorized MATLAB implementations, run on a workstation equipped with two AMD EPYC™ 7453 28-core CPUs. At this stage, we did not investigate a dedicated GPU implementation for any of the methods, since the purpose of this work was to validate the robustness of the proposed formulation rather than to optimize execution time through hardware-specific acceleration. Further acceleration will require both GPU implementation and a semi-analytical and/or approximate treatment of the fitting step, which will be investigated in future work with the aim of considerably reducing the runtime.

## Discussion

Many implementations of label-free and functional modalities such as Doppler OCT and OCE make use of phase-sensitive OCT.

In this paper, we used strain and bulk displacement retrieval in quasi-static compression OCE to show that the well-known phase-difference equation, which has been widely considered to be a mathematical identity, perturbs the measurement of motion using phase-sensitive OCT data. In particular, we have shown that for constant strain, phase difference can differ significantly from the linear function of depth predicted by the phase-difference equation, so that the accuracy and sensitivity of phase-based strain-retrieval models is reduced. We demonstrated two intrinsic types of oscillation in Δ*ϕ* for both bright and dark speckles. We also described the condition under which, in the vicinity of dark speckles, rapid phase changes of 2*π* may occur, which may be erroneously interpreted as noise or a phase-wrapping artifact. This erroneous interpretation has likely diverted research efforts to focus on developing smoothing-oriented phase-unwrapping algorithms, whose accuracy and robustness are, however, constrained by the limitations of the PHDE assumption.

These non-linear terms of Δ*ϕ* also contribute to the degradation of strain estimation quality, especially for large displacements, deep axial regions, or large strain discontinuities. To address this, we derived a more accurate approximation of Δ*ϕ* involving the most significant non-linear terms in equation (10). This enhanced understanding of phase difference can be useful in developing more accurate strain-retrieval models. Building on this enhanced understanding, we developed a method for displacement estimation in OCE that does not rely on equation (1) but instead uses a truncated version of equation (6). Our results demonstrate that this method of strain retrieval is more accurate than existing methods.

We believe that this approach will serve as a foundation for a new generation of more accurate and robust displacement-estimation techniques in OCE-type strain estimation, and that the phase-instability analysis performed in this work enhances understanding of the phase of OCT signals more broadly.

We anticipate that this paper will therefore aid in the further development of a range of modalities based on the phase of OCT signals.

## Methods

### General parameter setting

Throughout all simulations reported in this article, we used realistic OCT parameters. In particular, we assumed a wavelength interval from ${\lambda }_{\min }=1170.5\,{{\rm{nm}}}$ to ${\lambda }_{\max }=1407.8\,{{\rm{nm}}},$ corresponding to a wavelength span of approximately 237 nm. This gives a central spatial frequency ${\xi }_{0}=({\lambda }_{\min }^{-1}+{\lambda }_{\max }^{-1})/2$, or equivalently a central wavenumber *k*_0_ = 2*πξ*_0_ ≈ 4.9 × 10^6^ m^−1^. The corresponding full spectral interval in spatial-frequency space is $B={\lambda }_{\min }^{-1}-{\lambda }_{\max }^{-1}$. The parameter *W* was then chosen using $W=\pi B/\sqrt{\log (1/\tau )}$, where *τ* is the prescribed relative Gaussian level at the interval edges. For these simulations, *τ* = 10^−3^ was used, so that the Gaussian power spectral density was approximately three orders of magnitude lower than its peak value at the spectral edges, yielding *W* ≈ 1.7 × 10^5^ m^−1^ (see Supplementary Note 7.1 for further details).

The scatterers ${z}_{j}^{U}$ were placed randomly along the *z*-axis according to a uniform distribution, with a minimum density corresponding to 11 scatterers per coherence length of the OCT signal. For each scatterer, the scattering cross-section parameter *ρ*_*j*_ was also drawn from a uniform distribution on (0, 1). This choice is consistent with the analysis of Hillman et al.^27^, where OCT speckle formation is related to the effective number of scatterers contributing within the probed region, with strong deviations from fully developed speckle occurring when this number is below about five. In our one-dimensional OCT model, this corresponds to considering the number of randomly distributed scatterers within one coherence-gated axial interval. We numerically verified that approximately the same lower bound is sufficient to generate realistic OCT speckle signals. Therefore, by using 11 scatterers per coherence-gated axial interval as the minimum value, we ensure that the simulated signals are generated in a well-developed speckle regime.

### OCE signal simulation

We generated *N*_*U*_ = 160,801 unloaded OCT A-scans using equation (4a) with random scatterer axial positions, *z*_*j*_, and random scattering cross-section parameters, *ρ*_*j*_, in an axial range of [0,750]μm. We then simulated the loaded OCT signals using equation (4b). We considered *N*_*ϵ*_ = 20 different strains, $\hat{\epsilon }$, equally spaced within the interval [−3, 3]*mϵ*, and *N*_*D*_ = 9 bulk displacements, *D*, equally spaced within the interval [−1.5,1.5]μm, resulting in 180 different *A*^*L*^ for each *A*^*U*^. Then, for each pair $(\hat{\epsilon },D)$ the complex phase difference $B:={A}^{U}\overline{{A}^{L}}$ was calculated. To analyze the accuracy of the WLS and VM methods in retrieving strain, also as a function of depth *z*, we selected *N*_*z*_ = 9 axial intervals of width 130 μm from each *B*. The sampling rate for the OCT signals was *dz* = 3.6 μm, and the axial fitting range employed for both the WLS and VM methods was 100 μm. With these parameter choices, 9 values of axial strain were retrieved for each processed OCE signal. The shallowest axial interval was centered on *z* = 60 μm, with subsequent interval centers separated by 65 μm. The deepest axial interval was centered at *z* = 586 μm. Consequently, for each initial *A*^*U*^, *N*_*ϵ*_ × *N*_*D*_ × *N*_*z*_ = 1620 OCE A-scan intervals were simulated, for a total of *N*_*U*_ × 1620 = 260.5 × 10^6^ OCE A-scan intervals processed by both methods.

Quantifying the error in strain retrieved by the WLS and VM methods, due to the non-linear terms of Δ*ϕ*, would ideally involve testing these methods with OCE signals containing only one of: 2*π*-jumps, oscillation peaks, or bright speckle. However, these three phenomena are interconnected, and furthermore, WLS and VM retrieve pixel strains by utilizing OCE signals from surrounding pixels within a given axial fitting range (typically 100 μm), where the presence of two or three of these phenomena is highly probable. Because of that, simulating OCE signals containing low oscillations in Δ*ϕ* across an entire fitting range is challenging.

Due to the challenges described above, we categorized the simulated sections of OCE signals into three classes: (1) containing at least one 2*π*-jump, (2) without 2*π*-jumps but with a maximum oscillation peak higher than 0.65 × 2*π,* and (3) without 2*π*-jumps but with a maximum oscillation peak lower than 0.55 × 2*π*. The remaining generated OCE intervals were not used as they were too close to the edge of two classes and were not useful for this analysis. The numbers 0.55 and 0.65 were chosen heuristically to ensure that enough OCE signals for each class were generated; however, their choice did not have a significant impact on the results and did not change the conclusions that we made (see Supplementary Note 5 for further details).

### OCE C-scans simulation

In this section, we explain how the simulated OCE C-scans were generated from the OCE signal sections described in the previous section. Specifically, for each OCE signal feature class (i.e., 2*π*-jump, low peak and high peak) and for each $\hat{\epsilon },D$ and *z*, the first 55^2^ OCE signals were selected and were grouped into OCE C-scans comprising 55 lateral A-scans in the *x* and 55 lateral A-scans in the *y* direction, and 9 voxels in the *z* direction. The choice of 55^2^ was heuristic, ensuring that among all *N*_*U*_ axial intervals, the OCE signals simulated for each *D*,* ϵ,* and *z*, there were sufficient in number to form a C-scan for each of the three classes.

Each OCE C-scan was filtered using a (2 × 2 × 2) voxel moving average before strain retrieval with WLS and VM, as is commonly done with these methods to improve the robustness and accuracy of the estimates. In contrast, no averaging was applied before strain retrieval with the proposed displacement-estimation method.

The unwrapping algorithm utilized by the WLS method is described in ref. ^14^. The processing window used for the lateral and axial unwrapping algorithm spanned 4 voxels in *x*, 4 voxels in *y,* and 7 voxels in the *z* direction, respectively. In contrast, the VM uses a second averaging step instead of an unwrapping algorithm, which is performed after the calculation of the interframe phase variation array $C(x,y,z):=B(x,y,z+dz) \overline{B(x,y,z)}$. This second averaging step encompasses 2 voxels in *x*, 2 voxels in *y,* and a number of axial voxels to cover a 100 μm axial fitting range, as described in ref. ^6^. From each assembled OCE C-scan, we filled an error array for WLS and another one for VM, composed by the average relative error of predictions across the C-scan, namely:

17 $${{{\mathcal{E}}}}_{c}(\hat{\epsilon },D,z):=\frac{1}{5{5}^{2}\times 9}\sum_{{i}_{x}=1}^{55}\sum_{{i}_{y}=1}^{55}\sum_{{i}_{z}=1}^{9}\frac{\left\vert {\hat{\epsilon }}_{c}^{est}({i}_{x},{i}_{y},{i}_{z};\hat{\epsilon },D,z)-\hat{\epsilon }\right\vert }{\left\vert \hat{\epsilon }\right\vert },$$

where *c* = 1, 2 or 3 is the class index, $\hat{\epsilon }$ is the known strain, ${\hat{\epsilon }}_{c}^{est}({i}_{x},{i}_{y},{i}_{z};\hat{\epsilon },D,z)$ is the retrieved strain at the voxel corresponding to the spatial indices *i*_*x*_, *i*_*y*_ and *i*_*z*_, for each $\hat{\epsilon },D$, and *z*, and each class *c*. To further clarify the construction described above, each processed C-scan, ${\hat{\epsilon }}_{c}^{est}$, contains strains retrieved using either WLS or VM, corresponding to OCE signals obtained by the same $\hat{\epsilon }$, *D,* and *z*. Ideally, if the retrieved strains were exact, every pixel of each ${\hat{\epsilon }}_{c}^{est}$ would exhibit uniform strain, resulting in a relative error of zero for each pixel.

### Experimental OCE system

The experimental OCE data were acquired using a fiber-based spectral-domain OCT system (Telesto 220; Thorlabs Inc., USA) in a common-path compression configuration, with a central wavelength of 1300 nm, a 3-dB bandwidth of 170 nm, and measured axial and lateral resolutions of 4.8 μm and 7.2 μm, respectively^18^.

### OCE inclusion-phantom construction

The inclusion is a stiff cube (Young’s modulus of 295 kPa) with a side length of 500 μm. The center of the cube is positioned approximately 500 μm below the surface of a softer bulk cylinder (Young’s modulus of 20 kPa) with a thickness of 3 mm and a diameter of 6 mm. Titanium dioxide particles with a concentration of 2 mg/mL and 0.5 mg/mL were mixed with the silicone of the inclusion and bulk, respectively, to enable optical contrast. Each elastogram is centered on the inclusion, spanning 1600 μm in the *x* direction and 1250 μm in the *y* direction. It can be observed that both the VM and WLS elastograms are generally noisier than the elastogram produced by the proposed method. The white streak at the lower part of the inclusion in the WLS elastogram is an artifact resulting from the WLS phase-unwrapping algorithm^18^.

## Supplementary information

Supplementary Information

Transparent Peer Review file

## Data Availability

Most datasets generated in this study, including all data required to reproduce the figures, have been deposited in the Zenodo repository under https://doi.org/10.5281/zenodo.21722824. The full raw experimental OCT dataset, approximately 20 GB in size, has not been deposited because of its large size but is available without restriction by contacting the corresponding author.

## Code availability

The code required to reproduce the figures in this study has been deposited in the Zenodo repository under https://doi.org/10.5281/zenodo.21722824. The repository includes the processed experimental data and the simulated datasets used in the analyses.
