# Supplementary material for: Insight into phase-sensitive optical coherence tomography displacement measurement
Source: Nat Commun. 2026 Sep 16;17:10890. doi: 10.1038/s41467-026-77549-2 (PMC13677722; doi:10.1038/s41467-026-77549-2)
Supplement: Supplementary file 1 [file 41467_2026_77549_MOESM1_ESM.pdf]

Insight into phase-sensitive optical coherence tomography displacement measurement: Supplementary information

Supplementary Note 1. Loaded A-scan as a series expansion of the generalized unloaded A-scans

In this section we rewrite the loaded A-scan as a series expansion of the terms $A_n^U(z)$. In order to do that, we need to rewrite Eq. (4b) of the primary manuscript as follows:

$$\begin{aligned}
A^L(z) :&= \sum_{j=1}^{N} \rho_j e^{-i2k_0\left(D+(1+\hat{\epsilon})z_j^U\right)} e^{-W^2\left(z-\left(D+(1+\hat{\epsilon})z_j^U\right)\right)^2} \\
&= e^{-i2k_0(D+\hat{\epsilon}z)} e^{-W^2(\hat{\epsilon}z+D)^2} \\
&\quad \cdot \sum_{j=1}^{N} \rho_j e^{-i2k_0 z_j^U} e^{-W^2\left(z-z_j^U\right)^2} e^{i2k_0\hat{\epsilon}\left(z-z_j^U\right)} e^{-W^2\left(-2(1+\hat{\epsilon})(\hat{\epsilon}z+D)\left(z-z_j^U\right)+\left(\hat{\epsilon}^2+2\hat{\epsilon}\right)\left(z-z_j^U\right)^2\right)} \\
&= e^{i\psi-W^2 d_0} \sum_{j=1}^{N} \rho_j e^{-i2k_0 z_j^U} e^{-W^2\left(z-z_j^U\right)^2} e^{i2k_0\hat{\epsilon}\left(z-z_j^U\right)} e^{-W^2\left(d_1\left(z-z_j^U\right)+d_2\left(z-z_j^U\right)^2\right)}
\end{aligned} \tag{S1}$$

where the terms $\psi := -2k_0\left(D+\hat{\epsilon}z\right)$, $d_0 := \left(\hat{\epsilon}z+D\right)^2$, $d_1 := -2\left(1+\hat{\epsilon}\right)\left(\hat{\epsilon}z+D\right)$, $d_2 := \left(\hat{\epsilon}^2+2\hat{\epsilon}\right)$ do not depend on the scatterers z_j. By rewriting the last two exponential terms of Eq.(S1) in Taylor series of the exponential function $\left(e^T = \sum\limits_{n=0}^{\infty} \frac{T^n}{n!}\right)$ with argument $T := i2k_0\hat{\epsilon}\left(z-z_j^U\right) - W^2\left(d_1\left(z-z_j^U\right)+d_2\left(z-z_j^U\right)^2\right)$, we get:

$$\begin{aligned}
A^L(z) :&= e^{i\psi-W^2 d_0} \sum_{j=1}^{N} \rho_j e^{-i2k_0 z_j^U} e^{-W^2\left(z-z_j^U\right)^2} e^{i2k_0\hat{\epsilon}\left(z-z_j^U\right)} e^{-W^2\left(d_1\left(z-z_j^U\right)+d_2\left(z-z_j^U\right)^2\right)} \\
&= e^{i\psi-W^2 d_0} \sum_{j=1}^{N} \rho_j e^{-i2k_0 z_j^U} e^{-W^2\left(z-z_j^U\right)^2} \\
&\quad \cdot \sum_{n=0}^{\infty} \frac{\left(i2k_0\hat{\epsilon}\left(z-z_j^U\right) - W^2\left(d_1\left(z-z_j^U\right)+d_2\left(z-z_j^U\right)^2\right)\right)^n}{n!}
\end{aligned} \tag{S2a}$$

$$\begin{aligned}
&= e^{i\psi-W^2 d_0} \sum_{n=0}^{\infty} c_n \sum_{j=1}^{N} \left(z-z_j^U\right)^n \rho_j e^{-i2k_0 z_j^U} e^{-W^2\left(z-z_j^U\right)^2} \\
&= e^{i\psi-W^2 d_0} \sum_{n=0}^{\infty} c_n A_n^U(z)
\end{aligned} \tag{S2b}$$

where c_n are the coefficients of the polynomial terms $\left(z-z_j^U\right)^n$, which can be calculated recursively by expanding the parenthesis in Eq. (S2a), and depend on $z, \hat{\epsilon}$ and D, but *do not depend on the scatterers distribution* z_j. Each term $A_n^U(z)$ can be explicitly calculated from the following recursion:

$$\begin{aligned}
\frac{\partial}{\partial z} A_n^U(z) :&= \frac{\partial}{\partial z}\left(\sum_{j=1}^{N} \left(z-z_j^U\right)^n \rho_j e^{-i2k_0 z_j^U} e^{-W^2\left(z-z_j^U\right)^2}\right) \\
&= \sum_{j=1}^{N} \left(n\left(z-z_j^U\right)^{n-1} \rho_j e^{-i2k_0 z_j^U} e^{-W^2\left(z-z_j^U\right)^2} - 2W^2\left(z-z_j^U\right)^{n+1} \rho_j e^{-i2k_0 z_j^U} e^{-W^2\left(z-z_j^U\right)^2}\right) \\
&= nA_{n-1}^U(z) - 2W^2 A_{n+1}^U(z)
\end{aligned} \tag{S3}$$

By defining $A^U_{-1}(z) \equiv 0$, Eq. (S3) holds also for $n = 0$, which allows to express $A^U_n(z)$ as a function of the derivatives $\partial^k_z A^U_n(z)$. By calling $\alpha_n := e^{i\psi - W^2 d_0} c_n$ we finally get:

$$A^L(z) = \sum_{n=0}^{\infty} \alpha_n A^U_n(z) \tag{S4}$$

1.1 Calculation of the terms $c_n(z)$ and $\alpha_n(z)$

In order to calculate $c_n(z)$ terms, we need to rearrange the terms of the series in Eq. (S2a). In particular:

$$\sum_{n=0}^{\infty} c_n(z) \left(z - z^U_j\right)^n = \sum_{n=0}^{\infty} \frac{\left(i2k_0\hat{\epsilon}\left(z - z^U_j\right) - W^2\left(d_1\left(z - z^U_j\right) + d_2\left(z - z^U_j\right)^2\right)\right)^n}{n!} \tag{S5}$$

In order to simplify the calculations we rewrite Eq. (S5) as:

$$\sum_{n=0}^{\infty} c_n x^n = \sum_{n=0}^{\infty} \frac{\left(ax + bx^2\right)^n}{n!} \tag{S6}$$

where $x := (z - z_j)$, $a := i2k_0\hat{\epsilon} - W^2 d_1$ and $b = -W^2 d_2$ is written without the dependence on z, and we dropped the dependence on z of the terms c_n, for simplicity. The right hand side of Eq. (S6) corresponds to the Taylor expansion of the exponential function evaluated at $ax + bx^2$, which allows us the following calculation:

$$\sum_{n=0}^{\infty} \frac{\left(ax + bx^2\right)^n}{n!} = e^{ax+bx^2} = e^{ax} e^{bx^2} = \left(\sum_{k=0}^{\infty} \frac{(ax)^k}{k!}\right)\left(\sum_{m=0}^{\infty} \frac{\left(bx^2\right)^m}{m!}\right), \tag{S7}$$

The two factors in the last member of Eq. (S7) result from the Taylor expansion of e^{ax} and e^{bx^2}, respectively.
By expanding the product of the two multiplied series, and recollecting all the resulting terms with powers x^n for a given n, which means collecting the resulting terms of the product related to all indices k and m such that $k + 2m = n$ (so that $x^k x^{2m} = x^{k+2m} = x^n$), we find the term $c_n x^n$. The coefficients c_n are then given by:

$$c_n = \sum_{m=0}^{\lfloor \frac{n}{2} \rfloor} \frac{a^{n-2m} b^m}{(n-2m)!m!} \tag{S8}$$

where $\lfloor . \rfloor$ is the floor function. Now, by replacing a and b with the original parameters into Eq. (S8) we get:

$$c_n = \sum_{m=0}^{\lfloor \frac{n}{2} \rfloor} \frac{\left(i2k_0\hat{\epsilon} - W^2 d_1\right)^{n-2m}\left(-W^2 d_2\right)^m}{(n-2m)!m!} \tag{S9a}$$

$$= \sum_{m=0}^{\lfloor \frac{n}{2} \rfloor} \frac{\left(i2k_0\hat{\epsilon} + 2W^2(1+\hat{\epsilon})(\hat{\epsilon}z + D)\right)^{n-2m}\left(-W^2(\hat{\epsilon}^2 + 2\hat{\epsilon})\right)^m}{(n-2m)!m!} \tag{S9b}$$

From Eq. (S9a) it can be seen that, *by construction*, c_n (therefore, also α_n) depend on z, the mechanical loading (parameters $\hat{\epsilon}$ and D) and the physical properties of the OCT system (parameters W and k_0), but *do not depend on the sample*. From Eq. (S9b) we can derive the explicit solution of the terms $\alpha_n = e^{i\psi - W^2 d_0} c_n$:

$$\alpha_n = e^{-i2k_0(\hat{\epsilon}z+D) - W^2(\hat{\epsilon}z+D)^2} \sum_{m=0}^{\lfloor \frac{n}{2} \rfloor} \frac{\left(i2k_0\hat{\epsilon} + 2W^2(1+\hat{\epsilon})(\hat{\epsilon}z + D)\right)^{n-2m}\left(-W^2(\hat{\epsilon}^2 + 2\hat{\epsilon})\right)^m}{(n-2m)!m!} \tag{S10}$$

1.2 Estimation of the order of magnitude of the terms $A^U_n(z)$

The generalized A-scans $A^U_n(z)$ are linear combinations of Gaussian components of the form:

$$g_j(z; n) = (z - z_j)^n \exp\left(-W^2(z - z_j)^2\right), \tag{S11}$$

whose characteristic axial width is of order $1/W$ because the Gaussian in Eq. (S11) is appreciable only when

$$|z - z_j| = O\left(\frac{1}{W}\right),$$

Then, we can say that $g_j(z;n) \sim \frac{1}{W^n} g_j(z;0)$, from which we get:

$$A_n^U(z) \sim \frac{1}{W^n} A^U(z) \tag{S12}$$

Here, the symbol $\sim$ is not intended in a rigorous asymptotic sense. Instead, Eq. (S12) should be interpreted as an order-of-magnitude estimate that holds on average within the fitting range centered in z. In particular, there may be axial locations z' at which $A_n^U(z)$ becomes very small compared to $A_{n+1}^U(z)$, locally contradicting Eq. (S12). This happens because the linear combination of the functions $g_j(z;n)$ undergoes local cancellations.

Although deriving fully rigorous upper bounds for the terms discussed in this section is difficult, the order-of-magnitude estimates reported here were also verified numerically. In particular, the average magnitudes observed across all the simulations performed for this study were consistent with the scalings derived in this section.

Supplementary Note 2. Phase difference estimation in the case of bright speckle

In this section, we derive an estimate of $\Delta\phi$ that is more accurate than the PHDE approximation. To this end, we assume the following orders of magnitude for the relevant parameters:

$$W \sim 10^5\,\mathrm{m}^{-1} \tag{S13a}$$
$$k_0 \sim 10^6\,\mathrm{m}^{-1} \tag{S13b}$$
$$D + \hat{\epsilon} z \sim 10^{-5}\,\mathrm{m} \tag{S13c}$$

To proceed in the calculation of $\Delta\phi$, we need the following differential equation involving the loaded A-scan $A^L(z)$:

$$\begin{aligned}
\left[A^L(z)\right]' &= \partial_z \left(\sum_{j=1}^{N} \rho_j e^{-i2k_0\left(D+(1+\hat{\epsilon})z_j^U\right)} e^{-W^2\left(z-\left(D+(1+\hat{\epsilon})z_j^U\right)\right)^2} \right) \\
&= \sum_{j=1}^{N} \left[-2W^2\left(z-\left(D+(1+\hat{\epsilon})\,z_j^U\right)\right)\right] \rho_j e^{-i2k_0\left(D+(1+\hat{\epsilon})z_j^U\right)} e^{-W^2\left(z-\left(D+(1+\hat{\epsilon})z_j^U\right)\right)^2} \\
&= -2W^2 \sum_{j=1}^{N} \left[\left(z-z_j^U\right)(1+\hat{\epsilon}) - (D+\hat{\epsilon}z)\right] \rho_j e^{-i2k_0\left(D+(1+\hat{\epsilon})z_j^U\right)} e^{-W^2\left(z-\left(D+(1+\hat{\epsilon})z_j^U\right)\right)^2} \\
&= -2W^2\left[(1+\hat{\epsilon})A_1^L(z) - (D+\hat{\epsilon}z)A^L(z)\right]
\end{aligned} \tag{S14}$$

, where
$$\begin{aligned}
A_1^L(z) &:= \sum_{j=1}^{N} \rho_j (z-z_j^U) e^{-i2k_0\left(D+(1+\hat{\epsilon})z_j^U\right)} e^{-W^2\left(z-\left(D+(1+\hat{\epsilon})z_j^U\right)\right)^2} = \\
&= e^{i\psi - W^2 d_0} \sum_{n=0}^{\infty} c_n A_{n+1}^U(z)
\end{aligned} \tag{S15}$$

Eq. (S15) can be derived with analogous calculations that we made in Eq. (S2b). By reversing Eq. (S3) and reminding that $A_{-1}^U(z) \equiv 0, A_0^U(z) \equiv A^U(z)$, we can recursively calculate $A_n^U(z)$ as a function of the derivatives of the unloaded signal $A^U(z)$. The explicit calculation of the first two terms $A_1^U(z)$ and $A_2^U(z)$ is:

$$A_1^U(z) = -\frac{1}{2W^2}\partial_z A^U(z) \tag{S16a}$$
$$A_2^U(z) = \frac{1}{2W^2}A^U(z) + \frac{1}{(2W^2)^2}\partial_z^2 A^U(z) \tag{S16b}$$

Coefficients $c_0 = 1, c_1 = i2k_0\hat{\epsilon} + 2W^2(1+\hat{\epsilon})(\hat{\epsilon}z + D)$ were calculated with Eq. (S9a).

We now derive an approximation of $\Delta\phi$ that reproduces its low-order non-linear terms under the bright-speckle assumption. This approximation is not intended to provide a highly accurate description of $\Delta\phi$, but rather to find a more accurate approximation than PHDE and still sufficiently accurate to reproduce some of its non-linear terms analytically. This reinforces one of the main claims of the manuscript, namely that such non-linear terms are fundamental to OCT signals.

By using Eq. (S4) , Eq.(S14) , Eq. (S15), and Eq.(S16) we can estimate the inner function of the integral in Eq. (9) of the primary

manuscript:

$$
\begin{aligned}
Im\left\{\frac{\partial_s A^U(s)}{A^U(s)}-\frac{\partial_s A^L(s)}{A^L(s)}\right\} &= Im\left\{\frac{-2W^2A_1^U(s)}{A^U(s)}-\frac{-2W^2\left[(1+\hat{\epsilon})A_1^L(s)-(D+\hat{\epsilon}s)A^L(s)\right]}{A^L(s)}\right\}\\
&= Im\left\{-2W^2\left[\frac{A_1^U(s)A^L(s)-A^U(s)\left[(1+\hat{\epsilon})A_1^L(s)-(D+\hat{\epsilon}s)A^L(s)\right]}{A^U(s)A^L(s)}\right]\right\}\\
&= Im\left\{-2W^2\left[\frac{A_1^U(s)A^L(s)-(1+\hat{\epsilon})A^U(s)A_1^L(s)}{A^U(s)A^L(s)}+(D+\hat{\epsilon}s)\right]\right\}\\
&= -2W^2 Im\left\{\frac{A_1^U(s)A^L(s)-A^U(s)\left[(1+\hat{\epsilon})A_1^L(s)\right]}{A^U(s)A^L(s)}\right\}\\
&= -2W^2 Im\left\{\frac{e^{i\psi-W^2d_0}\left(A_1^U(s)\sum_{n=0}^{\infty}c_nA_n^U(s)-(1+\hat{\epsilon})A^U(s)\sum_{n=0}^{\infty}c_nA_{n+1}^U(s)\right)}{A^U(s)A^L(s)}\right\}\\
&= -2W^2 Im\left\{\frac{e^{i\psi-W^2d_0}\left(A_1^U(s)\sum_{n=0}^{\infty}c_nA_n^U(s)-(1+\hat{\epsilon})A^U(s)\sum_{n=0}^{\infty}c_nA_{n+1}^U(s)\right)}{A^U(s)e^{i\psi-W^2d_0}\sum_{n=0}^{\infty}c_nA_n^U(s)}\right\}\\
&\approx -2W^2 Im\left\{\frac{A_1^U(s)\left[c_0A_0^U(s)+c_1A_1^U(s)\right]-(1+\hat{\epsilon})A^U(s)\left[c_0A_1^U(s)+c_1A_2^U(s)\right]}{c_0A^U(s)^2}\right\} &\text{(S17a)}\\
&\approx -2W^2 Im\left\{\frac{c_1\left(A_1^U(s)^2-A^U(s)A_2^U(s)\right)}{c_0A^U(s)^2}\right\} &\text{(S17b)}\\
&= -\frac{1}{2W^2}Im\left\{\frac{c_1\left(\left(\left[A^U(s)\right]'\right)^2-A^U(s)\left(2W^2A_0^U(s)+\left[A^U(s)\right]''\right)\right)}{c_0A^U(s)^2}\right\}\\
&= -\frac{1}{2W^2}Im\left\{\frac{c_1\left(\left(\left[A^U(s)\right]'\right)^2-A^U(s)\left[A^U(s)\right]''\right)}{c_0A^U(s)^2}-\frac{2W^2c_1}{c_0}\right\}\\
&= Im\left\{\frac{1}{2W^2}\frac{c_1}{c_0}\partial_s\left(\frac{\left[A^U(s)\right]'}{A^U(s)}\right)+\frac{c_1}{c_0}\right\}
\end{aligned}
$$

$$
\begin{aligned}
&= Im\left\{\frac{1}{2W^2}\left(i2k_0\hat{\epsilon}+2W^2\left(1+\hat{\epsilon}\right)\left(\hat{\epsilon}s+D\right)\right)\partial_s\left(\frac{\left[A^U(s)\right]'}{A^U(s)}\right)\right.\\
&\qquad\left.+\,i2k_0\hat{\epsilon}+2W^2\left(1+\hat{\epsilon}\right)\left(\hat{\epsilon}s+D\right)\right\}\\
&= 2k_0\hat{\epsilon}+Im\left\{\frac{1}{2W^2}\left(i2k_0\hat{\epsilon}+2W^2\left(1+\hat{\epsilon}\right)\left(\hat{\epsilon}s+D\right)\right)\partial_s\left(\frac{\left[A^U(s)\right]'}{A^U(s)}\right)\right\} \qquad \text{(S17c)}
\end{aligned}
$$

Eq. (S17a) is obtained by approximating the numerator and denominator of the preceding ratio separately. Specifically, Eq. (S4) is truncated at order $n = 1$ in the numerator and at order $n = 0$ in the denominator. These relatively severe truncations were adopted in order to obtain a closed-form expression, which could not be derived for more accurate truncation orders. An additional approximation is introduced in Eq. (S17b), where the factor $(1 + \hat{\epsilon})$ is approximated as 1, since this term does not significantly affect the approximation. However, as explained in the section **Mapping between loaded and unloaded A-scans** of the main manuscript, this truncation choice is still more accurate than the PHDE, which corresponds to the $n = 0$ truncation in both the numerator and denominator. For this reason, the approximation still has sufficient fidelity to reproduce fundamental non-linear oscillations of $\Delta\phi$ that deviate from the PHDE, as is shown

in Figure (3) of the main manuscript. By replacing Eq. (S17c) in Eq. (9) of the primary manuscript, we have:

$$\begin{aligned}
&\Delta\phi(z) = \\
&= \Delta\phi(z_0) + \int_{z_0}^{z} \left(2k_0\hat{\epsilon} + Im\left\{ \frac{1}{2W^2}\left(i2k_0\hat{\epsilon} + 2W^2\left(1+\hat{\epsilon}\right)\left(\hat{\epsilon}s + D\right)\right)\partial_s\left(\frac{\left[A^U(s)\right]'}{A^U(s)}\right)\right\}\right) ds \\
&= \Delta\phi(z_0) + \left[2k_0\hat{\epsilon}s + \frac{k_0\hat{\epsilon}}{W^2} Re\left\{\frac{\left[A^U(s)\right]'}{A^U(s)}\right\} + \left(1+\hat{\epsilon}\right)\left(D+\hat{\epsilon}s\right) Im\left\{\frac{\left[A^U(s)\right]'}{A^U(s)}\right\}\right]_{z_0}^{z} \\
&\quad + \left[-\hat{\epsilon}\left(1+\hat{\epsilon}\right)\right]\int_{z_0}^{z} Im\left\{\frac{\left[A^U(s)\right]'}{A^U(s)}\right\} ds && \text{(S18a)} \\
&= C + 2k_0\hat{\epsilon}z + \frac{k_0\hat{\epsilon}}{W^2} Re\left\{\frac{\left[A^U(z)\right]'}{A^U(z)}\right\} + \left(D+\hat{\epsilon}z\right) Im\left\{\frac{\left[A^U(z)\right]'}{A^U(z)}\right\} - (\hat{\epsilon}+\hat{\epsilon}^2)\phi_U(z) && \text{(S18b)} \\
&\approx C + 2k_0\hat{\epsilon}z + \frac{k_0\hat{\epsilon}}{W^2}\frac{\rho'_U(z)}{\rho_U(z)} + \left(D+\hat{\epsilon}z\right)\phi'_U(z), && \text{(S18c)}
\end{aligned}$$

where C is a constant depending on z_0. The last term of Eq. (S18b) was neglected heuristically. Indeed, since $\Im\left\{\frac{[A^U(z)]'}{A^U(z)}\right\} = \phi'_U(z)$, the term $(D+\hat{\epsilon}z)\phi'_U(z)$ depends on the instantaneous value of $\phi'_U(z)$, and therefore retains its sharp local oscillations. Because $\phi'_U(z)$ undergoes rapid positive and negative fluctuations, substantial cancellations occur in its integral, making $\phi_U(z)$ much smoother and typically smaller than the local term $(D+\hat{\epsilon}z)\phi'_U(z)$. In addition, the neglected term is multiplied by the small prefactor $-(\hat{\epsilon}+\hat{\epsilon}^2)$. For this reason, it acts mainly as a weak smooth correction and was neglected, while the term $(D+\hat{\epsilon}z)\phi'_U(z)$ was retained since it is responsible for the oscillatory behaviour of interest. This behavior was also verified numerically in all the simulations considered here.

Supplementary Note 3. Description of the bulk displacement D

By assuming a sample consisting of N+1 layers of uniform strains defined by intervals $I_p := [L_p, L_{p+1}]\,|_{p=0:N}$:

$$\begin{aligned}
&s(z_j) = s(0) + z_j(1+\hat{\epsilon}_0), && 0 \le z_j \le L_1; \\
&s(z_j) = s(L_1) + (z_j - L_1)(1+\hat{\epsilon}_1), && L_1 < z_j \le L_2; \\
&\ldots && \ldots \\
&s(z_j) = s(L_N) + (z_j - L_N)(1+\hat{\epsilon}_N), && L_N < z_j \le L_{N+1}; && \text{(S19)}
\end{aligned}$$

where $\hat{\epsilon}_p$ is the uniform strain in the p-th layer I_p, and $s(z_j)$ is the new position of z_j after the loading and $L(0) = 0$. In the first layer of uniform strain there is the initial displacement of scatterers placed at the reference axial coordinate $z = 0$, which assuming a common path OCT system, is equal to 0 ($s(0) = 0$).

By rearranging the terms of Eqs. (S19) we can rewrite the displacement of the scatterers in each layer I_p:

$$s(z_j) = D_p + z_j(1+\hat{\epsilon}_p) \quad \forall z_j \in I_p, \qquad \forall p = 1, ..., N, \tag{S20}$$

where $D_p = s(L_p) - L_p(1+\hat{\epsilon}_p)$ is called **bulk displacement**. D_p is the difference between the actual displacement of L_p and the displacement that L_p would have if all the previous layers were under the strain $\hat{\epsilon}_p$. In case of single uniform strain, $D_p = 0$.

We can describe the bulk displacement D_N of the layer $[L_N, L_{N+1}]$ in terms of the average strain of the previous layers $<\hat{\epsilon}>_N := \frac{1}{L_N}\sum_{p=0}^{N-1}(L_{p+1}-L_p)\,\hat{\epsilon}_p$. In fact, we can rewrite D_N as:

$$\begin{aligned}
D_N &= s(L_N) - L_N(1+\hat{\epsilon}_N) \\
&= s(L_{N-1}) + (L_N - L_{N-1})\,(1+\hat{\epsilon}_{N-1}) - L_N(1+\hat{\epsilon}_N) \\
&= s(L_{N-2}) + (L_N - L_{N-1})\,(1+\hat{\epsilon}_{N-1}) + (L_{N-1} - L_{N-2})\,(1+\hat{\epsilon}_{N-2}) - L_N(1+\hat{\epsilon}_N) \\
&\ldots \\
&= \sum_{p=0}^{N-1}(L_{p+1}-L_p)\,(1+\hat{\epsilon}_p) - L_N(1+\hat{\epsilon}_N) \\
&= L_N + \sum_{p=0}^{N-1}(L_{p+1}-L_p)\,\hat{\epsilon}_p - L_N - L_N\hat{\epsilon}_N \\
&= L_N <\hat{\epsilon}>_N - L_N\hat{\epsilon}_N && \text{(S21)}
\end{aligned}$$

The two terms subtracted in Eq. (S21) are the real displacement $\left(L_N < \hat{\epsilon} >_N \right)$ and the displacement that there would be in case of uniform strain $\hat{\epsilon}_N$ across the entire sample $\left(L_N \hat{\epsilon}_N \right)$.

Supplementary Note 4. Meaning of zero displacement of scatterers

In this section we demonstrate that the average displacement of uniformly distributed scatterers related to the axial interval centered in z, with loading related to a zero displacement $|D + \hat{\epsilon} z| = 0$, is zero.

Let us call $2L$ the range of the axial interval. We want to calculate the mean of the function $S(x) := D + \hat{\epsilon}(z + x)$, such that $D + \hat{\epsilon} z = 0$, and $x \in [-L, L]$. Such mean can be calculated by:

$$< S(x) >= \frac{1}{2L} \int_{-L}^{L} S(x) dx = \frac{1}{2L} \int_{-L}^{L} \left(D + \hat{\epsilon}(z + x) \right) dx = \hat{\epsilon} \frac{1}{2L} \int_{-L}^{L} x dx = 0 \tag{S22}$$

Supplementary Note 5. Lower and upper bounds for the statistical analysis of WLS and VM methods

In this section we provide more details on the setting of the numbers 0.55 and 0.65, related to the analysis performed in *OCE signal simulation* subsection of the **Methods** section of the primary manuscript.

The value $N_1 := 0.55$ serves as the upper threshold for OCE signals falling into class (3). Through our analysis, we observed that setting $N_1 < 0.55$ made it nearly impossible to generate OCE signals satisfying $|D + \hat{\epsilon} z| > 2\mu$m.

Regarding the lower threshold of class (2), $N_2 := 0.65$ was chosen to exclusively encompass 'high' oscillation peaks. Opting not to set $N_2 = 0.55$, which would have maintained continuity with the class of low oscillation peaks, was intentional. Doing so would have diminished the distinction between class (2) and class (3). Instead, our aim was to underscore how high oscillation peaks influence prediction compared to low oscillation peaks. Thus, setting the lower bound N_2 as high as possible was preferred. Similar to N_1, the choice of N_2 was heuristic, ensuring sufficient generation of OCE signals for each compression scenario.

We emphasize that we conducted numerous tests with various combinations of N_1 and N_2, and the plots in Fig. (7) and Fig. (8) of the main document remained largely consistent across these combinations. Consequently, the explanations provided alongside the figures and the conclusions drawn from them remain unchanged.

Supplementary Note 6. Implementation details of the new displacement estimation method

This section describes in detail the implementation of the new displacement estimation method introduced in the main manuscript.

6.1 Derivation of the generalized A-scans from the spectral data. This section explains how the generalized A-scans $A_n^U(z)$ and $A_n^L(z)$ are obtained from the spectral-domain signal. In what follows, the Fourier transform is expressed using the spatial-frequency variable ξ, which is Fourier-conjugate to z, rather than the wave number k. This choice is consistent with the FFT conventions used in common numerical software, such as MATLAB and Python. For simplicity, the derivation is presented for a generic signal $A(z)$; the same relations apply to both the unloaded and loaded cases, namely $A^U(z)$ and $A^L(z)$.

The algorithm employs Eq. (S4) truncated at $n = 4$, and therefore requires the computation of the generalized A-scans $A_n(z)$. These terms are not obtained directly from the measured spectrum. Instead, they are reconstructed from the derivatives $\partial_z^k A(z)$, with $k = 0, \ldots, n$, through the recursive relation in Eq. (S3). It is therefore important to distinguish between the quantities that are directly accessible from the spectral data and those required by the algorithm. In particular, the function $A(z)$ appearing in $A_n(z)$ denotes the carrier-removed A-scan, defined as follows (see Eqs. (4) of the main manuscript):

$$A(z) = e^{-i4\pi\xi_0 z} A_{\text{scan}}(z) \tag{S23}$$

where $\xi_0 = \frac{k_0}{2\pi}$. Accordingly, the derivatives $\partial_z^k A(z)$ required to compute $A_n(z)$ are the derivatives of the carrier-removed A-scan, not of $A_{\text{scan}}(z)$. However, in practice, the directly accessible quantity is the measured spectrum $I(\xi)$, from which one can reconstruct $A_{\text{scan}}(z)$

and its derivatives, but not $\partial_z^k A(z)$ directly. However, we can express the derivatives of $A(z)$ in terms of the derivatives of $A_{\text{scan}}(z)$, by differentiating Eq. (S23) n times:

$$\partial_z^n A(z) = e^{-i4\pi\xi_0 z} \sum_{m=0}^{n} \binom{n}{m} (-i4\pi\xi_0)^{\,n-m} \partial_z^m A_{\text{scan}}(z), \tag{S24}$$

To prove Eq. (S24), let us consider a sufficiently smooth function $h(z)$. One has $\partial_z(e^{az}h(z)) = e^{az}h'(z) + ae^{az}h(z) = e^{az}(\partial_z + a)h(z)$. Differentiating n times gives $\partial_z^n(e^{az}h(z)) = e^{az}(\partial_z + a)^n h(z)$. Since a is a constant, the operator ∂_z and the multiplication by a commute, so the binomial theorem applies: $(\partial_z + a)^n = \sum_{m=0}^{n} \binom{n}{m} a^{\,n-m} \partial_z^m$. Hence $\partial_z^n(e^{az}h(z)) = e^{az}(\partial_z + a)^n h(z) = e^{az} \sum_{m=0}^{n} \binom{n}{m} a^{\,n-m} \partial_z^m (h(z))$. By setting $a = -i4\pi\xi_0$ and $h(z) = A_{scan}(z)$, we obtain Eq. (S24). For example, the first two derivatives are:

$$\partial_z A(z) = e^{-i4\pi\xi_0 z} \left[(-i4\pi\xi_0) A_{\text{scan}}(z) + \partial_z A_{\text{scan}}(z) \right], \tag{S25a}$$

$$\partial_z^2 A(z) = e^{-i4\pi\xi_0 z} \left[(-i4\pi\xi_0)^2 A_{\text{scan}}(z) + 2(-i4\pi\xi_0)\partial_z A_{\text{scan}}(z) + \partial_z^2 A_{\text{scan}}(z) \right]. \tag{S25b}$$

Higher-order derivatives follow directly from Eq. (S24). The derivatives of $A_{\text{scan}}(z)$ are then reconstructed from the measured spectrum according to:

$$\partial_z^n A_{\text{scan}}(z) = \mathcal{F}^{-1} \left\{ (i\,4\pi\xi)^n I(\xi) \right\} (2z), \quad n = 0, \ldots, 4. \tag{S26}$$

Therefore, the numerical procedure is as follows. First, the derivatives $\partial_z^n A_{\text{scan}}(z)$ are reconstructed from the measured spectrum $I(\xi)$ using Eq. (S26). Second, Eq. (S24) is used to obtain the derivatives $\partial_z^n A(z)$ of the carrier-removed A-scan. Finally, the generalized A-scans $A_n(z)$ required by the algorithm are computed from these derivatives through the recursive relation in Eq. (S3). In particular, setting $n = 0$ gives $A_1(z)$. Differentiating $A_1(z)$ then gives $\partial_z A_1(z)$, which is inserted back into the same relation with $n = 1$ to obtain $A_2(z)$. Repeating this procedure yields all terms up to the required order. For $n = 0, 1, 2, 3$ and 4, the resulting expressions are:

$$A_1(z) = -P\,\partial_z A(z), \tag{S27a}$$

$$A_2(z) = P\left(A(z) + P\,\partial_z^2 A(z) \right), \tag{S27b}$$

$$A_3(z) = -P^2\left(3\,\partial_z A(z) + P\,\partial_z^3 A(z) \right), \tag{S27c}$$

$$A_4(z) = P^2\left(3\,A(z) + 6P\,\partial_z^2 A(z) + P^2\,\partial_z^4 A(z) \right). \tag{S27d}$$

where $P := (2W^2)^{-1}$. Hence, all terms required by the fourth-order truncation of Eq. (S4) are ultimately reconstructed from the measured spectral-domain data.

6.2 Implementation of local deformation estimation with lateral displacement retrieval. Having derived the generalized A-scans from the spectral-domain data, we now describe how they are used in the numerical implementation of the proposed method. The estimation is performed locally over axial fitting windows of fixed length, each centered at a discrete depth position z, so that one strain and one bulk-displacement are retrieved for the fitting range $\Omega_z = [z - \mathcal{T}/2, z + \mathcal{T}/2]$ centered at z, where $\mathcal{T}$ is the width of the fitting range. In particular, for each Ω_z, the algorithm evaluates a discrete set of trial strains $\hat{\epsilon}'$ and trial bulk displacements D'. Each candidate pair $(\hat{\epsilon}', D')$ defines a *synthetic deformation* of the carrier-removed A-scans through Eq. (S4), truncated at $n = 4$. The optimal estimates, $\hat{\epsilon}^{opt}(z)$ and $D^{opt}(z)$, are then obtained by selecting the trial pair that minimizes the integrated squared error between the transformed signals over Ω_z.

In real experiments, mechanical loading generally induces lateral motion in addition to axial deformation. To describe this extension, we consider three-dimensional OCT acquisitions (C-scans) with voxels $\mathcal{V} := [i, j, k]$, where i, j and k denote the indices along the x, y and z directions, respectively. From this point onward, we therefore assume OCT C-scans have been acquired, so that neighbouring A-scans in both lateral directions can be taken into account. A-scans and B-scans are recovered as special cases by assuming zero motion along the missing lateral directions. Under mechanical loading, the portion of tissue occupied by the voxel $\mathcal{V}$ before loading, is typically displaced by a 3D bulk displacement vector $\boldsymbol{\delta}_{\mathcal{V}} := [\delta_i, \delta_j, \delta_k]$ after loading, such that $A^U[i, j, k]$ corresponds to $A^L[i + \delta_i, j + \delta_j, k + \delta_k]$ rather than $A^L[i, j, k + \delta_k]$. Since a C-scan $A[i, j, k]$ represents a sampled version of a continuous 3D array $A(x, y, z)$, the displacement components δ_i, δ_j and δ_k are general non-integer values. This means that the portion of tissue corresponding to the voxel $\mathcal{V}$ after loading typically moves to a region of space that lies between voxels. To reduce the complexity of the lateral displacement estimation algorithm, we assume that δ_i and δ_j are integers, allowing them to be treated as indices of voxels. In contrast, as also described in the main manuscript, since OCT A-scans axial sampling step size can be arbitrarily adjusted using zero-padding in the FFT, we treat both k and δ_k as a real number, namely, to simplify notation we replace the discrete variable k with the continuous variable z. Similarly, the axial displacement component δ_k is replaced by the axial bulk displacement D'. Under these assumptions, let us denote by $\widetilde{A}(i, j, z; \hat{\epsilon}', D')$ the synthetic transformation obtained by applying Eq. (S4) to the C-scan $A(i, j, z)$ for a deformation specified by $\hat{\epsilon}'$ and D'. A natural first formulation of the problem is then to compare the loaded A-scan with a synthetically deformed version of the corresponding unloaded one. Because lateral motion changes the correspondence between lateral indices before and after loading, the axial correlation between A^U and A^L around $\mathcal{V}$ is reduced compared with the case of purely axial displacement, which degrades the accuracy of the deformation estimation.

To account for this, one may test multiple lateral shifts (δ_i, δ_j) of the loaded A-scan and identify the pair that gives the best agreement with the synthetically deformed unloaded A-scan. On this basis, for each axial depth z and lateral indices (i, j), the optimal parameters $(\delta_i^{opt}, \delta_j^{opt}, \hat{\epsilon}^{opt}, D^{opt})$ could be defined by minimizing $\int_{\Omega_z} \left|\widetilde{A}^U(i, j, s; \hat{\epsilon}', D') - A^L(i + \delta_i, j + \delta_j, s)\right|^2 ds$, that is, by searching over trial values of $\hat{\epsilon}'$, D', δ_i, and δ_j for the combination that best matches the unloaded and loaded signals.
Although this formulation is natural, in practice it is not the one used in the numerical implementation. Instead of deforming only A^U and comparing it directly with A^L, the algorithm applies trial synthetic deformations to both A^U and A^L, thereby mapping the two signals toward an intermediate configuration. This symmetric formulation reduces the effective deformation applied to each signal and, as a result, improves the accuracy of the truncated Taylor approximation used by the algorithm. The underlying idea is that, if there is a strain $\hat{\epsilon}$ and a bulk displacement D transforms $A^U(i, j, z)$ into $A^L(i, j, z)$, then there must also exist a pair (γ, Δ) such that applying the deformation (γ, Δ) to A^U and the opposite deformation $(-\gamma, -\Delta)$ to A^L maps both signals to the same intermediate A-scan. To formalize this idea, it is sufficient to show that the two transformations map *each* scatterer in Ω_z to the same intermediate axial coordinate. Let z_n^U denote the unloaded axial coordinate of the nth scatterer associated with the A-scan $A^U(i, j, z)$, and let z_n^L denote its corresponding loaded axial coordinate associated with $A^L(i, j, z)$. In other words, scatterers at positions z_n^U and z_n^L within the fitting window Ω_z generate $A^U(i, j, z)$ and $A^L(i, j, z)$, respectively.

This can be expressed by writing explicitly the physical and symmetric coordinate transformations. Within each fitting window, under the local-deformation approximation, the relation between the physical deformation parameters $(\hat{\epsilon}, D)$ and the symmetric parameters (γ, Δ) is obtained from:

$$z_n^L = D(i, j, z) + \big(1 + \hat{\epsilon}(i, j, z)\big) z_n^U \tag{S28a}$$

$$\Delta(i, j, z) + \big(1 + \gamma(i, j, z)\big) z_n^U = -\Delta(i, j, z) + \big(1 - \gamma(i, j, z)\big) z_n^L \tag{S28b}$$

Equation (S28a) describes the physical mapping from the unloaded to the loaded scatterer position, whereas Eq. (S28b) imposes that the symmetric transformations of the unloaded and loaded signals meet at the same intermediate axial coordinate for all scatterers within Ω_z. Substituting Eq. (S28a) into Eq. (S28b) and solving for $D(i, j, z)$ and $\hat{\epsilon}(i, j, z)$ yields:

$$\hat{\epsilon}(i, j, z) := \frac{2\gamma(i, j, z)}{1 - \gamma(i, j, z)}, \qquad D(i, j, z) := \frac{2\Delta(i, j, z)}{1 - \gamma(i, j, z)}. \tag{S29}$$

This establishes the relation between the symmetric deformation parameters and the corresponding physical strain and bulk displacement.

The numerical implementation is therefore formulated in terms of the symmetric variables, and the algorithm proceeds by performing a greedy search over the trial pairs (γ, Δ), selecting the optimal pair $(\gamma^{opt}, \Delta^{opt})$ for which the transformed unloaded and loaded signals best match each other. However, minimizing the integrated error jointly over (γ, Δ) and lateral displacement indices (δ_i, δ_j) at every voxel would be computationally prohibitive. To reduce this cost, we assume that the lateral displacement field induced by the mechanical loading is *locally* continuous. Under this assumption, the optimal lateral displacement indices $(\delta_i^{opt}, \delta_j^{opt})$are not estimated directly on the full OCT C-scan, but first on a coarse three-dimensional grid obtained by subsampling the C-scan along the x, y, and axial directions. We denote the corresponding coarse-grid coordinates by (i', j', z'). Here, z is treated as a continuous axial variable for notational convenience, although in the numerical implementation the optimization is evaluated only at sampled axial positions; in particular, z' denotes a coarsely subsampled subset of those sampled z-positions. The resulting estimates $\delta_i^{opt}(i', j', z')$ and $\delta_j^{opt}(i', j', z')$ are then interpolated onto the full OCT C-scan grid (i, j, z), substantially accelerating the computation. The symmetric optimization problem on the coarse grid is then defined as:

$$\mathcal{E}_{lat}^{U,L}(i', j', z', \gamma, \Delta, \delta_{i'}, \delta_{j'}) := \sqrt{\frac{\displaystyle\int_{\Omega_{z'}} \left|\widetilde{A}^U(i', j', s; \gamma, \Delta) - \widetilde{A}^L(i' + \delta_{i'}, j' + \delta_{j'}, s; -\gamma, -\Delta)\right|^2 ds}{\displaystyle\int_{\Omega_{z'}} \left|\widetilde{A}^L(i' + \delta_{i'}, j' + \delta_{j'}, s; -\gamma, -\Delta)\right|^2 ds}}, \tag{S30a}$$

$$\Big[\gamma^{opt}(i', j', z'), \Delta^{opt}(i', j', z'), \delta_i^{opt}(i', j', z'), \delta_j^{opt}(i', j', z')\Big] := \underset{(\gamma, \Delta, \delta_{i'}, \delta_{j'})}{\operatorname{argmin}} \Big\{\mathcal{E}_{lat}^{U,L}(i', j', z', \gamma, \Delta, \delta_{i'}, \delta_{j'})\Big\}. \tag{S30b}$$

$\mathcal{E}_{lat}^{U,L}$ is the local error array containing the normalized residual $\mathcal{E}_2$ defined in the main manuscript, for each coarse-grid position (i', j', z'), each tested symmetric deformation pair (γ, Δ), and each tested lateral shift $(\delta_{i'}, \delta_{j'})$.
After this first step, the estimated coarse-grid lateral displacement maps $\delta_i^{opt}(i', j', z')$ and $\delta_j^{opt}(i', j', z')$ are interpolated over the full OCT C-scan grid, yielding $\delta_i^{opt}(i, j, z)$ and $\delta_j^{opt}(i, j, z)$. These interpolated lateral shifts are then used to perform a second displacement estimation over all voxels (i, j, z) of the C-scan:

$$[\gamma^{opt}(i, j, z), \Delta^{opt}(i, j, z)] := \underset{(\gamma, \Delta)}{\operatorname{argmin}} \Big\{\mathcal{E}_{lat}^{U,L}\left(i, j, z, \gamma, \Delta, \delta_i^{opt}(i, j, z), \delta_j^{opt}(i, j, z)\right)\Big\}, \tag{S31}$$

where only the interpolated lateral shifts are carried forward from the coarse-grid stage. In the implementation, this realignment is performed by constructing a laterally corrected version of the loaded C-scan and its derivatives from the interpolated lateral-shift maps before evaluating the final objective. Because neighbouring axial fitting windows overlap, this pre-registration can smooth transitions between adjacent integer lateral shifts along depth, improving robustness when the effective lateral displacement varies gradually. The final estimates of γ and Δ, however, are still obtained by minimizing Eq. (S31) on the full grid.

Although one could also interpolate the coarse-grid estimates of the symmetric deformation parameters, in practice we found it more accurate to re-estimate γ and Δ on the full grid, since the objective function is more sensitive to axial deformation.

The symmetrical solution in Eq. (S31) estimates how much A^U must be compressed and how much A^L must be stretched and laterally shifted for the two signals to match at an intermediate configuration in the neighborhood of the voxel $[i, j, z]$. It is worth noting that the assumption of local continuity may fail in voxels located near structural boundaries in the sample, such as the interface between cancerous and healthy tissue or the boundary of an inclusion in a phantom. In these regions, the accuracy of the lateral displacement estimation, and therefore of the retrieved strains, may be reduced. However, these boundary voxels typically represent only a small fraction of the sample, so this limitation does not prevent the generation of high-quality elastograms. Moreover, conventional OCE methods are also expected to perform poorly in such regions, since they rely on the assumption of locally uniform strain. Finally, both minimization steps in Eq. (S30) and Eq. (S31), corresponding to the coarse-grid and full-grid stages, respectively, are preceded by a local linear correction applied to each trial pair γ and Δ, which improves the robustness of the optimization. Details of the computation of the transformed C-scans $\widetilde{A}^U(i, j, z; \gamma, \Delta)$ and $\widetilde{A}^L(i, j, z; \gamma, \Delta)$, as well as of the linear correction step, are provided in Notes 6.4 and 6.5-6.6, respectively.

6.3 Multi-scale grid search for local strain and displacement estimation. The local optimization described above is not performed over a single wide trial grid, as this would require evaluating too many candidate deformation pairs $(\hat{\epsilon}', D')$ and would therefore be computationally expensive. Instead, the algorithm uses a three-level coarse-to-fine strategy, in which the same optimization is repeated over progressively smaller trial grids. This enables a broad initial exploration of the admissible parameter range, followed by two refinement steps around the best candidate identified at the previous stage (see Fig. S1). At each level, the search is carried out on a rectangular

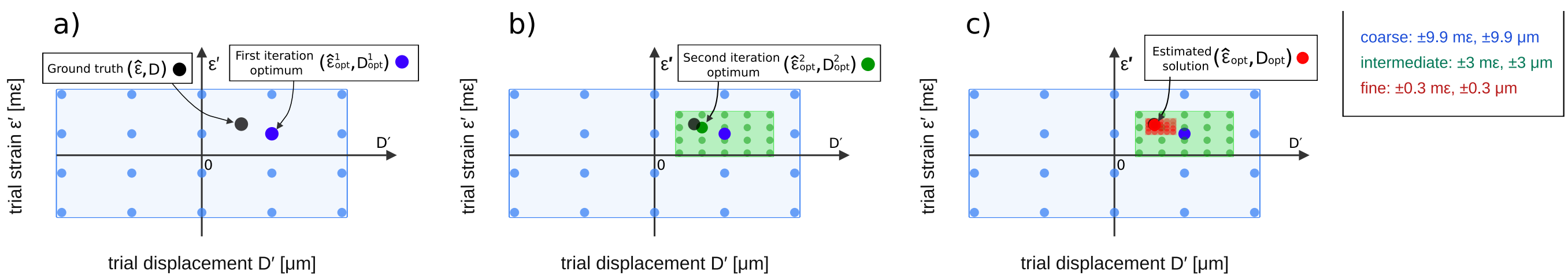

Figure S1: Schematic illustration of the coarse-to-fine grid search in the (ϵ', D') parameter space, corresponding to the trial strain and trial bulk displacement. Each search stage evaluates a discrete grid consisting of 13 trial displacement values and 11 trial strain values. (a) Coarse search over the full range, identifying the first coarse estimate $(\hat{\epsilon}^1_{\mathrm{opt}}, D^1_{\mathrm{opt}})$ (blue), shown relative to the ground-truth pair $(\hat{\epsilon}, D)$ (black). (b) Intermediate search performed on a smaller grid (green) centered at the first estimate (blue), yielding the second estimate $(\hat{\epsilon}^2_{\mathrm{opt}}, D^2_{\mathrm{opt}})$ (green). (c) Final fine search on the smallest grid (red) centered at the intermediate optimum, returning the final estimated solution $(\hat{\epsilon}_{\mathrm{opt}}, D_{\mathrm{opt}})$ (red). For clarity, this schematic does not include the linear refinement step discussed in Notes 6.5 and 6.6, which adds an additional small correction to each trial pair before selecting the optimum.

grid with 11 equally spaced trial values of $\hat{\epsilon}'$ and 13 equally spaced trial values of D', for a total of 11×13 evaluations per level. The finest grid spans $\hat{\epsilon}' \in [-0.3, 0.3]\,\mathrm{m}\epsilon$ and $D' \in [-300, 300]\,\mathrm{nm}$. This corresponds to a strain spacing of approximately $60\,\mu\epsilon$ and a displacement spacing of $50\,\mathrm{nm}$. As shown in Fig. S1a, the first step uses the coarsest grid, obtained by expanding the finest grid by a factor of 33 in both $\hat{\epsilon}'$ and D'. This corresponds to maximum tested magnitudes of $|\hat{\epsilon}'_{\mathrm{max}}| = 9.9\,\mathrm{m}\epsilon$ and $|D'_{\mathrm{max}}| = 9.9\,\mu\mathrm{m}$. The first iteration therefore explores the largest admissible region and identifies the coarse neighborhood in parameter space containing the optimal pair. A second search is then performed on an intermediate grid centered at the optimal pair $(\hat{\epsilon}^1_{\mathrm{opt}}, D^1_{\mathrm{opt}})$ found in the first step, as shown in Fig. S1b. This grid is obtained by expanding the finest grid by a factor of 10, corresponding to maximum tested magnitudes of $|\hat{\epsilon}'_{\mathrm{max}}| = 3\,\mathrm{m}\epsilon$ and $|D'_{\mathrm{max}}| = 3\,\mu\mathrm{m}$. Finally, as shown in Fig. S1c, a third search is performed on the finest grid, centered at the updated optimal pair $(\hat{\epsilon}^2_{\mathrm{opt}}, D^2_{\mathrm{opt}})$, yielding the final refined estimates of local strain and bulk displacement. In this way, the method combines broad initial exploration with progressively finer local refinement while keeping the computational cost low.

At each stage, the trial grid is defined in terms of the physical parameters $(\hat{\epsilon}', D')$, since these are the quantities of direct interest. For each tested pair, the corresponding symmetric parameters (γ, Δ) are computed through Eq. (S29) and used to construct the symmetrically transformed unloaded and loaded signals, on which the objective function is evaluated. The optimal symmetric pair identified at that stage is then converted back into the corresponding physical strain and bulk displacement, and this updated physical estimate is used to center the trial grid at the next refinement level.

6.4 Computation of the synthetically transformed OCT signals. For each trial transformation of $A^U(z)$, the coefficients of the additional Taylor expansion used by the algorithm are computed from (S9a) using the trial parameters γ and Δ:

$$c_0^U = 1, \tag{S32a}$$
$$c_1^U = i2k_0\,\gamma + 2W^2(1+\gamma)\big(\Delta + \gamma z\big), \tag{S32b}$$
$$c_2^U = -W^2\left(\gamma^2 + 2\gamma\right) + \frac{(c_1^U)^2}{2}, \tag{S32c}$$
$$c_3^U = -W^2\left(\gamma^2 + 2\gamma\right)c_1^U + \frac{(c_1^U)^3}{6}, \tag{S32d}$$
$$c_4^U = \frac{\left(-W^2\left(\gamma^2 + 2\gamma\right)\right)^2}{2} + \frac{\left(-W^2\left(\gamma^2 + 2\gamma\right)\right)(c_1^U)^2}{2} + \frac{(c_1^U)^4}{24}. \tag{S32e}$$

After that, we get $\alpha_n^U := e^{-i2k_0(\gamma z+\Delta)-W^2(\gamma z+\Delta)^2}c_n^U$ evaluating Eq. (S10). The coefficients α_n^L associated with the transformation of $A^L(z)$ are obtained analogously by evaluating (S10) with strain $-\gamma$ and bulk displacement $-\Delta$. The symmetrically-transformed unloaded and loaded signals are approximated by Eq. (S4) truncated at $n=4$, with:

$$\widetilde{A}^U(z;\gamma,\Delta) = \sum_{n=0}^{\infty}\alpha_n^U A_n^U(z) \approx \alpha_0^U A^U + \alpha_1^U A_1^U + \alpha_2^U A_2^U + \alpha_3^U A_3^U + \alpha_4^U A_4^U, \tag{S33a}$$
$$\widetilde{A}^L(z;-\gamma,-\Delta) = \sum_{n=0}^{\infty}\alpha_n^L A_n^L(z) \approx \alpha_0^L A^L + \alpha_1^L A_1^L + \alpha_2^L A_2^L + \alpha_3^L A_3^L + \alpha_4^L A_4^L, \tag{S33b}$$

where the generalized terms A_n^U and A_n^L are computed by using Eqs. (S24-S27) and do not depend on the trial deformation parameters.

6.5 Local linear correction of trial symmetric parameters. This section describes the local linear correction step used to refine all trial parameters γ and Δ in both optimization problems defined on the coarse grid, Eq. (S30), and on the final grid, Eq. (S31). The implementation performs the axial optimization independently for each sampled axial coordinate z. For a given trial pair (γ,Δ), once the symmetrically transformed signals $\widetilde{A}^U(z;\gamma,\Delta)$ and $\widetilde{A}^L(z;-\gamma,-\Delta)$ have been computed, the algorithm applies a local linearized correction to improve that trial pair. We stress that $\widetilde{A}^U(i,j,z;\gamma,\Delta)$ and $\widetilde{A}^L(i+\delta_i,j+\delta_j,z;-\gamma,-\Delta)$ are not the original unloaded and laterally shifted loaded C-scans, but their synthetically transformed versions obtained by applying the trial deformation (γ,Δ) to $A^U(i,j,z)$ and the opposite trial deformation $(-\gamma,-\Delta)$ to $A^L(i+\delta_i,j+\delta_j,z)$, respectively. In particular, $\widetilde{A}^U(i,j,z;0,0) = A^U(i,j,z)$ and $\widetilde{A}^L(i,j,z;0,0) = A^L(i,j,z)$ for all i, j, and z, since the trial pair $(0,0)$ corresponds to no synthetic deformation.

The optimization problems in Eqs. (S30) and (S31) imply that the optimal parameters $(\gamma^{opt},\Delta^{opt},\delta_i^{opt},\delta_j^{opt})$ are those that minimize the integrated residual between the two transformed signals, where the residual is defined as:

$$r(i,j,z;\gamma,\Delta,\delta_i,\delta_j) := \widetilde{A}^U(i,j,z;\gamma,\Delta) - \widetilde{A}^L(i+\delta_i,j+\delta_j,z;-\gamma,-\Delta). \tag{S34}$$

However, since r is evaluated only on a discrete set of trial values of γ, Δ, δ_i and δ_j, the true optimum is, in general, not expected to coincide exactly with one of the sampled grid points. For this reason, we introduced an additional step in the algorithm that locally refines each tested trial pair, so that every candidate can move closer to a nearby local minimum and, consequently, the overall discrete search can also approach the global minimum more accurately. In particular, for each trial pair (γ,Δ), a small linear correction (h_γ,h_Δ) is computed in order to perturb the current trial values and generate a refined candidate pair. From this point onward, to simplify the notation, we omit the explicit dependence on r and the related quantities on the lateral indices i,j and on the lateral displacement indices δ_i,δ_j applied to A^L. The linear correction is intended as a *small* local refinement, and can therefore be computed in closed form after linearizing the residual around the current trial pair:

$$r(z;\gamma+h_\gamma,\Delta+h_\Delta) \approx r(z;\gamma,\Delta) + h_\gamma\,\partial_\gamma r(z;\gamma,\Delta) + h_\Delta\,\partial_\Delta r(z;\gamma,\Delta), \tag{S35}$$

where $\partial_\gamma r$ and $\partial_\Delta r$ denote the derivatives of r with respect to γ and Δ, respectively. Details of the computation of these derivatives are provided in Note 6.6.

The correction (h_γ,h_Δ) is obtained by minimizing the integrated squared residual:

$$[h_\gamma,h_\Delta] = \underset{(u,v)\in\mathbb{R}^2}{\operatorname{argmin}}\int_{\Omega_z}\left|r(s) + \partial_\gamma r(s;\gamma,\Delta)u + \partial_\Delta r(s;\gamma,\Delta)v\right|^2 ds. \tag{S36}$$

By differentiating the integral in Eq. (S36) with respect to the variables u and v, and equating the two derivatives to zero, one obtains the normal equations for the minimum:

$$\begin{bmatrix}\langle\partial_\gamma r,\partial_\gamma r\rangle_z & \langle\partial_\gamma r,\partial_\Delta r\rangle_z\\ \langle\partial_\Delta r,\partial_\gamma r\rangle_z & \langle\partial_\Delta r,\partial_\Delta r\rangle_z\end{bmatrix}\begin{bmatrix}h_\gamma\\ h_\Delta\end{bmatrix} = \begin{bmatrix}-\langle\partial_\gamma r,r\rangle_z\\ -\langle\partial_\Delta r,r\rangle_z\end{bmatrix}, \tag{S37}$$

where

$$\langle X, Y\rangle_z := \int\limits_{\Omega_z} \mathrm{Re}\Big(\overline{X(s)}\,Y(s)\Big)\,ds. \tag{S38}$$

For each axial depth z and for each trial pair γ, Δ, Eq. (S37) is solved by inverting the corresponding (2×2) matrix. The resulting explicit expressions are:

$$h_\gamma = \frac{\langle \partial_\gamma r, \partial_\Delta r\rangle_z \langle \partial_\Delta r, r\rangle_z - \langle \partial_\Delta r, \partial_\Delta r\rangle_z \langle \partial_\gamma r, r\rangle_z}{\langle \partial_\gamma r, \partial_\gamma r\rangle_z \langle \partial_\Delta r, \partial_\Delta r\rangle_z - \langle \partial_\gamma r, \partial_\Delta r\rangle_z^2}, \tag{S39a}$$

$$h_\Delta = \frac{\langle \partial_\gamma r, \partial_\Delta r\rangle_z \langle \partial_\gamma r, r\rangle_z - \langle \partial_\gamma r, \partial_\gamma r\rangle_z \langle \partial_\Delta r, r\rangle_z}{\langle \partial_\gamma r, \partial_\gamma r\rangle_z \langle \partial_\Delta r, \partial_\Delta r\rangle_z - \langle \partial_\gamma r, \partial_\Delta r\rangle_z^2}. \tag{S39b}$$

For each depth z and for each related pair (h_γ, h_Δ), Eq. (S37) is solved *analytically*, which makes this optimization step computational advantageous and easily parallelizable. Once the optimal linear corrections h_γ and h_Δ have been computed, the algorithm *numerically* evaluates the corrected relative residual over the discrete set of trial pairs and selects the optimal one through a greedy search:

$$[\gamma^{opt}(z), \Delta^{opt}(z)] = \underset{(\gamma,\Delta)}{\text{argmin}} \left\{ \sqrt{\frac{\int\limits_{\Omega_z} \left| r(s;\gamma,\Delta) + h_\gamma \partial_\gamma r(s;\gamma,\Delta) + h_\Delta \partial_\Delta r(s;\gamma,\Delta)\right|^2 ds}{\int\limits_{\Omega_z} \left|\widetilde{A}^L(s;-\gamma,-\Delta)\right|^2 ds}} \right\}, \tag{S40}$$

where h_γ and h_Δ depend on $i, j, z, \gamma, \Delta, \delta_i, \delta_j$, but are treated as constant within the axial variable s within the range Ω_z, since they are estimated locally for that fitting range.

6.6 Computation of the derivatives $\partial_\gamma r$ and $\partial_\Delta r$. This section derives the expressions of the residual derivatives with respect to the symmetric deformation parameters γ and Δ, which are required for the local linear correction introduced in Note 6.5. Let $A(z)$ denote a generic carrier-removed OCT A-scan. The synthetic transformation associated with a trial strain γ and a trial bulk displacement Δ is defined by the same form as the loaded signal in Eq. (4b) of the main manuscript, after replacing $\hat{\epsilon}$ with γ and D with Δ, namely, $\widetilde{A}(z;\gamma,\Delta) := \sum_{j=1}^{N} \rho_j e^{-i2k_0\left(\Delta+(1+\gamma)z_j^U\right)} e^{-W^2\left(z-\left(\Delta+(1+\gamma)z_j^U\right)\right)^2}$. To simplify the derivation below, we introduce the following auxiliary quantities:

$$z_j^s := \Delta + (1+\gamma)z_j^U \tag{S41a}$$

$$\partial_\Delta z_j^s = 1 \tag{S41b}$$

$$\partial_\gamma z_j^s = z_j^U \tag{S41c}$$

$$z_j^U = \frac{z_j^s - \Delta}{1+\gamma} \tag{S41d}$$

$$-z_j^U = \frac{z - z_j^s - (z - \Delta)}{1+\gamma} \tag{S41e}$$

$$\widetilde{A}_n(z;\gamma,\Delta) = \sum_{j=1}^{N} \rho_j (z - z_j^s)^n e^{-i2k_0 z_j^s - W^2\left(z - z_j^s\right)^2} \tag{S41f}$$

where Eq. (S41d) is obtained by inverting Eq. (S41a), and Eq. (S41e) is derived from Eq. (S41a) after changing the sign; this latter form will be used below. It is important to distinguish the generalized terms $\widetilde{A}_n(z;\gamma,\Delta)$ from the quantities $A_n^U(z)$ and $A_n^L(z)$ introduced previously. Although they have the same formal structure, they are not the same objects: $\widetilde{A}_n(z;\gamma,\Delta)$ is associated with the synthetically deformed A-scan and depends on the trial scatterer positions z_j^s, whereas $A_n^U(z)$ and $A_n^L(z)$ are associated with the original unloaded and loaded signals and depend on the physical scatterer positions z_j^U and z_j^L, respectively.

In other words, $\widetilde{A}_n(z;\gamma,\Delta)$ represents the generalized A-scans of the synthetically deformed signal, while $A_n^U(z)$ and $A_n^L(z)$ are reconstructed directly from the measured spectra $I^U(\xi)$ and $I^L(\xi)$, as described in Note 6.1. In principle, computing the terms in Eq. (S41f) directly would require additional reconstruction steps beyond those described in Note 6.1. However, these terms are not evaluated in the implementation, since, under the approximation introduced below, their contribution becomes negligible. By applying Eq. (S41b) to

derivative of Eq. (S41f) with $n = 0$, we obtain the derivative of $\widetilde{A}(z;\gamma,\Delta)$ respect to Δ:

$$\begin{aligned}\partial_\Delta \widetilde{A}(z;\gamma,\Delta) &:= \partial_\Delta\left(\sum_{j=1}^{N}\rho_j e^{-i2k_0 z_j^s - W^2\left(z-z_j^s\right)^2}\right)\\ &= \sum_{j=1}^{N}\rho_j\left(-i2k_0 - 2W^2\left(z-z_j^s\right)(-1)\right)e^{-i2k_0 z_j^s - W^2\left(z-z_j^s\right)^2}\\ &= -i2k_0\sum_{j=1}^{N}\rho_j e^{-i2k_0 z_j^s - W^2\left(z-z_j^s\right)^2} + 2W^2\sum_{j=1}^{N}\rho_j\left(z-z_j^s\right)e^{-i2k_0 z_j^s - W^2\left(z-z_j^s\right)^2}\\ &= -i2k_0\widetilde{A}(z;\gamma,\Delta) + 2W^2\widetilde{A}_1(z;\gamma,\Delta) \end{aligned} \tag{S42}$$

Now we compute the derivative of $\widetilde{A}(z;\gamma,\Delta)$ with respect to γ, using Eqs. (S41c) and (S41e):

$$\begin{aligned}\partial_\gamma \widetilde{A}(z;\gamma,\Delta) &:= \partial_\gamma\left(\sum_{j=1}^{N}\rho_j e^{-i2k_0 z_j^s - W^2\left(z-z_j^s\right)^2}\right)\\ &= \sum_{j=1}^{N}\rho_j\left(-i2k_0 z_j^U - 2W^2\left(z-z_j^s\right)\left(-z_j^U\right)\right)e^{-i2k_0 z_j^s - W^2\left(z-z_j^s\right)^2}\\ &= \sum_{j=1}^{N}\rho_j(-z_j^U)\left(i2k_0 - 2W^2\left(z-z_j^s\right)\right)e^{-i2k_0 z_j^s - W^2\left(z-z_j^s\right)^2}\\ &= \sum_{j=1}^{N}\rho_j\left(\frac{z-z_j^s-(z-\Delta)}{1+\gamma}\right)\left(i2k_0 - 2W^2\left(z-z_j^s\right)\right)e^{-i2k_0 z_j^s - W^2\left(z-z_j^s\right)^2}\\ &= \sum_{j=1}^{N}\rho_j\left(-\frac{i2k_0(z-\Delta)}{1+\gamma} + \frac{i2k_0+2W^2(z-\Delta)}{1+\gamma}\left(z-z_j^s\right) - \frac{2W^2}{1+\gamma}\left(z-z_j^s\right)^2\right)e^{-i2k_0 z_j^s - W^2\left(z-z_j^s\right)^2}\\ &= -\frac{i2k_0(z-\Delta)}{1+\gamma}\widetilde{A}(z;\gamma,\Delta) + \frac{i2k_0+2W^2(z-\Delta)}{1+\gamma}\widetilde{A}_1(z;\gamma,\Delta) - \frac{2W^2}{1+\gamma}\widetilde{A}_2(z;\gamma,\Delta) \end{aligned} \tag{S43}$$

By assuming the parameter constraints discussed in Eqs. (S13), we obtain the following order-of-magnitude relations from Eqs. (S42) and (S43), based on the estimate in Eq. (S12):

$$\begin{aligned}\partial_\Delta \widetilde{A}(z;\gamma,\Delta) &:= -i2k_0\widetilde{A}(z;\gamma,\Delta) + 2W^2\widetilde{A}_1(z;\gamma,\Delta)\\ &\sim -i2k_0\widetilde{A}(z;\gamma,\Delta) + 2W\widetilde{A}(z;\gamma,\Delta)\\ &\sim -i2k_0\widetilde{A}(z;\gamma,\Delta)\left(1+i\frac{W}{k_0}\right)\\ &\sim -i2k_0\widetilde{A}(z;\gamma,\Delta) \end{aligned} \tag{S44a}$$

$$\begin{aligned}\partial_\gamma \widetilde{A}(z;\gamma,\Delta) &:= -\frac{i2k_0(z-\Delta)}{1+\gamma}\widetilde{A}(z;\gamma,\Delta) + \frac{i2k_0+2W^2(z-\Delta)}{1+\gamma}\widetilde{A}_1(z;\gamma,\Delta) - \frac{2W^2}{1+\gamma}\widetilde{A}_2(z;\gamma,\Delta)\\ &\sim -i2k_0 z\widetilde{A}(z;\gamma,\Delta) + (i2k_0+2W^2 z)\widetilde{A}_1(z;\gamma,\Delta) - 2W^2\widetilde{A}_2(z;\gamma,\Delta)\\ &\sim -i2k_0 z\widetilde{A}(z;\gamma,\Delta) + \left(\frac{i2k_0}{W}+2Wz\right)\widetilde{A}(z;\gamma,\Delta) - 2\widetilde{A}(z;\gamma,\Delta)\\ &\sim -i2k_0 z\widetilde{A}(z;\gamma,\Delta)\left(1-\frac{1}{Wz}+\frac{iW}{k_0}\right) \end{aligned} \tag{S44b}$$

$$\sim -i2k_0 z\widetilde{A}(z;\gamma,\Delta) \tag{S44c}$$

where we distinguish explicitly between the symbols $\sim$ and $\approx$. The symbol $\sim$ denotes an order-of-magnitude relation, and is used only to identify the dominant term within a sum of contributions. By contrast, the symbol $\approx$ denotes the resulting approximation obtained after neglecting the lower-order terms suggested by the preceding order-of-magnitude comparison. We stress that Eqs. (S44a) and (S44c) are not intended as uniformly accurate pointwise approximations along the whole A-scan. Rather, they are obtained by identifying the dominant terms in an order-of-magnitude sense and neglecting terms whose relative contribution is smaller on average over the fitting window Ω_z. In some cases, the neglected terms may still have an average magnitude of order 10% of the dominant term over Ω_z, and,

as already discussed below Eq. (S12), there may also exist isolated axial points at which such terms become locally much larger or even dominant. However, the quantities considered here enter the algorithm through squared integrals over Ω_z, so their relevance must be assessed mainly through their average behaviour over the fitting window, rather than through isolated pointwise deviations.

From Eq. (S44b) to Eq. (S44c), we neglected the term $\frac{1}{Wz}$. Although this term is not negligible for $|z| < \frac{1}{W} \sim 10\,\mu\mathrm{m}$, the approximation in Eq. (S44c) is intended to capture the dominant trend over the fitting window Ω_z. Even in the shallowest region, Ω_z contains several axial points for which this order-of-magnitude comparison remains valid. Therefore, Eqs. (S44a) and (S44c) indicate that the first terms in Eqs. (S42) and (S43) are dominant on average over Ω_z. Accordingly, the derivatives of the residual in Eq. (S34) are approximated by retaining only their dominant terms:

$$
\begin{aligned}
\partial_\Delta r(z;\gamma,\Delta) &= \partial_\Delta\Big(\widetilde{A}^U(z;\gamma,\Delta) - \widetilde{A}^L(z;-\gamma,-\Delta)\Big) \\
&= \partial_\Delta \widetilde{A}^U(z;\gamma,\Delta) - (-1)\partial_\Delta \widetilde{A}^L(z;-\gamma,-\Delta) \\
&\sim -i2k_0\widetilde{A}^U(z;\gamma,\Delta) - i2k_0\widetilde{A}^L(z;-\gamma,-\Delta) \qquad \text{(S45a)}\\
\partial_\gamma r(z;\gamma,\Delta) &= \partial_\gamma\Big(\widetilde{A}^U(z;\gamma,\Delta) - \widetilde{A}^L(z;-\gamma,-\Delta)\Big) \\
&= \partial_\gamma \widetilde{A}^U(z;\gamma,\Delta) - (-1)\partial_\gamma \widetilde{A}^L(z;-\gamma,-\Delta) \\
&\sim -\frac{i2k_0(z-\Delta)}{1+\gamma}\widetilde{A}^U(z;\gamma,\Delta) - \frac{i2k_0(z-(-\Delta))}{1+(-\gamma)}\widetilde{A}^L(z;-\gamma,-\Delta) \\
&= -\frac{i2k_0(z-\Delta)}{1+\gamma}\widetilde{A}^U(z;\gamma,\Delta) - \frac{i2k_0(z+\Delta)}{1-\gamma}\widetilde{A}^L(z;-\gamma,-\Delta) \qquad \text{(S45b)}
\end{aligned}
$$

These approximations are used in the current implementation of the method and were also verified numerically.

Supplementary Note 7. Gaussian spectral profile, width definition, and experimental spectral reshaping

7.1 Definition of the Gaussian spectral width

Throughout this work, the OCT A-scan model, the phase-difference analysis, and the corresponding numerical reconstructions are formulated using a Gaussian power spectral density. In this note, we clarify this Gaussian power spectral density assumption and define the width parameter W.

In what follows, the Fourier transform is expressed using the spatial-frequency variable ξ, which is Fourier-conjugate to z, rather than the wavenumber k. In the simulations, the Gaussian power spectral density is imposed directly in spatial-frequency space. Specifically, if ξ_0 denotes the central spatial frequency of the spectrum, we use a power spectral density of the form

$$
S_G(\xi) := \frac{1}{\sqrt{\pi}W} e^{-\left(\frac{2\pi(\xi-\xi_0)}{W}\right)^2}, \qquad \text{(S46)}
$$

where W is the corresponding Gaussian width parameter in angular spatial-frequency units, with inverse units of z, typically m^{-1}. To define W, we start from the wavelength interval used to represent the spectrum, denoted by $[\lambda_{\min}, \lambda_{\max}]$. This interval corresponds to the spatial-frequency interval $[\xi_{\min}, \xi_{\max}]$, where $\xi_{\min} = 1/\lambda_{\max}$ and $\xi_{\max} = 1/\lambda_{\min}$, so that its full width in spatial-frequency space is

$$
B = \xi_{\max} - \xi_{\min} = \frac{1}{\lambda_{\min}} - \frac{1}{\lambda_{\max}}. \qquad \text{(S47)}
$$

Since $S_G(\xi)$ is centred at ξ_0, it reaches its maximum over this interval at ξ_0. Moreover, because $\xi_0 = (\xi_{\min} + \xi_{\max})/2$, the interval edges $\xi_{\min}$ and $\xi_{\max}$ are equally distant from ξ_0, and therefore correspond to the same minimum value of $S_G(\xi)$ over $[\xi_{\min}, \xi_{\max}]$. We then choose W by prescribing the relative Gaussian level τ at the edges of this interval, namely

$$
\frac{\min\limits_{\xi\in[\xi_{\min},\xi_{\max}]} S_G(\xi)}{\max\limits_{\xi\in[\xi_{\min},\xi_{\max}]} S_G(\xi)} = \frac{S_G(\xi_{\min})}{S_G(\xi_0)} = \frac{S_G(\xi_{\max})}{S_G(\xi_0)} = \tau. \qquad \text{(S48)}
$$

From the Gaussian definition of $S_G(\xi)$ in Eq. (S46), the relative value at the edges of the interval is

$$\tau = \frac{S_G(\xi_{\min})}{S_G(\xi_0)} = \frac{S_G(\xi_{\max})}{S_G(\xi_0)} = e^{-\left(\frac{\pi B}{W}\right)^2}. \tag{S49}$$

Inverting this relation gives

$$W = \frac{\pi B}{\sqrt{\log(1/\tau)}}. \tag{S50}$$

Thus, B is fixed by the wavelength interval used to represent the spectrum, while τ specifies the relative Gaussian level imposed at the edges of this fixed interval.

7.2 Gaussian spectral reshaping of experimental data

For experimental data, the displacement-estimation algorithm described in the **Results** section of the main manuscript assumes that the measured spectra can be reshaped to a Gaussian profile before A-scan reconstruction. In particular, following the notation of Supplementary Note 6.2, we consider three-dimensional OCT acquisitions (C-scans), where each A-scan has lateral indices i and j along the x and y directions, respectively. A-scans and B-scans are recovered as special cases by using singleton dimensions along the missing lateral directions. Under this convention, let $I^U_{\mathrm{acq}}(i,j,\xi)$ and $I^L_{\mathrm{acq}}(i,j,\xi)$ denote the acquired raw unloaded and loaded C-scan spectra, respectively. We estimate the mean acquired power spectral density $S_{\mathrm{acq}}(\xi)$ by averaging $I^U_{\mathrm{acq}}(i,j,\xi)$ over the lateral indices i and j of the unloaded C-scan, and then define the reshaped Gaussian spectra used for reconstruction as:

$$I^U(i,j,\xi) = \frac{I^U_{\mathrm{acq}}(i,j,\xi)}{S_{\mathrm{acq}}(\xi)} S_G(\xi), \qquad I^L(i,j,\xi) = \frac{I^L_{\mathrm{acq}}(i,j,\xi)}{S_{\mathrm{acq}}(\xi)} S_G(\xi). \tag{S51}$$

The reshaping is therefore used mainly to make the experimental reconstructions more consistent with the Gaussian-spectrum formulation used in the theory. For the experimental data analysed in the main manuscript, we used $\tau \leq 10^{-2}$, which was chosen to represent the Gaussian power spectral density over an interval where its tails are negligible, rather than because the method requires this specific cutoff. We have verified heuristically that the methods and analyses in this work are robust when the acquired raw spectral data are preprocessed using Eq. (S51), and when higher values of τ are used.
