# Supplementary material for: Insight into phase-sensitive optical coherence tomography displacement measurement
Source: Nat Commun. 2026 Sep 16;17:10890. doi: 10.1038/s41467-026-77549-2 (PMC13677722; doi:10.1038/s41467-026-77549-2)
Supplement: Supplementary file 2 [file 41467_2026_77549_MOESM2_ESM.pdf]

nature portfolio

Peer Review File

Insight into Phase-Sensitive Optical Coherence Tomography Displacement Measurement

Corresponding Author: Mr Andrea Mazzolani

This file contains all reviewer reports in order by version, followed by all author rebuttals in order by version.

Version 0:

Reviewer comments:

Reviewer #2

(Remarks to the Author)
This manuscript presents a rigorous theoretical and experimental analysis of the limitations of the widely used phase difference equation in phase-sensitive OCT, with a focus on compression optical coherence elastography (OCE). The authors demonstrate that axial displacement is not strictly proportional to phase difference due to previously unaccounted non-linear terms, particularly in regions of dark speckle or sharp strain gradients. They identify two new intrinsic phase artifacts, oscillation-peaks and 2π-jumps, and introduce a novel displacement estimation method based on a series expansion that significantly improves accuracy and signal-to-noise ratio over conventional approaches. The work is potentially suitable for publication in Nature Communications, but only with important clarifications and enhancements in the following aspects:
1) The first sentence of the Abstract implies broad applicability across various phase-sensitive OCT modalities, including Doppler and dynamic OCT. However, the derivation, particularly Equation (3b), is specific to quasi-static compression OCE. In other modalities, where displacement profiles differ (e.g., flow-induced or vibrational motion), the non-linear phase effects described may not arise. The authors should clarify the scope of applicability or justify the generalization.
2) The coefficients 'alpha_n(z)' introduced in Equation (6) are central to the proposed model but are not defined in the main text. Including at least a qualitative or simplified description would improve clarity and make the derivation more self-contained for the reader.
3) Figure 2 validates Equation (6) under idealized, noise-free conditions. However, real OCT signals are affected by noise sources such as speckle decorrelation and shot noise. To assess the model's practical robustness, it would be helpful to show simulations with varying noise levels.
4) In Equation (7), the derivative A'(z) is not explicitly defined. Providing a definition, as the derivative of the complex OCT signal with respect to depth, would enhance clarity, particularly for readers less familiar with complex signal analysis.
5) The proposed method involves iterative fitting of elastic parameters 'epsilon' and 'D', which could be computationally intensive. Details on the algorithm's runtime performance and potential strategies for acceleration (e.g., GPU implementation or approximate models) would help assess its feasibility for real-time or high-throughput imaging scenarios.

(Remarks on code availability)

Reviewer #3

(Remarks to the Author)
Mazzolani et al. addressed the fundamental limitations of displacement estimation using phase-sensitive optical coherence tomography (OCT). A theoretical model based on individual scatterers is used to derive the phase change in the OCT signal resulting from sample displacement. The model reveals that estimation errors in displacement measurements are caused by low OCT signal amplitude due to speckle, rather than phase unwrapping errors. Both numerical simulations and experimental results support this insight. Finally, the authors propose a new algorithm to mitigate these errors and demonstrate that it outperforms conventional methods.

Overall, the manuscript is well-written, and the results effectively support the conclusions. I have several comments aimed at improving the descriptions and avoiding potential misrepresentations.

Comments

- Abstract, Line 5 and Discussion (Page 8, Left column, Line 35)

Phase-sensitive OCT is uncommon in the context of dynamic OCT. As noted in the review paper [Ref. 3], almost all dynamic OCT metrics are based on the intensity or amplitude of the OCT signal, not the phase. It is inappropriate to mention dynamic OCT as a primary application of phase-sensitive OCT without qualification. The authors should either remove the descriptions of dynamic OCT or cite specific dynamic OCT studies that rely on phase-sensitive measurements.

- Page 2, Left column, Line 21-25

"Dynamic OCT images the active motion of sub-cellular components, which are linked with the physiological state of cells. Such motion could also, to first approximation, be represented as a combination of bulk displacement and local strain."

I find it difficult to agree with this point. How can the random motion of sub-cellular components be accurately represented by bulk displacement and local strain? In a model combining bulk displacement and local strain, one would expect the moving directions of the scatterers to be aligned.

Sub-cellular motion typically involves displacements that are mutually uncorrelated. I do not believe this case can be accurately expressed by the authors' model in Eq. (3b), as describing such motions would require a stochastic displacement framework.

- Eq. (10) and Fig. 3

In Fig. 3 processed by the script "accurate_dphi.m", there are huge jumps in phase at depths of 292 um and 387 um, which cannot be reproduced with Eq. (10).
Please describe it in the manuscript and why these jumps cannot be expressed with Eq. (10).

- Unit of strain

Please provide more detail regarding the simulation parameters. For example, what does -3 m\epsilon represent? To ensure the paper is accessible to a broader readers, it would be helpful to explicitly define the meanings of "m" and "\epsilon" in this context, which are not common for readers not familiar with the strain measurements.

- Page 6, Left column, Line 13

What does "B" represent? It appears to be the product of the unloaded A-line and the complex conjugate of the loaded A-line (the Method); however, it is not defined at this stage. Please provide a definition of "B" here.

- Page 6, Left column, Line 20

Please clarify the definitions of "high oscillation peaks" and "low oscillation peaks." Without clear definitions, it is difficult for the reader to relate these descriptions to Fig. 6.

- Figs. 7 and 8

The legends for Figs. 7 and 8 differ from those in Fig. 6. Do they refer to different classes or categories? Please ensure consistency across figures.

- The proposed method, Eq. (11)

How does this optimization operate? The provided codes are very complicated and make it difficult to identify the algorithm for this minimization process.
Is this global optimization or local optimization? Are the parameters \epsilon and D bounded?
Please provide the details of the algorithm that was used.

- Page 7, left column, Line 8-32

Please provide the details of processing of the proposed method with experimental data.

- What level of truncation (n) was used for the experimental data?
- What was the total processing time?
- The authors stated that lateral averaging was not used. It means, axial averaging was performed?

- OCE signal simulations

An important simulation parameter is missing: the number of scatterers, N.
Please clarify this parameter and the method used to determine the scatterers' positions, z_j.

- OCE C-scans simulation (Page 9, Left column, Line 17)

The states: "Each OCE C-scan was filtered using a moving average of ... before performing strain retrieval."
Is this a preprocessing step for all strain retrieval methods?

The authors claim their new method does not use lateral averaging (Page 8, Line 19). Please clarify the exact processing procedures used for each strain retrieval method to avoid confusion.

- Supplementary Note 1, Line 11

Could the authors provide examples (e.g., n=0, 1, 2) to demonstrate that the coefficient c_n does not depend on z_j?

- Supplementary Note 2

The section is titled "Phase difference estimation in the case of bright speckle."

Is this derivation in this note valid only for bright speckle?
The authors appear to apply these equations to all cases, regardless of whether they involve bright or dark speckle.
Please correct the title if the derivation is intended to be universal.

- Numbering of equations

I recommend using a different labeling system for equations in the supplementary documents (e.g., Eq. S1) to distinguish them from the main text. This will help prevent confusion when referencing specific derivations.

- Eq. (8) in Supplementary Note 2

Please clarify the details of the approximation made between lines 6 and 7.

It is unclear why the term $\epsilon c_1 A_2^U$ disappeared in the derivation from line 7 to line 8.

- Eq. (9) in Supplementary Note 2

Please clarify the details of the approximation used to move from Eq. (9a) to Eq. (9b).

- Eq. (12) in Supplementary Note 3

In the final line, the first term is written as L_N (1 + <\epsilon>_N).
However, based on the definition of <\epsilon>_N and the preceding line, it appears it should be L_N <\epsilon>_N.
Please double-check this derivation.

- Supplementary Note 4

Supplementary Note 4 is currently not cited anywhere in the main text.

(Remarks on code availability)
- Missing script

The script "Non_linear_dphi.m" required for Fig. 1, which is described in the README file, is missing from the provided files.

- Commenting on scripts

Throughout the code, comments have not been added appropriately. In particular, the newly proposed strain estimation code (likely CSR_Tay_*.m) is difficult to understand.
Please add appropriate comments to enable interpretation of routine blocks and to explain hard-coded parameters.

Reviewer #4

(Remarks to the Author)
This manuscript presents an innovative approach for the phase-difference base displacement estimation in optical coherence elastography. The usually approach considers a linear relationship between phase and displacement. Here, a novel expansion for phase difference is derived, which contains higher order terms, in addition to the currently applied term. Moreover, two principal phenomena which perturb the usual assumed linear relationship between displacement and phase difference, are investigated.
This work seems original and represents promising advances for displacement estimation in OCE, which overcome the limitations of existing approaches.
Nevertheless, several descriptions in the manuscript lack sufficient discussion, affecting its clarity and reliability. Some comments and questions for the authors are listed below:
1) Figure 2, gives a clear illustrative example why it is important to consider the expansion in (6) with n greater than 0, and

the accuracy improves as n grows. So the claim that in OCE the choice n=1 generates a good estimation is not well justified. Wouldn't the choice n=4 improve the results?
2) Why is only the case n=1 explored along the manuscript?
3) The concepts of oscillation-peaks and 2π-jumps are illustrated throughout the paper, but they are not defined in a rigorous manner. A precise definition is therefore required.
4) Not enough detail is given about how to eveal the values of local strain and bulk displacement using equation (11).
After major revisions addressing the issues raised above, the manuscript could become suitable for publication.

(Remarks on code availability)
The results of the paper are reproducible.

Version 1:

Reviewer comments:

Reviewer #2

(Remarks to the Author)
The authors have carefully addressed my previous comments and substantially improved the manuscript. I have only the following minor comments:

1) The new perturbation analysis supports the possibility that dark-speckle phase instability may occur beyond compression OCE. However, it does not establish that the specific higher-order deformation model or proposed displacement-estimation algorithm applies generally to all phase-sensitive OCT modalities. Please distinguish the broader phase-instability mechanism from the compression-OCE-specific derivation and algorithm.

2) Please define all parameters in Eq. (3) clearly at their first occurrence. In particular, clarify the physical meaning, units, and sign convention of D. The definition of W as simply the "spectral width of the light source" may be imprecise. Please define W according to the assumed Gaussian spectrum and provide its exact relationship to the source bandwidth, including whether the bandwidth is expressed as a standard deviation or FWHM and whether it is defined in wavelength, wavenumber, or spatial-frequency space.

3) The additional noise and vibrational-motion simulations are helpful. However, the relative error reaches approximately 17.4% under the combined r=5% and D_v=10 nm condition, which may not warrant the description "accurate." Moreover, this analysis evaluates the truncated signal expansion rather than the complete strain-retrieval algorithm. Please moderate the robustness claim and clarify this distinction.

4) For reproducibility, please provide the hardware specifications and dataset dimensions associated with the reported processing times.

Subject to these minor clarifications, I consider my original concerns adequately addressed and support publication after minor revision.

(Remarks on code availability)

Reviewer #3

(Remarks to the Author)
The authors have thoroughly addressed the reviewers' concerns, and the manuscript has been substantially improved as a result.
I commend the authors on their thorough revisions and have no further comments.

(Remarks on code availability)
The code can reproduce the results of the paper. This is valuable to the community, as it enables reanalysis of the phase-related issues identified in the manuscript and the reuse of the proposed algorithm.
This will contribute to the development of algorithms that address issues in other types of phase-sensitive OCT.

Some revisions are still required.
Unnecessary lines are included in 'Figs7_8_WLS_VM_analysis.m' script, thus the script cannot run without modifications.
- undeclared, and perhaps not necessary, function 'get_info()'
- Multiple definitions of the 'saving_file' variable

Reviewer #4

(Remarks to the Author)
This manuscript presents an innovative approach for the phase-difference base displacement estimation in optical coherence elastography. The usually approach considers a linear relationship between phase and displacement. Here, a novel expansion for phase difference is derived, which contains higher order terms, in addition to the currently applied term. Moreover, two principal phenomena which perturb the usual assumed linear relationship between displacement and phase difference, are investigated. This work seems original and represents promising advances for displacement estimation in OCE, which overcome the limitations of existing approaches.
Several issues identified in the previous version of the manuscript have now been satisfactorily addressed. I have reviewed the authors' responses to the comments and appreciate their efforts in resolving all the my previous concerns.The article is of sufficient interest and quality and is recommended for publication in its current form.

(Remarks on code availability)
The results are reproducible.

Open Access This Peer Review File is licensed under a Creative Commons Attribution 4.0 International License, which permits use, sharing, adaptation, distribution and reproduction in any medium or format, as long as you give appropriate credit to the original author(s) and the source, provide a link to the Creative Commons license, and indicate if changes were made.
In cases where reviewers are anonymous, credit should be given to 'Anonymous Referee' and the source.
The images or other third party material in this Peer Review File are included in the article's Creative Commons license, unless indicated otherwise in a credit line to the material. If material is not included in the article's Creative Commons license and your intended use is not permitted by statutory regulation or exceeds the permitted use, you will need to obtain permission directly from the copyright holder.
To view a copy of this license, visit https://creativecommons.org/licenses/by/4.0/

Response to reviewers for manuscript: Insight into Phase-Sensitive Optical Coherence Tomography Displacement Measurement

On behalf of all authors I thank the reviewers for their carefully considered suggestions, comments and queries. We are grateful to have the opportunity to address them. We have modified the manuscript to make its purpose clearer and to address the specific queries raised by each reviewer.

In order to clearly denote changes made to the manuscript, added text is highlighted in the red-lined manuscript in yellow and deleted text is highlighted in red. The line numbers cited in this response refer to the line numbers shown in the red-lined manuscript and Supplementary Material. As highlighted text is typeset using LaTeX colour boxes, a single line number in the red-lined document may correspond to a block of text containing several visible lines of highlighted text. For example, "Line 34" corresponds to the highlighted revision blocks numbered 34 in the red-lined main manuscript, which actually contains 12 lines of text.

Response to all reviewers: high level modifications

- For clarity, both the red-lined manuscript and the red-lined Supplementary Information have been uploaded in the Nature Communications submission system under the file type **Supplementary Info**, together with the corresponding clean revised Supplementary Information.

- Given the reviewers' request for a more detailed description of the algorithm, we have now included the lateral-displacement search as part of the strain-retrieval procedure. This step had already been part of the broader implementation strategy, but it was not used to generate the figures in the original manuscript because we initially planned to present the lateral-displacement estimation procedure in a separate publication. For this reason, in the original inclusion-phantom analysis in the previous Fig. (8), now Fig. (9), we manually registered the data by applying a uniform one-pixel correction in the *x*-direction to the loaded C-scan before strain retrieval. This manual

correction was used as a pre-processing step, based on previous tests on the same dataset showing that the dominant lateral mismatch between the unloaded and loaded C-scans corresponded approximately to one voxel in the x-direction.

In the revised manuscript, however, we have removed this manual pre-processing step and now include the full procedure. Specifically, rather than applying a uniform shift to the whole loaded C-scan, the algorithm estimates the optimal lateral shift locally for each voxel, producing three-dimensional displacement maps in both the x- and y-directions. Applying this local lateral-displacement correction before strain retrieval improved the final strain predictions. We have now described this procedure explicitly in the section **Novel approach for displacement estimation** of the revised manuscript (see Lines 455-504), and added a complete step-by-step description of the implementation in the new Supplementary Note 6 (see Lines 75-100 of the Supplementary material).

As a result, the retrieved strains in the previous Fig. (8), now Fig. (9), improved slightly, with the difference being most visible in the line plots h) and i). In addition, to make the SNR comparison more meaningful, we revised the moving-mean and moving-SNR analyses shown in plots h) and i) of the same figure, respectively.

Specifically, instead of averaging within a one-dimensional 51-pixel, window along the x-direction, we now use a two-dimensional, 15×15 pixel window in the (x, y) plane, so that the averaging is performed over pixels that are, on average, closer to the pixel under evaluation. This yields a more localised SNR estimate and makes for a fairer comparison between the proposed and conventional strain retrieval methods, which confirms the superiority of the proposed method. We refer to the corresponding revision in the main text (see Lines 485-486):

> To illustrate this more clearly, in plots h) and i) of Fig. (8) show the moving averages and moving SNRs where each pixel is calculated by using a window of 51 pixels along the line $y = 550\mu$m of the elastograms shown in plots e), f) and g)

> To illustrate this more clearly, plots h) and i) of Fig. (9) show, respectively, the values along the line $y = 550\mu$m extracted from the moving-average and moving-SNR maps. In both cases, the underlying maps were computed using a 15×15 pixel moving window in the x- and y-directions on the en-face elastograms shown in panels e), f) and g).

- In Fig. (6) we have corrected the colour bar of the central panel, which shows the normalized B-scan magnitude. In the previous version, the displayed range was incorrectly reported as $\left[10^{-4}, 10^{-2}\right]$; this has now been corrected to $\left[10^{-3}, 10^{-2}\right]$, which is the range actually used in the figure (see central panel of Fig. (6)). We have also revised the caption and the y-axis labels of the top and central panels to better specify the normalization used in each case. In particular, the top panel now clarifies that the plotted A-scan magnitude is normalized by its own maximum, whereas the central panel clarifies that the displayed B-scan magnitude is normalized by the maximum magnitude of the unloaded B-scan.

- We additionally note that we updated the previous Fig. (7), now Fig. (8), to make the plotting procedure fully consistent with the manuscript description. The original plotting script generated the strain-dependent curves using only the positive-strain branch, although the manuscript describes the analysis in terms of the strain magnitude $|\hat{\epsilon}|$. Moreover, the representative fixed strain used in the other line plots was $\hat{\epsilon} = +1\, m\epsilon$, whereas the manuscript text refers to the representative case $\hat{\epsilon} = -1\, m\epsilon$. We therefore revised the plotting script to include all tested strain values through $|\hat{\epsilon}|$ and to use $\hat{\epsilon}_0 = -1\, m\epsilon$ as the representative fixed strain (see Lines 418-422).
During this check, we also clarified the representative values of the other non-

independent variables used in the line plots. In the previous manuscript text, these were reported as $D_0 = -0.2\mu$m and $z_0 = 180\mu$m. However, because the plotted curves are extracted from discretely sampled arrays, the actual values used in the script are the closest available sampled values, namely $D_0 = -0.375\mu$m and $z_0 = 200\mu$m. We have therefore updated the manuscript text to report the sampled values used in the plotted data (see Lines 418-419). The trends and conclusions remain the same, but the plotted curves and the numerical values discussed in the text have been updated for consistency.

Reviewer 2

This manuscript presents a rigorous theoretical and experimental analysis of the limitations of the widely used phase difference equation in phase-sensitive OCT, with a focus on compression optical coherence elastography (OCE). The authors demonstrate that axial displacement is not strictly proportional to phase difference due to previously unaccounted non-linear terms, particularly in regions of dark speckle or sharp strain gradients. They identify two new intrinsic phase artifacts, oscillation-peaks and 2π-jumps, and introduce a novel displacement estimation method based on a series expansion that significantly improves accuracy and signal-to-noise ratio over conventional approaches. The work is potentially suitable for publication in Nature Communications, but only with important clarifications and enhancements in the following aspects:

QUERY 2.1: *The first sentence of the Abstract implies broad applicability across various phase-sensitive OCT modalities, including Doppler and dynamic OCT. However, the derivation, particularly Equation (3b), is specific to quasi-static compression OCE. In other modalities, where displacement profiles differ (e.g., flow-induced or vibrational motion), the non-linear phase effects described may not arise. The authors should clarify the scope of applicability or justify the generalization.*
ANSWER 2.1

We thank the reviewer for this valuable comment, which we believe helped us strengthen the manuscript. We agree that the derivation based on Eq. (3b), as stated in the manuscript, is specific to quasi-static compression OCE. In the original version, we focused on the noise-free deterministic case to demonstrate that the reported phase effects are intrinsic to the OCT signal model, rather than being caused by experimental noise. However, we agree that this presentation may have made the broader relevance of the phase-instability mechanism unclear.

To address this point, we have added an additional perturbation analysis beyond the deterministic quasi-static compression model. The purpose of this analysis is not simply to test the effect of noise, but to show that the same phase-instability mechanism can arise whenever phase differences are computed from perturbed speckled complex OCT signals. Let $A^U(z)$ and $A^L(z)$ denote two *ideal* complex OCT signals used to compute a phase difference in any phase-sensitive OCT approach employing speckled complex OCT images, such as compression OCE, Doppler OCT, phase-sensitive dynamic OCT, or related modalities. In realistic measurements, however, both complex OCT signals may be affected by small perturbations, for example due to stochastic scatterer motion, vibration, additive noise, or other changes occurring between repeated acquisitions. What matters for the phase difference is the relative perturbation between the two signals, which we write here as a perturbation of the second signal with respect to the first. The measured phase difference can therefore be written as $\Delta\phi(z) = \phi^U(z) - \phi^L_{\text{pert}}(z) = \left[\phi^U(z) - \phi^L(z)\right] + \left[\phi^L(z) - \phi^L_{\text{pert}}(z)\right]$. The first term is the ideal phase difference associated with the modality-specific motion, such as

compression, flow, or vibration. The second term is the additional phase contribution that can arise in realistic measurements due to perturbation of the complex OCT signal itself. Therefore, even if the ideal phase difference $\phi^U(z) - \phi^L(z)$ were smooth and well described by a specific displacement model, the perturbation-induced term $\phi^L(z) - \phi^L_{\text{pert}}(z)$ can introduce additional non-linear phase behaviour.

To isolate this perturbation-induced contribution, we have added a dedicated section in which we analyse the phase difference generated only by a small perturbation of a complex OCT signal. In the revised manuscript, this is done by considering a perturbed version $A^L_{\text{pert}}(z)$ of an ideal signal $A^L(z)$, and by studying the corresponding perturbation-induced phase term $\Delta\phi^L_{\text{pert}}(z) = \phi^L(z) - \phi^L_{\text{pert}}(z)$. In the absence of perturbations, this term is identically zero. Therefore, any oscillation-peak or 2π-jump generated in this analysis cannot be attributed to the specific quasi-static compression profile assumed in Eq. (3b), but only to the perturbation of the speckled complex OCT signal. We show that even very small perturbations, including stochastic vibrational scatterer motion and/or additive Gaussian noise, are sufficient to generate both oscillation-peaks and 2π-jumps (see the new section **Generality of oscillation-peaks and 2π-jumps in speckled OCT signals**, Lines 279-283, and the new Fig. (5)).

These results demonstrate that the phase-instability mechanism identified in this work is not restricted to the specific compression-OCE deformation model, but can arise in any phase-sensitive OCT technique in which phase differences are computed from speckled OCT signals. As shown in the previous section and illustrated in Fig. (4), the key mechanism is that, near dark speckles, the complex OCT signal can pass close to the origin of the complex plane, where the phase is extremely sensitive to small perturbations. Therefore, Eq. (3b) should be understood as providing the analytical derivation for the compression-OCE case, rather than as a complete model for all phase-sensitive OCT modalities.

This clarification is also important for the scope of the proposed displacement-estimation framework. Since phase-instability events can arise from perturbations of speckled complex OCT signals, methods that rely directly on the PHDE may be sensitive to these effects whenever the phase difference is used as a displacement estimator. In the revised manuscript, we therefore also clarify the mathematical link between the PHDE and our signal model. In particular, we now show explicitly that the PHDE corresponds to the lowest-order, $n = 0$, truncation of Eq. (6) (see Line 182):

It is worth noting that the PHDE assumption is equivalent to approximating the loaded A-scan by the $n = 0$ truncation of Eq. (6), namely $A^L(z) \approx \alpha_0(z) A^U_0(z)$. In fact, under this assumption, we get $\Delta\phi(z) = \text{Arg}\left\{A^U(z)\overline{A^L(z)}\right\} \approx \text{Arg}\left\{A^U(z)\overline{\alpha_0(z)A^U_0(z)}\right\} = \text{Arg}\left\{\overline{\alpha_0(z)}\,|A^U(z)|^2\right\} = 4\pi\xi_0(D + \hat{\epsilon}z)$, since $|A^U(z)|^2$ is real and non-negative. Therefore, by dividing both sides by $2k_0$, we obtain Eq. (1).

In addition, as discussed in detail in ANSWER 2.3, the approximation of the loaded A-scan given by Eq. (6) becomes progressively more accurate as the truncation order n increases. This is relevant because the PHDE corresponds to the lowest-order case $n = 0$, whereas the new displacement-estimation method proposed in this work uses Eq. (6) truncated at $n = 4$. The proposed method therefore relies on a more accurate representation of the relationship between the unloaded and loaded complex OCT signals, making it substantially more robust than conventional displacement-estimation methods based on the PHDE.

QUERY 2.2: *The coefficients '$\alpha_n(z)$' introduced in Eq. (6) are central to the proposed model but are not defined in the main text. Including at least a qualitative or simplified description would improve clarity and make the derivation more self-contained for the reader.*

ANSWER 2.2

We thank the reviewer for this helpful comment. We agree that the coefficients $\alpha_n(z)$ should be described more explicitly in the main text, and that doing so helps the reader become more familiar with these terms. To make the derivation more self-contained, we have now added a short explanation clarifying that the $\alpha_n(z)$ are the deterministic coefficients arising from the Taylor expansion. To give the reader a more concrete idea of these terms, we have added a new Supplementary Note **1.1 Calculation of the terms $c_n(z)$ and $\alpha_n(z)$** for the general derivation and the higher-order coefficients (see Lines 16-17 of the supplementary material). We also report the explicit expressions of α_0 and α_1 in the main text (see Line 172):

The explicit expressions of the first two terms are $\alpha_0 = e^{i\psi - W^2 d_0}$ and $\alpha_1 = e^{i\psi - W^2 d_0}\left[i\,2k_0\hat{\epsilon} + 2W^2(1+\hat{\epsilon})(\hat{\epsilon}z + D)\right]$, where $\psi = -2k_0(D + \hat{\epsilon}z)$ and $d_0 = (\hat{\epsilon}z + D)^2$. Higher-order coefficients are not reported explicitly here, but can be derived using the procedure described in Supplementary Note 1.1.

QUERY 2.3: *Figure 2 validates Eq. (6) under idealized, noise-free conditions. However, real OCT signals are affected by noise sources such as speckle decorrelation and shot noise. To assess the model's practical robustness, it would be helpful to show simulations with varying noise levels.*

ANSWER 2.3

We thank the reviewer for this valuable comment, which we believe helped us considerably strengthen the manuscript. Following this suggestion, we have modified Fig. (2) to assess the robustness of Eq. (6) under non-ideal conditions. In particular, we now test the approximation after perturbing the ideal relationship between the unloaded and loaded A-scans in two controlled ways: by adding Gaussian noise to the complex OCT signals, and by applying small stochastic perturbations to the scatterer positions after compression.

We note that speckle decorrelation is not an additive noise source in the same sense as Gaussian signal noise. Rather, in this context it refers to a reduction in the similarity between the two reconstructed complex OCT signals acquired at different times. Eq. (6) itself is formulated at the level of the complex OCT signal and does not treat speckle as an additional noise term. In the ideal deterministic model, A^L is related to A^U by the scatterer displacement model, and Eq. (6) describes this relationship. In practice, however, the measured loaded signal can deviate from this ideal relationship because of noise, motion of scatterers, or other changes between acquisitions. The added simulations therefore test the robustness of the truncated approximation in Eq. (6) when this ideal relationship between A^U and A^L is perturbed.

The updated Fig. (2) now includes both the original noise-free comparison and additional error maps showing the robustness of Eq. (6) under these perturbations (see Fig. (2) and Lines 173-174):

Fig. (2) shows a plot of the real part of the estimation of the loaded A-scan, by truncating the sum in equation (6) at $n = 0, 1, 4$.

Plots a), b), c) and d) of Fig. (2) show, respectively, the real part of the unloaded A-scan and the approximations of the loaded A-scan obtained by truncating the sum in Eq. (6) at $n = 0$, $n = 1$, and $n = 4$, each compared with the actual loaded A-scan.

and Line (181):

To assess the robustness of Eq. (6) under more realistic conditions, we performed additional simulations in which the loaded A-scan was corrupted with additive complex Gaussian noise. Specifically, for each clean complex A-scan $A(z)$, we generated a noisy signal as $A_{\text{noise}}(z) = A(z) + r\,(n_R(z) + i\,n_I(z))\max_z |A(z)|$, where $n_R(z)$ and $n_I(z)$ are independent standard Gaussian random variables at each axial position z. With this convention, the real and imaginary parts of the complex Gaussian perturbation each have standard deviation $r\max_z |A(z)|$. Therefore, the root mean square amplitude of the complex perturbation is $\sqrt{2}\,r\max_z |A(z)|$. For example, a noise level $r = 0.05$ means that the real and imaginary components are each scaled by 5% of the maximum magnitude of the clean A-scan, corresponding to a complex root-mean-square perturbation amplitude of approximately 7.1% of $\max_z |A(z)|$. We also modeled vibrational scatterer motion by adding an independent Gaussian displacement to each scatterer after compression, so that the position of the jth scatterer became $\tilde{z}_j^L := z_j^L + \delta z_j^{vibr}$, where $\delta z_j^{vibr} \sim N(0, D_v^2)$, and D_v is the standard deviation of the vibrational displacement. Panels f), g), h), i) and j) of Fig. (2) show the integral relative error $\mathcal{E}_2$, defined as $\mathcal{E}_2(X,Y) := \sqrt{\frac{\int_{\mathbb{R}} |X(z)-Y(z)|^2 dz}{\int_{\mathbb{R}} |X(z)|^2 dz}}$, between the reference loaded A-scan and the approximation given by Eq. (6) when the series is truncated at $n = 0$, $n = 1, 2, 3$, and 4, respectively. In these maps, the level of additive Gaussian noise added to the loaded A-scans varies from 0 to 5%, while the vibrational displacement applied after loading varies from 0 to 10 nm. The black crosses in the top-left corners of panels f) and g) and the red cross in the panel j) indicate the noise-free cases and correspond to the A-scans shown in plots b), c) and d), with relative errors of 58%, 33% and 1.7%, respectively. Likewise, the black cross in the bottom-right corner of panel j) corresponds to plot e), where 5% additive complex Gaussian noise is added to the truncated A^L with $n = 4$, together with vibrational scatterer displacement of standard deviation $D_v = 10$ nm, with relative errors around 17.4%.

Overall, plot d), plot e) and the bottom panels show that the approximation in Eq. (6) improves as the truncation order n increases, and the case $n = 4$ remains accurate even in the presence of high levels of noise and vibrational perturbations.

QUERY 2.4: *In Equation (7), the derivative A'(z) is not explicitly defined. Providing a definition, as the derivative of the complex OCT signal with respect to depth, would enhance clarity, particularly for readers less familiar with complex signal analysis.*

ANSWER 2.4

In the revised manuscript, we have clarified that $A(z)$ is a complex-valued function of the real axial coordinate z, and that $A'(z)$ denotes its derivative with respect to depth. We have also specified that this derivative can be expressed in terms of the real and imaginary parts of the OCT signal (see Line 200):

$A'(z)$ denotes the derivative of the complex OCT signal with respect to the axial coordinate $z \in \mathbb{R}$, namely, $A'(z) := \frac{d}{dz} Re\{A(z)\} + i\frac{d}{dz} Im\{A(z)\}$.

QUERY 2.5: *The proposed method involves iterative fitting of elastic parameters 'epsilon' and 'D', which could be computationally intensive. Details on the algorithm's runtime performance and potential strategies for acceleration (e.g., GPU implementation or approximate models) would help assess its feasibility for real-time or high-throughput imaging scenarios.*

ANSWER 2.5

We thank the reviewer for this important comment. In the revised manuscript, we have added a new subsection in which we report the computational time required by the

proposed method to process the inclusion phantom data shown in Fig. (9), together with the corresponding times for WLS and VM. This subsection also briefly discusses the main computational bottleneck of the proposed method and possible directions for future acceleration. The title of this new subsection is (see Line 505):

Computational time and scope of the proposed method

In this section we show that the implementation of the new algorithm is currently considerably slower than WLS and VM, and discuss how we aim to improve computational time in future work.

We clarify that the aim of the proposed method is not to provide a real-time strain-retrieval algorithm, nor to compete at this stage with conventional OCE methods in terms of computational speed. Rather, its purpose is to show that, once the limitations of the PHDE are taken into account, substantially more robust strain estimation is possible.

Following the reviewer's suggestion, we also considerably expanded the section in the main manuscript titled (see Lines 455-504): **Novel approach for displacement estimation** and added a complete step-by-step description of the implementation in the new Supplementary Note 6 (see Lines 75-100 of the Supplementary material).

Reviewer 3

Mazzolani et al. addressed the fundamental limitations of displacement estimation using phase-sensitive optical coherence tomography (OCT). A theoretical model based on individual scatterers is used to derive the phase change in the OCT signal resulting from sample displacement. The model reveals that estimation errors in displacement measurements are caused by low OCT signal amplitude due to speckle, rather than phase unwrapping errors. Both numerical simulations and experimental results support this insight. Finally, the authors propose a new algorithm to mitigate these errors and demonstrate that it outperforms conventional methods. Overall, the manuscript is well-written, and the results effectively support the conclusions. I have several comments aimed at improving the descriptions and avoiding potential misrepresentations.

QUERY 3.1: *- Abstract, Line 5 and Discussion (Page 8, Left column, Line 35) Phase-sensitive OCT is uncommon in the context of dynamic OCT. As noted in the review paper [Ref. 3], almost all dynamic OCT metrics are based on the intensity or amplitude of the OCT signal, not the phase. It is inappropriate to mention dynamic OCT as a primary application of phase-sensitive OCT without qualification. The authors should either remove the descriptions of dynamic OCT or cite specific dynamic OCT studies that rely on phase-sensitive measurements.*

ANSWER 3.1:

We thank the reviewer for this important clarification. We agree that our original wording was too broad, since it could suggest that dynamic OCT is generally a primary phase-sensitive OCT application, we have therefore revised the manuscript.

In the Abstract, we have removed "dynamic OCT" from the main list of phase-sensitive OCT modalities (see Line 5), and modified the closing sentence to mention dynamic OCT as a possible application:

We believe our analysis will have a significant impact upon the the way displacement is estimated from phase-sensitive OCT data, beyond compression optical coherence elastography, and will therefore be important to the extensive range applications of phase-sensitive OCT.

We believe our analysis will have a significant impact upon the the way displacement is estimated from phase-sensitive OCT data beyond compression OCE, and may also be relevant to emerging phase-sensitive implementations of dynamic OCT in which phase changes are used to quantify motion.

In the Introduction, we now explicitly state that dynamic OCT is most commonly based on temporal fluctuations in the intensity or amplitude of the OCT signal. We also clarify that this differs from more recent dynamic OCT studies in which phase-based quantities have been investigated. To support this qualification, we have added two specific references: one describing a phase-sensitive dynamic OCT approach for quantifying intracellular motion from repeated complex OCT measurements [12], and one including phase variance among the dynamic OCT metrics evaluated for breast tumour margin assessment [13] (see Line 34):

Dynamic OCT has emerged recently as a powerful label-free approach for observing cellular and intracellular motion, most commonly by analysing temporal fluctuations in the intensity or amplitude of the OCT signal [11]. Although conventional dynamic OCT metrics are generally not based on phase-difference displacement estimation, recent studies have begun to investigate phase-based dynamic OCT analyses. For example, Schulz-Hildebrandt *et al.* introduced a phase-sensitive dynamic OCT approach to quantify intracellular motion from phase changes between repeated complex OCT measurements [12], while Bellesini *et al.* included phase variance among the methods used to visualise three-dimensional dynamic OCT contrast for breast tumour margin assessment [13].

and removed the old text (see Line 26):

Dynamic OCT has emerged recently as a powerful label-free approach for observing cellular and intracellular motion [3].

We have also revised the Discussion so that dynamic OCT is no longer grouped with Doppler OCT and OCE as a general phase-sensitive modality (see Line 509).

QUERY 3.2: *- Page 2, Left column, Line 21-25*
"Dynamic OCT images the active motion of sub-cellular components, which are linked with the physiological state of cells. Such motion could also, to first approximation, be represented as a combination of bulk displacement and local strain."
I find it difficult to agree with this point. How can the random motion of sub-cellular components be accurately represented by bulk displacement and local strain? In a model combining bulk displacement and local strain, one would expect the moving directions of the scatterers to be aligned.
Sub-cellular motion typically involves displacements that are mutually uncorrelated. I do not believe this case can be accurately expressed by the authors' model in Eq. (3b), as describing such motions would require a stochastic displacement framework.

ANSWER 3.2: We thank the reviewer for this important comment. We agree that the original statement was inaccurate. We have removed the statement that (see Line 46):

Dynamic OCT images the active motion of sub-cellular components, which are linked with the physiological state of cells. Such motion could also, to first approximation, be represented as a combination of bulk displacement and local strain.

To further address the reviewer's concern about non-deterministic scatterer motion, we have added an additional perturbation analysis which does not assume that the motion can be described by the deterministic compression model of Eq. (3b). The purpose of this analysis is to show that these phase-instability events are not limited to an ideal deterministic strain or bulk-displacement field. In experimental phase-sensitive OCT, the two complex signals used to compute a phase difference are almost inevitably affected by small relative perturbations, for example due to stochastic scatterer motion, vibration, noise, or other changes between acquisitions. We show that even very small perturbations of this kind can generate oscillation-peaks and 2π-jumps in speckled complex OCT signals, so these non-linear phase events should be regarded as a broadly relevant feature of realistic phase-sensitive OCT measurements rather than as a special case of the compression-OCE model. In the revised manuscript, we now consider an ideal complex OCT signal $A^L(z)$ and a perturbed version $A^L_{\text{pert}}(z)$, with phase $\phi^L_{\text{pert}}(z)$. The perturbation-induced phase contribution is then $\Delta\phi^L_{\text{pert}}(z) = \phi^L(z) - \phi^L_{\text{pert}}(z)$. This term is independent of the particular deterministic model used to generate the original OCT signal. We show that even very small perturbations of $A^L(z)$, including additive Gaussian noise or stochastic vibrational scatterer motion, can generate oscillation-peaks and 2π-jumps. This analysis is included in the new section entitled:

Generality of oscillation-peaks and 2π-jumps in speckled OCT signals (see Lines 278-282)

and in the new Fig. (5) (between Lines 279 and 283).

This added analysis does not claim to provide a complete stochastic displacement framework for dynamic OCT. Instead, it addresses the specific point raised by the reviewer by showing that the non-linear phase phenomena analysed in this work are not restricted to the ideal deterministic compression model. They can also arise

from stochastic perturbations of speckled complex OCT signals, because near dark speckles the local complex trajectory passes close to the origin of the complex plane, where the phase is highly sensitive to small perturbations. Thus, although Eq. (3b) remains the analytical model used for the compression-OCE derivation, the underlying phase-instability mechanism is expected to be relevant more broadly in phase-sensitive OCT modalities in which phase differences are computed from speckled complex signals.

QUERY 3.3: *- Eq. (10) and Fig. 3*
In Fig. 3 processed by the script "accurate-dphi.m", there are huge jumps in phase at depths of 292 um and 387 um, which cannot be reproduced with Eq. (10). Please describe it in the manuscript and why these jumps cannot be expressed with Eq. (10).
ANSWER 3.3:

We thank the reviewer for this important observation. We also appreciate the care and effort reflected in the detailed review, including this and the related queries. We agree that some phase jumps in Fig. (3) cannot be reproduced by Eq. (10). This is because Eq. (10) is a low-order analytical approximation designed to reproduce the leading non-linear oscillatory terms of $\Delta\phi$, but it is less robust in dark-speckle regions. In such regions, higher-order non-linear phase behaviour can dominate.

To clarify this point, we have revised Fig. (3) to explicitly show an example where Eq. (10) fails in dark speckle (see updated Fig. (3)). We have also modified the corresponding text in the manuscript (Lines 218-219):

The plot illustrates that the oscillations are faithfully reproduced by the approximate form.

Plot b) illustrates that the oscillations in $\Delta\phi$ are faithfully reproduced by the approximate form in Eq. (10) over regions containing both bright and dark speckles. The corresponding normalized magnitude of the unloaded A-scan is shown on a log scale in Plot a). However, Plots c) and d) also show that even this more accurate approximation is less robust in dark speckle, where higher-order non-linear phase behaviour may not be faithfully reproduced by Eq. (10). As we clarify below, in dark speckle the non-linear oscillations can reach magnitudes of approximately 2π (as shown in plot (d)).

These changes make clear that Eq. (10) captures the leading oscillatory deviations from the PHDE in bright speckle, but is not intended to reproduce all dark-speckle phase jumps, which are analysed more generally in the following section through the complex-plane interpretation of oscillation-peaks and 2π-jumps.

QUERY 3.4: *- Unit of strain: Please provide more detail regarding the simulation parameters. For example, what does -3 mϵ represent? To ensure the paper is accessible to a broader readers, it would be helpful to explicitly define the meanings of "m" and "ϵ" in this context, which are not common for readers not familiar with the strain measurements.*
ANSWER 3.4:
We agree that the notation ' ϵ ', which is commonly used in OCE and in elastography more broadly, may not be immediately clear. In the revised manuscript, we now clarify that although strains are dimensionless, we use the symbol ϵ only to make clear that the reported numerical values refer to strain. The prefix m denotes “milli”, so that $1\,m\epsilon = 10^{-3}$ strain. Therefore, for example, $-3\,m\epsilon$ corresponds to $\hat{\epsilon} = -3 \times 10^{-3}$, i.e. a compressive strain of -0.3% (see Line 58):

Although strain is dimensionless, for clarity throughout the manuscript we use the symbol ϵ as a strain marker, while $\hat{\epsilon}$ denotes the strain value used in the model; the hat is used only to distinguish the strain variable from the dimensionless unit ϵ. For example, $\hat{\epsilon} = -3\, m\epsilon$ corresponds to a compressive strain of -0.3‰, where m denotes the metric prefix milli.

This clarification distinguishes the notation ϵ, used only to indicate that a value refers to strain, from $\hat{\epsilon}$, which denotes the actual strain value used in the model.

QUERY 3.5: *- Page 6, Left column, Line 13*

What does "B" represent? It appears to be the product of the unloaded A-line and the complex conjugate of the loaded A-line (the Method); however, it is not defined at this stage. Please provide a definition of "B" here.

ANSWER 3.5:

We thank the reviewer for pointing this out. To improve clarity, we have now added the definition of B at its first occurrence in the Results section (Line 348):

From now on, we denote as $B := A^U \overline{A^L}$ the complex phase-difference signal.

QUERY 3.6: *- Page 6, Left column, Line 20*

Please clarify the definitions of "high oscillation peaks" and "low oscillation peaks." Without clear definitions, it is difficult for the reader to relate these descriptions to Fig. 6.

ANSWER 3.6

We thank the reviewer for this helpful comment. We agree that, in the original version of the manuscript, the terms "high oscillation peaks" and "low oscillation peaks" were not defined clearly, and appeared only in the Methods section, which may have made the original Fig. (6), which now is Fig. (7), difficult to interpret. In the new version, we define clearly these non-linear events also in the main text. We added two paragraphs in the main text. In particular, we now provide a formal definition of 2π-jumps and oscillation-peaks, and define the oscillation-peak magnitude M_I to make clearer the latter definition (see Line 277):

We now provide a formal definition of these non-linear phenomena in the general case, where $A^{(1)}(z)$ is a complex OCT A-scan and $A^{(2)}(z)$ is a perturbed version of $A^{(1)}(z)$. Such a perturbation may arise, for example, from strain, bulk motion, vibration, or noise. Let z_{ds} be a local minimum of $|A^{(1)}(z)|$, and let $\Omega_{z_{ds}}$ denote a local axial interval centred at z_{ds} over which the corresponding phase perturbation is analysed. Let $\Gamma_1 := \{A^{(1)}(z) : z \in \Omega_{z_{ds}}\}, \qquad \Gamma_2 := \{A^{(2)}(z) : z \in \Omega_{z_{ds}}\}$ be the associated local trajectories in the complex plane. In practice, $\Omega_{z_{ds}}$ is chosen as the local interval used to isolate the single phase perturbation associated with the dark-speckle region around z_{ds}. We define the perturbation as a 2π-jump if $0 \in \mathrm{conv}(\Gamma_1 \cup \Gamma_2)$, where $\mathrm{conv}(\cdot)$ denotes the convex hull. Otherwise, it is defined as an oscillation-peak. In the latter case, its magnitude is defined as $M_I := \max_{z \in \Omega_{z_{ds}}} |\Delta\phi_{\mathrm{osc}}(z)|$, where $\Delta\phi_{\mathrm{osc}}(z)$ denotes the phase difference after subtraction of the local linear trend predicted by the PHDE.

Additionally, we explicitly state the thresholds used in the original Figs. (6,7,8), now Figs. (7,8,9), for the definition of "high-peak" and "low-peak" classes (see Line 352):

For the present analysis, OCE signal intervals were divided into three classes: intervals containing at least one 2π-jump; intervals without 2π-jumps but with oscillation-peak magnitude $M_I > 0.65 \times 2\pi$, referred to as the "high peak" class, and intervals without 2π-jumps but with $M_I < 0.55 \times 2\pi$, referred to as the "low peaks" class. Intermediate cases were left unclassified.

QUERY 3.7: - *Figs. 7 and 8*
The legends for Figs. 7 and 8 differ from those in Fig. 6. Do they refer to different classes or categories? Please ensure consistency across figures.
ANSWER 3.7
We thank the reviewer for pointing this out. We have revised the legend of the previous Fig. 6 (now Fig. 7) to make it consistent with the legends and class definitions used in the previous Figs. 7 and 8, which are now numbered as Figs. 8 and 9, respectively.

QUERY 3.8: - *The proposed method, Eq. (11)*
How does this optimization operate? The provided codes are very complicated and make it difficult to identify the algorithm for this minimization process. Is this global optimization or local optimization? Are the parameters ϵ and D bounded? Please provide the details of the algorithm that was used.
ANSWER 3.8
We thank the reviewer for this helpful comment, which motivated us to improve the manuscript by providing full details of the algorithm. The optimization is performed independently for each axial fitting window. It is not implemented as an unconstrained local optimization. Instead, it is a deterministic bounded coarse-to-fine search over the strain and bulk-displacement parameters, combined with a local linear correction applied to each tested candidate. Therefore, the method uses a global optimization with respect to the finite bounded trial grid considered at each search level.

In the revised manuscript, we have added a new section titled (see Lines 455-504): **Novel approach for displacement estimation**, which now contains a clearer description of the algorithm in the main text. A full step-by-step derivation has also been added in **Supplementary Note 6** (see Lines 75-100 of the Supplementary Material), which includes a schematic figure showing the parameter constraints used in the coarse-to-fine search.

QUERY 3.9: - *Page 7, left column, Line 8-32*
Please provide the details of processing of the proposed method with experimental data.

3.9a *What level of truncation (n) was used for the experimental data?*
The level of truncation used for the new displacement algorithm was $n = 4$, as it is now discussed in the main manuscript in the revised section **Novel approach for displacement estimation** (see Lines 455-504), and the new **Supplementary Note 6** (see Line 76 and Eq. (S33) of the Supplementary Material).

3.9b *What was the total processing time?*
ANSWER 3.9b:
In the revised manuscript, we have added a new subsection in which we report the computational time required by the proposed method to process the inclusion phantom shown in Fig. (9), together with the corresponding times for processing the same dataset with WLS (phase unwrapping included) and VM. This subsection also briefly discusses the main computational bottleneck of the proposed method and possible directions for future acceleration. The title of this new subsection is (Line 505):
Computational time and scope of the proposed method

In this section we show that the implementation of the new algorithm is considerably slower than WLS and VM, and discuss how we aim to improve computational time in future work.

We clarify that the aim of the proposed method in this article is not to provide a real-time strain-retrieval algorithm, nor to compete at this stage with conventional OCE methods in terms of computational speed. Rather, its purpose is to show that, once the limitations of the PHDE are taken into account, substantially more robust strain estimation is possible.

3.9c *The authors stated that lateral averaging was not used. It means, axial averaging was performed?*

ANSWER 3.9c:

The proposed method does not use either lateral or axial averaging to retrieve strain. In contrast, for WLS and VM, a $2 \times 2 \times 2$-voxel moving-average filter was applied to each OCE C-scan before strain retrieval to improve the robustness and accuracy of the estimates. We have clarified this in the revised manuscript (see Lines 490-491):

It is worth to mention that the proposed method does not use lateral averaging to retrieve the strain, which likely means that the model will have higher spatial resolution compared to VM an WLS methods.

It is worth mentioning that the proposed method does not use either lateral or axial averaging to retrieve strain. This may allow it to preserve higher spatial resolution than VM and WLS, for which a $2 \times 2 \times 2$-voxel moving-average filter was applied to each OCE C-scan before strain retrieval.

QUERY 3.10: *- OCE signal simulations*

An important simulation parameter is missing: the number of scatterers, N. Please clarify this parameter and the method used to determine the scatterers' positions, z_j.

ANSWER 3.10

To provide a complete description of the parameters used in the simulations, we have also added a new subsection entitled **General parameter setting** in the Methods section. This includes the number of scatterers N, together with the method used to determine the scatterer positions z_j^U (see Lines 548-549):

General parameter setting.

Throughout all simulations reported in this article, we used realistic OCT parameters. In particular, we assumed a central wavelength of 1278 nm and a spectral width of 237 nm. This corresponds to a central wavenumber of $k_0 \approx 4.9 \times 10^6$ m^{-1}. The parameter W was chosen so that the Gaussian spectrum centered at k_0 was approximately three orders of magnitude smaller at the spectral edges, yielding $W \approx 1.7 \times 10^5$ m^{-1}.

The scatterers z_j^U were placed randomly along the z-axis according to a uniform distribution, with a minimum density corresponding to 11 scatterers per coherence length of the OCT signal. For each scatterer, the scattering cross-section parameter ρ_j was also drawn from a uniform distribution on $(0, 1)$. This choice is consistent with the analysis of Hillman *et al.* [27], where OCT speckle formation is related to the effective number of scatterers contributing within the probed region, with strong deviations from fully developed speckle occurring when this number is below about five. In our one-dimensional OCT model, this corresponds to considering the number of randomly distributed scatterers within one coherence-gated axial interval. We numerically verified that approximately the same lower bound is sufficient to generate realistic OCT speckle signals. Therefore, by using 11 scatterers per coherence-gated axial interval as the minimum value, we ensure that the simulated signals are generated in a well-developed speckle regime.

QUERY 3.11: *- OCE C-scans simulation (Page 9, Left column, Line 17)*

The states: "Each OCE C-scan was filtered using a moving average of ... before per-

forming strain retrieval." Is this a preprocessing step for all strain retrieval methods? The authors claim their new method does not use lateral averaging (Page 8, Line 19). Please clarify the exact processing procedures used for each strain retrieval method to avoid confusion.

ANSWER 3.11

We thank the reviewer for pointing out this potential ambiguity. As clarified in ANSWER 3.9c, the proposed method does not use either lateral or axial averaging to retrieve strain. In contrast, a $2 \times 2 \times 2$-voxel moving-average filter was applied to each OCE C-scan generated with VM and WLS methods. To avoid confusion, we have also clarified the processing procedure in the **Methods** section (see Lines 607-608):

Each OCE C-scan was filtered using a moving average of $(2 \times 2 \times 2)$ voxels before performing strain retrieval.

Each OCE C-scan was filtered using a $(2 \times 2 \times 2)$ voxel moving average before strain retrieval with WLS and VM, as is commonly done with these methods to improve the robustness and accuracy of the estimates. In contrast, no averaging was applied before strain retrieval with the proposed displacement-estimation method.

QUERY 3.12: *- Supplementary Note 1, Line 11*

Could the authors provide examples (e.g., n=0, 1, 2) to demonstrate that the coefficient c_n does not depend on z_j?

ANSWER 3.12:

We thank the reviewer for this helpful suggestion. The key point is that the coefficients $c_n(z)$ are obtained by collecting the powers of $(z - z_j^U)^n$ in the Taylor expansion, and are therefore independent of the scatterer position z_j^U by construction. To make this clearer, we have added a new subsection in Supplementary Note 1.1, where we derive an explicit formula for all $c_n(z)$ and $\alpha_n(z)$ (see Lines 16-17 of the supplementary material): **1.1 Calculation of the terms $c_n(z)$ and $\alpha_n(z)$**

In this section, we show that $c_n(z)$, and therefore also $\alpha_n(z)$, depend on the OCT system parameters and on the deformation parameters, but not on the scatterer positions z_j^U (see Eq. (S9b)).

Additionally, in the new Supplementary Note 6.4 (see Line 91 of the Supplementary Material):

6.4 Computation of the synthetically transformed OCT signals, we now provide the explicit expressions of c_0, c_1, c_2, c_3, and c_4, which are the terms used in the fourth-order implementation of the proposed method (see Eq. (S32) in the revised Supplementary Material). Related to this clarification, in the main manuscript we have also added the explicit expressions of α_0 and α_1 (see Lines 172):

The explicit expressions of the first two terms are $\alpha_0 = e^{i\psi - W^2 d_0}$ and $\alpha_1 = e^{i\psi - W^2 d_0}\left[i\,2k_0\hat{\epsilon} + 2W^2(1+\hat{\epsilon})(\hat{\epsilon}z + D)\right]$, where $\psi = -2k_0(D + \hat{\epsilon}z)$ and $d_0 = (\hat{\epsilon}z + D)^2$. Higher-order coefficients are not reported explicitly here, but can be derived using the procedure described in Supplementary Note 1.1.

QUERY 3.13: *- Supplementary Note 2*

The section is titled "Phase difference estimation in the case of bright speckle." Is this derivation in this note valid only for bright speckle? The authors appear to apply these equations to all cases, regardless of whether they involve bright or dark speckle. Please correct the title if the derivation is intended to be universal.

ANSWER 3.13:

We agree with the reviewer that the scope of this derivation needed to be made clearer. The approximation derived in Supplementary Note 2 is based on assumptions that are more robust in bright speckle, and it is therefore more prone to error in

dark-speckle regions. In some dark-speckle regions it can still reproduce the observed phase behaviour well, whereas in others higher-order terms are non-negligible and the low-order approximation breaks down. These points correspond to dark-speckle regions that are later classified in the manuscript as high-oscillation peaks or 2π-jumps. To clarify this point, we have changed the title of the corresponding section (see Lines 187-188):

Phase difference approximation

Phase difference approximation in bright speckles

Additionally, we have updated the introduction of the same section as (see Lines 189-190)

In this section, we derive an approximation of $\Delta\phi$ based on equation (6) truncated at $n = 1$.

In this section, we derive an approximation of $\Delta\phi$ valid in bright speckles by using the definition of $\phi'(z)$ in Eq. (7). Both the numerator and denominator of the resulting ratio are expressed independently through the series representation in Eq. (6), but with different truncation orders: $n = 1$ in the numerator and $n = 0$ in the denominator. This low-order truncation was chosen because it is sufficient to reproduce the leading non-linear terms of $\Delta\phi$ beyond the PHDE while keeping the derivation analytically tractable (see Supplementary Note 2 for further details). Higher-order terms would improve the accuracy of the approximation, but they lead to substantially more complicated phase expressions and were not required to demonstrate, theoretically, the existence of these non-linear terms. Nevertheless, higher truncation orders of Eq. (6) are considered later in the manuscript.

We have also updated Fig. (3) to show both cases: a region where the approximation reproduces the phase oscillations well, and a dark-speckle region where it becomes less accurate. In the main text, we have updated the description of Fig. (3) as (see Lines 218-219):

The plot illustrates that the oscillations are faithfully reproduced by the approximate form.

Plot b) illustrates that the oscillations in $\Delta\phi$ are faithfully reproduced by the approximate form in Eq. (10) over regions containing both bright and dark speckles. The corresponding normalized magnitude of the unloaded A-scan is shown on a log scale in Plot a). However, Plots c) and d) also show that even this more accurate approximation is less robust in dark speckle, where higher-order non-linear phase behaviour may not be faithfully reproduced by Eq. (10). As we clarify below, in dark speckle the non-linear oscillations can reach magnitudes of approximately 2π (as shown in plot (d)).

QUERY 3.14: *- Numbering of equations*
I recommend using a different labeling system for equations in the supplementary documents (e.g., Eq. S1) to distinguish them from the main text. This will help prevent confusion when referencing specific derivations.
ANSWER 3.14
We agree with the reviewer. In the revised Supplementary material, all supplementary equations and the new figure have been renumbered using the prefix ' S ', so that they are now labelled as Eq. (S1), Eq. (S2), Fig. (S1), etc.

QUERY 3.15: *Eq. (8) in Supplementary Note 2*
Please clarify the details of the approximation made between lines 6 and 7.

It is unclear why the term $\epsilon c_1 A_2^U$ disappeared in the derivation from line 7 to line 8.

ANSWER 3.15

We agree that this approximation required further clarification. We note that lines 7 and 8 of Eq. (8) in the previous submission correspond to Eq. (S17a) and Eq. (S17b) in the revised supplementary material, respectively. In the revised version, we have expanded the explanation of this step and adjusted the derivation accordingly (see Line 34 of the Supplementary Material):

> Eq. (S17a) is obtained by approximating the numerator and denominator of the preceding ratio separately. Specifically, Eq. (S4) is truncated at order $n = 1$ in the numerator and at order $n = 0$ in the denominator. These relatively severe truncations were adopted in order to obtain a closed-form expression, which could not be derived for more accurate truncation orders. An additional approximation is introduced in Eq. (S17b), where the factor $(1 + \hat{\epsilon})$ is approximated as 1, since this term does not significantly affect the approximation. However, as explained in the section **Mapping between loaded and unloaded A-scans** of the main manuscript, this truncation choice is still more accurate than the PHDE, which corresponds to the $n = 0$ truncation in both the numerator and denominator. For this reason, the approximation still has sufficient fidelity to reproduce fundamental non-linear oscillations of $\Delta\phi$ that deviate from the PHDE, as is shown in Figure (3) of the main manuscript.

The statement that the PHDE assumption is equivalent to the $n = 0$ truncation of Eq. (6) has also been clarified in the main manuscript (see Line 182):

> It is worth noting that the PHDE assumption is equivalent to approximating the loaded A-scan by the $n = 0$ truncation of Eq. (6), namely $A^L(z) \approx \alpha_0(z) A_0^U(z)$. In fact, under this assumption, we get $\Delta\phi(z) = \text{Arg}\left\{A^U(z)\overline{A^L(z)}\right\} \approx \text{Arg}\left\{A^U(z)\overline{\alpha_0(z)A_0^U(z)}\right\} = \text{Arg}\left\{\overline{\alpha_0(z)}\,|A^U(z)|^2\right\} = 4\pi\xi_0(D + \hat{\epsilon}z)$, since $|A^U(z)|^2$ is real and non-negative. Therefore, by dividing both sides by $2k_0$, we obtain Eq. (1).

QUERY 3.16: *- Eq. (9) in Supplementary Note 2*

Please clarify the details of the approximation used to move from Eq. (9a) to Eq. (9b).

ANSWER 3.16

We note that Eq. (9a) and Eq. (9b) in the previous submission correspond to Eq. (S18a) and Eq. (S18b) in the revised supplementary material, respectively. We have revised the main manuscript where we added details about that approximation (see Line 37 of the Supplementary Material):

> The last term of Eq. (S18b) was neglected heuristically. Indeed, since $\text{Im}\left\{\frac{[A^U(z)]'}{A^U(z)}\right\} = \phi_U'(z)$, the term $(D + \hat{\epsilon}z)\phi_U'(z)$ depends on the instantaneous value of $\phi_U'(z)$, and therefore retains its sharp local oscillations. Because $\phi_U'(z)$ undergoes rapid positive and negative fluctuations, substantial cancellations occur in its integral, making $\phi_U(z)$ much smoother and typically smaller than the local term $(D + \hat{\epsilon}z)\phi_U'(z)$. In addition, the neglected term is multiplied by the small prefactor $-(\hat{\epsilon} + \hat{\epsilon}^2)$. For this reason, it acts mainly as a weak smooth correction and was neglected, while the term $(D + \hat{\epsilon}z)\phi_U'(z)$ was retained since it is responsible for the oscillatory behaviour of interest. This behavior was also verified numerically in all the simulations considered here.

QUERY 3.17: *- Eq. (12) in Supplementary Note 3*

In the final line, the first term is written as $L_N(1+ < \epsilon >_N)$. However, based on the definition of $< \epsilon >_N$ and the preceding line, it appears it should be $L_N < \epsilon >_N$. Please double-check this derivation.

ANSWER 3.17

We note that Eq. (12) in the previous submission corresponds to Eq. (S21) in the revised Supplementary Material. We thank the reviewer for identifying this inconsistency, which allowed us to correct an error in the derivation. In particular, the recursive expression for the loaded position was written incorrectly. We had written $s(L_j) = s(L_{j-1}) + (L_j - L_{j-1})\hat{\epsilon}_{j-1}$, whereas the correct expression is $s(L_j) = s(L_{j-1}) + (L_j - L_{j-1})(1 + \hat{\epsilon}_{j-1})$. This correction propagates through the derivation and leads to the reviewer's suggested form, $L_N \langle \hat{\epsilon} \rangle_N$, rather than $L_N(1 + \langle \hat{\epsilon} \rangle_N)$. The corrected derivation is now reported in the revised supplementary material (see Lines 51-52 of the Supplementary Material):

$$
\begin{aligned}
D_N &= s(L_N) - L_N(1 + \hat{\epsilon}_N) \\
&= s(L_{N-1}) + (L_N - L_{N-1})\,\hat{\epsilon}_{N-1} - L_N(1 + \hat{\epsilon}_N) \\
&= s(L_{N-2}) + (L_N - L_{N-1})\,\hat{\epsilon}_{N-1} + (L_{N-1} - L_{N-2})\,\hat{\epsilon}_{N-2} - L_N(1 + \hat{\epsilon}_N) \\
&\dots \\
&= \sum_{p=0}^{N-1} \left(L_{p+1} - L_p\right) \hat{\epsilon}_p - L_N(1 + \hat{\epsilon}_N) \\
&= L_N(1 + < \hat{\epsilon} >_N) - L_N(1 + \hat{\epsilon}_N) \qquad (12)
\end{aligned}
$$

The two terms subtracted in Eq. (12) are the real displacement $\left(L_N(1 + < \hat{\epsilon} >_N)\right)$ and the displacement that there would be in case of uniform strain $\hat{\epsilon}_N$ across the entire sample $\left(L_N(1 + \hat{\epsilon}_N)\right)$.

$$
\begin{aligned}
D_N &= s(L_N) - L_N(1 + \hat{\epsilon}_N) \\
&= s(L_{N-1}) + (L_N - L_{N-1})\,(1 + \hat{\epsilon}_{N-1}) - L_N(1 + \hat{\epsilon}_N) \\
&= s(L_{N-2}) + (L_N - L_{N-1})\,(1 + \hat{\epsilon}_{N-1}) + (L_{N-1} - L_{N-2})\,(1 + \hat{\epsilon}_{N-2}) - L_N(1 + \hat{\epsilon}_N) \\
&\dots \\
&= \sum_{p=0}^{N-1} \left(L_{p+1} - L_p\right) \left(1 + \hat{\epsilon}_p\right) - L_N(1 + \hat{\epsilon}_N) \\
&= L_N + \sum_{p=0}^{N-1} \left(L_{p+1} - L_p\right) \hat{\epsilon}_p - L_N - L_N \hat{\epsilon}_N \\
&= L_N < \hat{\epsilon} >_N - L_N \hat{\epsilon}_N
\end{aligned}
$$

The two terms subtracted in Eq. (S21) are the real displacement $\left(L_N < \hat{\epsilon} >_N\right)$ and the displacement that there would be in case of uniform strain $\hat{\epsilon}_N$ across the entire sample $\left(L_N \hat{\epsilon}_N\right)$.

QUERY 3.18: - *Supplementary Note 4*
Supplementary Note 4 is currently not cited anywhere in the main text.
ANSWER 3.18
This note was originally included as an additional clarification for the reader, rather than as a central part of the main derivation.

However, we agree that an explicit reference improves the clarity of the manuscript.

In particular, Supplementary Note 4 helps clarify that the behaviour of VM and WLS is determined by the magnitudes of the strain $\hat{\epsilon}$ and bulk displacement D, *and*, also by the resulting local displacement $D + \hat{\epsilon}z$. In particular, even when $|\hat{\epsilon}|$ and $|D|$ are individually large, VM and WLS can still perform well if $|D + \hat{\epsilon}z|$ remains small.

We have therefore added a reference to supplementary Note 4 in the main manuscript, where we discuss Figs. (8d) and (8h), and we show that the term $|D + \hat{\epsilon}z|$ has as significant impact on the accuracy of both VM and WLS. The added text reads (see Line 425):

It is worth noting that Fig. (8d) and Fig. (8h) show that even when $|\hat{\epsilon}|$ and $|D|$ are individually large, VM and WLS can still perform well if $|D + \hat{\epsilon}z|$ remains small, since $D + \hat{\epsilon}z$ represents the actual displacement at the centre of the considered axial interval. The special case $D + \hat{\epsilon}z = 0$ also corresponds to zero mean displacement of scatterers randomly drawn from a uniform distribution within a symmetric interval centred at z, as shown in Supplementary Note 4. This implies that, around z, the unloaded and loaded signals are expected to remain more similar than in cases with larger local mean displacement, which explains why VM and WLS can show higher accuracy in this regime.

QUERY 3.19: *- Missing script*
The script "Non-linear-dphi.m" required for Fig. 1, which is described in the README file, is missing from the provided files.
ANSWER 3.19
The script generating Fig. 1 has now been added and renamed as
`Fig1_Phase_difference_simulation.m`

QUERY 3.20: *Throughout the code, comments have not been added appropriately. In particular, the newly proposed strain estimation code (likely CSR-Tay-*.m) is difficult to understand. Please add appropriate comments to enable interpretation of routine blocks and to explain hard-coded parameters*
ANSWER 3.20
We agree with the reviewer. The file `CSR_Tay_gen.m` is the main implementation of the proposed displacement-estimation algorithm. In the revised code package, we have rewritten this file to make it easier to follow and to match the notation used in the new Supplementary Note 6 (see Lines 75–100 of the Supplementary material). We also clarify that `CSR_Tay_gen_comb.m`, which appears in the figure-reproduction scripts, is only a wrapper used to split large datasets into smaller blocks before passing them to `CSR_Tay_gen.m`; therefore, the core algorithm is contained in `CSR_Tay_gen.m`. In particular, the variable names have been made consistent with the parameters and quantities described in Supplementary Note 6, and the help text and comments have been rewritten to explain the main routine blocks and the hard-coded parameters.
These revisions now clarify all the steps of the implementation, including the reconstruction of the carrier-removed A-scans and their derivatives Note 6.1, the sparse-grid lateral-displacement estimation Note 6.2, the three-level coarse-to-fine search Note 6.3, the computation of the synthetically transformed signals in Note 6.4, and the local linear correction and residual derivatives in Notes 6.5–6.6.

Reviewer 4

This manuscript presents an innovative approach for the phase-difference base displacement estimation in optical coherence elastography. The usually approach considers a linear

relationship between phase and displacement. Here, a novel expansion for phase difference is derived, which contains higher order terms, in addition to the currently applied term. Moreover, two principal phenomena which perturb the usual assumed linear relationship between displacement and phase difference, are investigated. This work seems original and represents promising advances for displacement estimation in OCE, which overcome the limitations of existing approaches. Nevertheless, several descriptions in the manuscript lack sufficient discussion, affecting its clarity and reliability. Some comments and questions for the authors are listed below. After major revisions addressing the issues raised above, the manuscript could become suitable for publication.

QUERY 4.1: *Figure 2, gives a clear illustrative example why it is important to consider the expansion in (6) with n greater than 0, and the accuracy improves as n grows. So the claim that in OCE the choice n=1 generates a good estimation is not well justified. Wouldn't the choice n=4 improve the results?*

ANSWER 4.1

We thank the reviewer for this important comment. We agree that Fig. (2) shows that the approximation of Eq. (6) improves as the truncation order increases, and therefore that the case $n = 4$ is more accurate than lower-order approximations. Indeed, the proposed displacement-estimation method used later in the manuscript is implemented using Eq. (6) truncated at $n = 4$, as now clarified in the revised section **Novel approach for displacement estimation**, specifically in Line 455.

On the other hand, the analytical approximation of $\Delta\phi$, employs truncated versions of Eq. (6) up to order $n = 1$, however it is not intended to provide the most accurate practical approximation of $\Delta\phi$, but to show analytically that the phase difference contains intrinsic non-linear terms beyond the PHDE. For this reason, we used the minimum number of terms of Eq. (6) required to reproduce non-linear behaviours of $\Delta\phi$, while keeping the analytical derivation tractable. This also demonstrates that the non-linear oscillations of $\Delta\phi$ are not phase-unwrapping artefacts, since they can be reproduced directly by the analytical approximation shown in Eq. (10).

To reinforce the point that Eq. (10) goes beyond the PHDE, in the revised manuscript we have added a paragraph showing that the PHDE corresponds to the $n = 0$ truncation of Eq. (6), whereas Eq. (10) retains additional terms beyond $n = 0$, sufficient to reproduce the leading non-linear behaviour of $\Delta\phi$ (see Line 182):

It is worth noting that the PHDE assumption is equivalent to approximating the loaded A-scan by the $n = 0$ truncation of Eq. (6), namely $A^L(z) \approx \alpha_0(z) A_0^U(z)$. In fact, under this assumption, we get $\Delta\phi(z) = \text{Arg}\left\{A^U(z)\overline{A^L(z)}\right\} \approx \text{Arg}\left\{A^U(z)\overline{\alpha_0(z)A_0^U(z)}\right\} = \text{Arg}\left\{\overline{\alpha_0(z)}\,|A^U(z)|^2\right\} = 4\pi\xi_0(D + \hat{\epsilon}z)$, since $|A^U(z)|^2$ is real and non-negative. Therefore, by dividing both sides by $2k_0$, we obtain Eq. (1).

Additionally, the revised Fig. (2) now shows that the approximation improves as the truncation order increases, including in the presence of noise and vibrational perturbations. This reinforces that higher-order truncations provide further gains in accuracy, which is why the proposed displacement-estimation method uses $n = 4$. The reason why the analytical derivation uses only the minimum number of terms required to reproduce the leading non-linear behaviour of $\Delta\phi$ is discussed in more detail in ANSWER 4.2.

QUERY 4.2: *Why is only the case n=1 explored along the manuscript?*

ANSWER 4.2

As mentioned in ANSWER 4.1, we clarify that $n = 1$ is not the only truncation order

used in this work. In particular, the proposed displacement-estimation method is implemented using Eq. (6) truncated at $n = 4$, as now described in the revised section **Novel approach for displacement estimation** (see Line 455).

The case discussed in the analytical derivation is different. There, we use the minimum number of terms of Eq. (6) required to reproduce the leading non-linear behaviour of $\Delta\phi$ beyond the PHDE, while still allowing a tractable analytical derivation. We have revised the text to avoid suggesting that the entire manuscript only explores the case $n = 1$, or that all terms in the derivation are uniformly truncated at $n = 1$. The aim of this analytical section is to demonstrate, theoretically, the origin of the leading non-linear terms, rather than to provide the most accurate practical approximation of $\Delta\phi$. To make this point clearer, we added the following paragraph to the manuscript (see Lines 189-190):

In this section, we derive an approximation of $\Delta\phi$ based on equation (6) truncated at $n = 1$.

In this section, we derive an approximation of $\Delta\phi$ valid in bright speckles by using the definition of $\phi'(z)$ in Eq. (7). Both the numerator and denominator of the resulting ratio are expressed independently through the series representation in Eq. (6), but with different truncation orders: $n = 1$ in the numerator and $n = 0$ in the denominator. This low-order truncation was chosen because it is sufficient to reproduce the leading non-linear terms of $\Delta\phi$ beyond the PHDE while keeping the derivation analytically tractable (see Supplementary Note 2 for further details). Higher-order terms would improve the accuracy of the approximation, but they lead to substantially more complicated phase expressions and were not required to demonstrate, theoretically, the existence of these non-linear terms. Nevertheless, higher truncation orders of Eq. (6) are considered later in the manuscript.

QUERY 4.3: *The concepts of oscillation-peaks and 2π-jumps are illustrated throughout the paper, but they are not defined in a rigorous manner. A precise definition is therefore required.*
ANSWER 4.3
We thank the reviewer for this helpful comment. We agree that, in the original version of the manuscript, the concepts of oscillation-peaks and 2π-jumps were illustrated and discussed, but not defined in a sufficiently rigorous manner. To address this point, we have now added a formal definition directly in the main text (highlighted in yellow in the red-lined manuscript), rather than leaving the reader to infer these events only from the figures and surrounding discussion. In particular, we now define oscillation-peaks and 2π-jumps in terms of the local trajectories of two complex OCT A-scans in the complex plane, and we distinguish the two cases through the convex hull of these trajectories. For completeness, in the oscillation-peak case we also define the corresponding magnitude M_I, which helps clarify the definition (see Line 277):

We now give a more formal definition of these non-linear events in the general case, where $A^{(1)}(z)$ is a complex OCT A-scan and $A^{(2)}(z)$ is regarded as a perturbed version of $A^{(1)}(z)$. Such a perturbation may arise, for example, from strain, bulk motion, vibration, or noise. Let z_{ds} be a local minimum of $|A^{(1)}(z)|$, and let $\Omega_{z_{ds}}$ denote a local axial interval centred at z_{ds} over which the corresponding phase perturbation is analysed. Let $\Gamma_1 := \{A^{(1)}(z) : z \in \Omega_{z_{ds}}\}$, $\quad \Gamma_2 := \{A^{(2)}(z) : z \in \Omega_{z_{ds}}\}$ be the associated local trajectories in the complex plane. In practice, $\Omega_{z_{ds}}$ is chosen as the local interval used to isolate the single phase perturbation associated with the dark-speckle region around z_{ds}. We define the perturbation as a 2π-jump if $0 \in \mathrm{conv}(\Gamma_1 \cup \Gamma_2)$, where $\mathrm{conv}(\cdot)$ denotes the convex hull. Otherwise, it is defined as an oscillation-peak. In the latter case, its magnitude is defined as $M_I := \max_{z \in \Omega_{z_{ds}}} |\Delta\phi_{\mathrm{osc}}(z)|$, where $\Delta\phi_{\mathrm{osc}}(z)$ denotes the phase difference after subtraction of the local linear trend predicted by the PHDE.

QUERY 4.4: *Not enough detail is given about how to eveal the values of local strain and bulk displacement using equation (11).*

ANSWER 4.4

We agree with the reviewer that the original description of the proposed displacement-estimation method was not sufficiently detailed. In the revised manuscript, we have completely rewritten the section **Novel approach for displacement estimation**, which now provides a clearer description of how local strain and bulk displacement are estimated from the minimization procedure.

In addition, we have added a full step-by-step derivation and implementation description in **Supplementary Note 6** (See Lines 75-100 of the Supplementary Material). These revisions now clarify all the steps of the implementation, including the reconstruction of the carrier-removed A-scans and their derivatives Note 6.1 , the sparse-grid lateral-displacement estimation Note 6.2 , the three-level coarse-to-fine search Note 6.4 , and the local linear correction applied to each tested candidate Notes 6.4-6.6 .

References

1. R. A. Leitgeb, R. M. Werkmeister, C. Blatter, and L. Schmetterer, "Doppler Optical Coherence Tomography," Prog. Retin. Eye Res. **41**, 26–43 (2014).
2. G. Liu, A. J. Lin, B. J. Tromberg, and Z. Chen, "A comparison of Doppler optical coherence tomography methods," Biomed. Opt. Express **3**, 2669–2680 (2012).
3. B. F. Kennedy, K. M. Kennedy, and D. D. Sampson, "A review of optical coherence elastography: Fundamentals, techniques and prospects," IEEE J. on Sel. Top. Quantum Electron. **20**, p. 17 (2014).
4. M. A. Kirby, I. Pelivanov, S. Song, L. Ambrozinski, S. J. Yoon, L. Gao, D. Li, T. T. Shen, R. K. Wang, and M. O'Donnell, "Optical coherence elastography in ophthalmology," J. Biomed. Opt. **22**, p. 28 (2017).

5. B. F. Kennedy, S. H. Koh, R. A. McLaughlin, K. M. Kennedy, P. R. T. Munro, and D. D. Sampson, "Strain estimation in phase-sensitive optical coherence elastography," Biomed. Opt. Express **3**, 1865–1879 (2012).
6. A. L. Matveyev, L. A. Matveev, A. A. Sovetsky, G. V. Gelikonov, A. A. Moiseev, and V. Y. Zaitsev, "Vector method for strain estimation in phase-sensitive optical coherence elastography," Laser Phys. Lett. **15**, 065603 (2018).
7. V. Y. Zaitsev, A. L. Matveyev, L. A. Matveev, G. V. Gelikonov, V. M. Gelikonov, and A. Vitkin, "Deformation-induced speckle-pattern evolution and feasibility of correlational speckle tracking in optical coherence elastography," J. Biomed. Opt. **20**, p. 12 (2015).
8. F. Zvietcovich, P. Pongchalee, P. Meemon, J. P. Rolland, and K. J. Parker, "Reverberant 3D optical coherence elastography maps the elasticity of individual corneal layers," Nat. Commun. **10**, p. 13 (2019).
9. J. A. Izatt, M. A. Choma, and A.-H. Dhalla, "Theory of optical coherence tomography," in *Optical Coherence Tomography: Technology and Applications, Second Edition,* vol. 1 Wolfgang Drexler and James G. Fujimoto, eds. (Springer International Publishing Switzerland, 2015), chap. 2, pp. 65–94, 2nd ed.
10. J. M. Schmitt and A. Knüttel, "Model of optical coherence tomography of heterogeneous tissue," J. Opt. Soc. Am. A **14**, 1231–1242 (1997).
11. S. Azzollini, T. Monfort, O. Thouvenin, and K. Grieve, "Dynamic optical coherence tomography for cell analysis [Invited]," Biomed. Opt. Express **14**, 3362–3379 (2023).
12. H. Schulz-Hildebrandt, J. A. Gardecki, T. Miller, M. Avila-Wallace, E. Villareyna-Lopez, and G. Tearney, "Phase-sensitive dynamic micro-optical coherence tomography for high-speed intracellular motion imaging," Opt. Lett. **50**, 4734–4737 (2025).
13. J. A. Bellesini, K. Y. Foo, J. Li, R. W. Sanderson, R. Zilkens, L. Gale, M. Hardie, S. Hamza, A. Rijhumal, C. M. Saunders, and B. F. Kennedy, "Three-dimensional dynamic optical coherence tomography for breast tumor margin assessment," Biomed. Opt. Express **16** (2025).
14. B. F. Kennedy, R. A. McLaughlin, K. M. Kennedy, L. Chin, A. Curatolo, A. Tien, B. Latham, C. M. Saunders, and D. D. Sampson, "Optical coherence micro-elastography: mechanical-contrast imaging of tissue microstructure," Biomed. Opt. Express **5**, 2113–2124 (2014).
15. M. S. Hepburn, K. Y. Foo, L. Chin, P. R. T. Munro, and B. F. Kennedy, "Measuring Deformation in Optical Coherence Elastography," in *Optical Coherence Elastography,* (Springer, 2021), pp. 1–26.
16. R. K. Wang, Z. Ma, and S. J. Kirkpatrick, "Tissue Doppler optical coherence elastography for real time strain rate and strain mapping of soft tissue," Appl. Phys. Lett. **89**, p. 3 (2006).
17. L. Chin, A. Curatolo, B. F. Kennedy, B. J. Doyle, P. R. T. Munro, R. A. McLaughlin, and D. D. Sampson, "Analysis of image formation in optical coherence elastography using a multiphysics approach," Biomed. Opt. Express **5**, 2913–2930 (2014).
18. J. Li, E. Pijewska, Q. Fang, M. Szkulmowski, and B. F. Kennedy, "Analysis of strain estimation methods in phase-sensitive compression optical coherence elastography," Biomed. Opt. Express **13**, 2224–2246 (2022).
19. V. Y. Zaitsev, A. L. Matveyev, L. A. Matveev, G. V. Gelikonov, A. A. Sovetsky, and A. Vitkin, "Optimized phase gradient measurements and phase-amplitude interplay in optical coherence elastography," J. Biomed. Opt. **21**, p. 12 (2016).
20. V. Y. Zaitsev, S. Y. Ksenofontov, A. A. Sovetsky, A. L. Matveyev, L. A. Matveev, A. A. Zykov, and G. V. Gelikonov, "Real-Time Strain and Elasticity Imaging in Phase-Sensitive Optical Coherence Elastography Using a Computationally Efficient Realization of the Vector Method," Photonics **8**, p. 21 (2021).
21. B. Dong, N. Huang, Y. Bai, and S. Xie, "Deep-learning-based approach for strain estimation in phase-sensitive optical coherence elastography," Opt. Lett. **46**, 5914–5917 (2021).
22. Y. Bai, K. Zhang, R. Mo, Z. Ni, Z. He, S. Xie, and B. Dong, "Bayesian-neural-network-based strain estimation approach for optical coherence elastography," Optica **11**, 1334–1345 (2024).
23. V. Y. Zaitsev, A. L. Matveyev, L. A. Matveev, A. A. Sovetsky, M. S. Hepburn, A. Mowla, and B. F. Kennedy, "Strain and elasticity imaging in compression optical coherence elastography: The two-decade perspective and recent advances," J. Biophotonics **14**, p. 32 (2021).
24. S. Makita, F. Jaillon, I. Jahan, and Y. Yasuno, "Noise statistics of phase-resolved optical coherence tomography imaging: single-and dual-beam-scan Doppler optical coherence tomography," Opt. Express **22** (2014).
25. M. S. Hepburn, K. Y. Foo, P. Wijesinghe, P. R. T. Munro, L. Chin, and B. F. Kennedy, "Speckle-dependent accuracy in phase-sensitive optical coherence tomography," Opt. Express **29**, 16950–16966 (2021).
26. P. Dang and T. Qian, "Analytic phase derivatives, all-pass filters and signals of minimum phase," IEEE Transactions on Signal Process. **59**, 4708–4718 (2011).
27. T. R. Hillman, S. G. Adie, V. Seemann, J. J. Armstrong, S. L. Jacques, and D. D. Sampson, "Correlation of static speckle with sample properties in optical coherence tomography," Opt. Lett. **31** (2006).

Response to reviewers for manuscript: Insight into Phase-Sensitive Optical Coherence Tomography Displacement Measurement

On behalf of all authors I thank the reviewers for their carefully considered suggestions, comments and queries. We are grateful to have the opportunity to address them. We have modified the manuscript to make its purpose clearer and to address the specific queries raised by each reviewer.

In order to clearly denote changes made to the manuscript, added text is highlighted in the red-lined manuscript in yellow and deleted text is highlighted in red. The line numbers cited in this response refer to the line numbers shown in the red-lined manuscript and Supplementary Material. As highlighted text is typeset using LaTeX colour boxes, a single line number in the red-lined document may correspond to a block of text containing several visible lines of highlighted text. For example, "Line 76" corresponds to the highlighted revision blocks numbered 76 in the red-lined main manuscript, which actually contains 8 lines of text.

Reviewer 2

The authors have carefully addressed my previous comments and substantially improved the manuscript. I have only the following minor comments:

> QUERY 2.1: *The new perturbation analysis supports the possibility that dark-speckle phase instability may occur beyond compression OCE. However, it does not establish that the specific higher-order deformation model or proposed displacement-estimation algorithm applies generally to all phase-sensitive OCT modalities. Please distinguish the broader phase-instability mechanism from the compression-OCE-specific derivation and algorithm.*
>
> ANSWER 2.1
>
> We thank the reviewer for this helpful clarification. We agree that the broader dark-speckle phase-instability mechanism should be distinguished from the specific analytical derivation and displacement-estimation algorithm developed in this work. We have therefore revised the manuscript in three places.

First, we revised the final sentence of the Abstract to clarify that the general claim concerns some elements of the work performed in this work, rather than the full higher-order model or algorithm (see lines 19-20):

We believe our analysis will have a significant impact upon the way displacement is estimated from phase-sensitive OCT data beyond compression OCE, and may also be relevant to emerging phase-sensitive implementations of dynamic OCT in which phase changes are used to quantify motion.

We believe that this work will significantly impact strain retrieval in compression OCE. However, elements of this work will also influence how displacement is measured from phase-sensitive OCT data beyond compression OCE, and may be relevant to emerging phase-sensitive implementations of dynamic OCT in which phase changes are used to quantify motion.

Second, at the end of the section *"Generality of oscillation-peaks and 2π-jumps in speckled OCT signals"*, we added an explicit clarification that the phase-instability analysis concerns the broader mechanism associated with perturbed speckled complex OCT signals, whereas the series expansion in Eq. (6), the phase-difference approximation in Eq. (10), and the displacement estimation algorithm are formulated for OCE-type strain estimation, where local axial motion is represented by axial strain and bulk displacement (see line 435).

It is important to clarify that, whilst the noise and vibration perturbation analysis performed in this section concerns the broader phase-instability mechanism associated with perturbed speckled complex OCT signals, the series expansion in Eq. (6), the phase-difference approximation in Eq. (10), and the novel displacement estimation approach derived below are formulated for OCE-type strain estimation, where local axial motion is represented by axial strain and bulk displacement.

Third, we revised the closing paragraph of the Discussion to make the same distinction (see lines 854-855):

We believe that this approach will serve as a foundation for a new generation of more accurate and robust displacement estimation techniques applied widely in phase-sensitive OCT and that the concepts presented in this article enhance understanding of the phase of OCT signals.

We believe that this approach will serve as a foundation for a new generation of more accurate and robust displacement estimation techniques in OCE-type strain estimation, and that the phase-instability analysis performed in this work enhances understanding of the phase of OCT signals more broadly.

These changes are intended to preserve the broader relevance of the dark-speckle phase-instability mechanism, while avoiding the implication that the specific higher-order deformation model or displacement-estimation algorithm applies directly to arbitrary phase-sensitive OCT modalities.

QUERY 2.2: *Please define all parameters in Eq. (3) clearly at their first occurrence. In particular, clarify the physical meaning, units, and sign convention of D. The definition of W as simply the "spectral width of the light source" may be imprecise. Please define W according to the assumed Gaussian spectrum and provide its exact relationship to the source bandwidth, including whether the bandwidth is expressed as a standard deviation or FWHM and whether it is defined in wavelength, wavenumber, or spatial-frequency space.*

ANSWER 2.2

We thank the reviewer for pointing out that the definitions of D and W required clarification. The other parameters appearing in Eq. (3), including z_j, ρ_j, $\hat{\epsilon}$, and k_0,

had already been defined when first introduced in the text. We have now revised the manuscript and Supplementary Information to make the definitions of D and W more explicit.

First, we clarified the definition of the bulk displacement D at its first occurrence in the main text. We now state that, in the local displacement model $d(z) = D + \hat{\epsilon} z$, D has the same units as z (see line 73):

, with the same units as z,

We also expanded the main-text explanation of D, adding details about its sign convention (see line 76):

In this local description, D accounts for the difference between the actual local displacement at depth z and the displacement that would be obtained in a hypothetical loaded configuration where the same strain $\hat{\epsilon}$ is applied uniformly throughout the entire sample. Therefore, $D > 0$ means that the actual loaded position is shifted towards larger z compared with this hypothetical uniformly strained loaded configuration, whereas $D < 0$ means that it is shifted towards smaller z

Second, since this revision provides a more precise definition of the spectral profile, we also specify that the Gaussian profile considered here is the Gaussian power spectral density, rather than a field-amplitude spectrum (see line 150):

Gaussian spectrum Gaussian power spectral density

and we replaced the previous description of W as the "spectral width of the light source" with a more precise definition at its first occurrence. Here, W is not specified through a standard deviation or FWHM bandwidth, but is defined from the full spatial-frequency interval B and from the prescribed edge level τ. Specifically, τ is the relative value of the Gaussian power spectral density at the spectral edges compared with its peak value (see lines 155-156):

, and W is the spectral width of the light source

The choice of the parameter W controls the width of the Gaussian terms in Eq. (2), and W has inverse units of z, typically m^{-1}. Specifically, let ξ denote the spatial-frequency variable Fourier-conjugate to z, and let $\xi_0 = \frac{k_0}{2\pi}$ be the central spatial frequency. In Eq. (2) and in the rest of this article we assume a Gaussian power spectral density $S_G(\xi) := \frac{1}{\sqrt{\pi} W} e^{-\left(\frac{2\pi(\xi-\xi_0)}{W}\right)^2}$. In practice, starting from a finite full spectral interval B in spatial-frequency space, W is chosen by prescribing the relative value τ of $S_G(\xi)$ at the interval edges, yielding $W = \frac{\pi B}{\sqrt{\log(1/\tau)}}$. In the simulations, we use $\tau \leq 10^{-2}$, so that the Gaussian power spectral density is negligible at the edges of the spectral interval. We also verified that the method works for larger values of τ, for which $S_G(\xi)$ has a non-negligible value at the interval edges. For general experimental data, this Gaussian-spectrum assumption can be satisfied in practice by reshaping the measured power spectral density to a Gaussian profile before applying the algorithm; further details are provided in Supplementary Notes 7.1 and 7.2.

Third, we added a new Supplementary Note 7, titled:

Gaussian spectral profile, width definition, and experimental spectral reshaping, where we provide the full details of the definition of W and the Gaussian spectrum assumption used in the article. In particular, in the Supplementary note 7.1, we relate the wavelength interval $[\lambda_{\min}, \lambda_{\max}]$ to the spatial-frequency interval through $\xi_{\min} = 1/\lambda_{\max}$, $\xi_{\max} = 1/\lambda_{\min}$, and $B = \xi_{\max} - \xi_{\min}$.

In Supplementary Note 7.2, we provide the corresponding details of this spectral reshaping procedure, including the use of the same reshaping for the unloaded and loaded spectra. We thought it was useful to also report this preprocessing step, which makes the experimental spectra more consistent with the theoretical framework used

to derive the proposed displacement-estimation algorithm.

Fourth, we revised the Methods section to state explicitly that, following the above definition of W, the simulations used $\lambda_{\text{min}} = 1170.5$ nm, $\lambda_{\text{max}} = 1407.8$ nm, $\tau = 10^{-3}$, and $W \approx 1.7 \times 10^5$ m^{-1} (see lines 863-864):

In particular, we assumed a central wavelength of 1278 nm and a spectral width of 237 nm. This corresponds to a central wavenumber of $k_0 \approx 4.9 \times 10^6 m^{-1}$. The parameter W was chosen so that the Gaussian spectrum centered at k_0 was approximately three orders of magnitude smaller at the spectral edges, yielding $W \approx 1.7 \times 10^5\, m^{-1}$.

In particular, we assumed a wavelength interval from $\lambda_{\text{min}} = 1170.5$ nm to $\lambda_{\text{max}} = 1407.8$ nm, corresponding to a wavelength span of approximately 237 nm. This gives a central spatial frequency $\xi_0 = (\lambda_{\text{min}}^{-1} + \lambda_{\text{max}}^{-1})/2$, or equivalently a central wavenumber $k_0 = 2\pi\xi_0 \approx 4.9 \times 10^6\, m^{-1}$. The corresponding full spectral interval in spatial-frequency space is $B = \lambda_{\text{min}}^{-1} - \lambda_{\text{max}}^{-1}$. The parameter W was then chosen using $W = \pi B/\sqrt{\log(1/\tau)}$, where τ is the prescribed relative Gaussian level at the interval edges. For these simulations, $\tau = 10^{-3}$ was used, so that the Gaussian power spectral density was approximately three orders of magnitude lower than its peak value at the spectral edges, yielding $W \approx 1.7 \times 10^5\, m^{-1}$ (see the Supplementary Note 7.1 for further details).

After providing these additional details on W and on the Gaussian power spectral density assumption, we also thought it useful to state explicitly how this assumption enters the displacement-estimation formulation. In the main manuscript, we now state that $I(\xi)$ is assumed to have a Gaussian-shaped power spectral density and clarify that, for experimental data, the measured power spectral density can be reshaped to a Gaussian profile before applying the algorithm (see lines 665-667):

, from which the derivatives of $A_{\text{scan}}(z)$ (defined in Eq. (3)) are computed as:

.

In this formulation, $I(\xi)$ is assumed to have a Gaussian-shaped power spectral density. This assumption is not overly restrictive, as the power spectral density can be reshaped to a Gaussian profile (see Supplementary Note 7.2 for further details), and we have observed heuristically that the proposed displacement-estimation model is robust to this reshaping step. The derivatives of $A_{\text{scan}}(z)$ (defined in Eq. (3)) are then computed from $I(\xi)$ as:

These additions define all parameters in Eq. (3) at their first occurrence and clarify the physical meaning, units, and sign convention of D, as well as the precise meaning of W and its relationship to the assumed Gaussian power spectral density.

QUERY 2.3: *The additional noise and vibrational-motion simulations are helpful. However, the relative error reaches approximately 17.4% under the combined r=5% and D_v=10 nm condition, which may not warrant the description "accurate." Moreover, this analysis evaluates the truncated signal expansion rather than the complete strain-retrieval algorithm. Please moderate the robustness claim and clarify this distinction.*

ANSWER 2.3

We agree with the reviewer that the 17.4% error in the strongest perturbation case does not, by itself, prove robustness of the complete strain-retrieval algorithm. We have therefore moderated the robustness/accuracy wording in the manuscript, avoiding any implication that this specific A-scan-level test directly demonstrates the final robustness of the complete method. In particular, we have changed the description of Fig. (2): (see lines 250-251)

We emphasize that equation (6) is not only theoretically significant, but it can also be effectively utilised to make robust displacement predictions based on experimental data, as will be discussed in the **Methods** section.

We emphasize that equation (6) is theoretically significant and validates the essential building block required by the displacement-estimation method applied to experimental data, as discussed in the **Methods** section.

We also moderated the wording at the end of the Results section (see lines 786-787):

We provide this demonstration as proof of principle that it is possible to retrieve strain robustly and accurately in a manner immune to the limitations of conventional approaches.

These proof-of-principle results show that the proposed approach can provide more accurate and stable strain estimates than conventional phase-difference methods, by avoiding their key modelling limitations.

and (see lines 803-804):

These results show that the implementation used in this work is considerably slower than the WLS and VM methods, but it demonstrates that substantially more robust and accurate strain estimation can be achieved by modelling the OCT signals beyond the conventional phase-difference assumption.

These results show that the implementation used in this work is considerably slower than the WLS and VM methods, but they also demonstrate that modelling the OCT signals beyond the conventional phase-difference assumption enables more accurate and robust strain estimation.

We would also like to add some additional context, beyond the manuscript revisions, on how the 17.4% A-scan-level error should be interpreted in relation to strain retrieval. In particular, approximating the loaded complex OCT signal and estimating strain from that signal model are related, but not equivalent, tasks. Our experience is that strain can be retrieved more accurately, in the presence of noise, than the error of approximating the loaded complex OCT signal would suggest. This is because all strain retrieval methods, including WLS, VM and our method, apply either averaging or a fitting procedure that mitigates the impact of noise in the complex OCT signal on the retrieved strain.

For example, the $n = 0$ truncation of Eq. (6) corresponds to the conventional phase-difference assumption used by WLS and VM (see lines 243-249). In panel (f) of Fig. (2), this $n = 0$ approximation gives a relative error of approximately 58% in the complex loaded A-scan even in the noise-free case (see plot (b)). Nevertheless, when the same phase-difference assumption is used within the conventional WLS and VM strain-retrieval methods, the relative errors of the retrieved strain maps remain mainly below approximately 10% (see Fig. (8)).

A similar comparison can be made for our proposed method in the noise-free case. The fourth-order truncated signal expansion gives a relative loaded A-scan error of approximately 1.7% (see line 237), corresponding to the red cross in panel j) and to plot d) of Fig. (2). However, in the separate strain-estimation simulations shown later in the top plots of Fig. (9), which use the same $n = 4$ truncation but different OCT signals generated under the same underlying assumptions, the strain errors are below 0.1%, which is substantially smaller than the corresponding complex A-scan approximation error for all tested cases (see plots a)-d) of Fig. (9)). Although this is not a direct comparison on the same A-scans, it illustrates why an A-scan-level approximation error should not be interpreted directly as the expected strain-retrieval error.

This interpretation is also consistent with our previous OCT simulation work [28],

where we compared the approximation error of a different OCT simulator with the error of the corresponding OCE strain map computed using WLS (see Fig. 6 of [28]). In that case, the OCE error was at least one order of magnitude smaller than the corresponding loaded-OCT signal approximation error. Although that study used a different OCT simulation model, it supports the general point that complex OCT-signal errors and strain-retrieval errors should not be treated as equivalent quantities, where the former can be larger. We believe this is reasonable because strain retrieval is obtained through a fitting procedure over an axial range, so local discrepancies in the complex A-scan approximation may be partly averaged during the estimation.

We decided not to add text to the manuscript explaining this distinction because we have not performed sufficient tests required to prove this explanation. Rather, the evidence has been acquired incidentally, and is not complete. Further, we felt that there would be a risk of confusing the reader by adding another nuanced point which we believe is not central to the claims of the paper.

QUERY 2.4: *For reproducibility, please provide the hardware specifications and dataset dimensions associated with the reported processing times.*
ANSWER 2.4

We thank the reviewer for pointing this out. We have revised the runtime description to include both the hardware specifications and the dataset dimensions used for the reported processing times.

First, we added the hardware specifications and clarified that the reported implementations were CPU-based, despite the workstation having an available GPU (see lines 811-812):

The reported runtimes correspond to the current MATLAB implementations, which were written in vectorized form.

The reported runtimes correspond to the current vectorized MATLAB implementations, run on a workstation equipped with two AMD EPYC™ 7453 28-core CPUs.

We also added the dimensions of the dataset used for the timing comparison (see line 802):

The raw 3D OCT spectral datasets processed for this timing comparison contained 2048 wavelength samples and 2000×1000 lateral positions in the x and y directions, respectively. Thus, each dataset contained 2000×1000 A-scans, each sampled over 2048 spectral points. For visualization only, the images shown in the figures were cropped around the inclusion, displaying a region of 800×624 lateral pixels in the x and y directions, respectively.

Reviewer 3

(Remarks to the Author):
The authors have thoroughly addressed the reviewers' concerns, and the manuscript has been substantially improved as a result. I commend the authors on their thorough revisions and have no further comments.

(Remarks on code availability):
The code can reproduce the results of the paper. This is valuable to the community, as it enables reanalysis of the phase-related issues identified in the manuscript and the reuse of the proposed algorithm. This will contribute to the development of algorithms that address issues in other types of phase-sensitive OCT. Some revisions are still required.

> QUERY 3.1: *Unnecessary lines are included in "Figs7-8-WLS-VM-analysis.m" script, thus the script cannot run without modifications.:*
> *- undeclared, and perhaps not necessary, function "get-info()"*
> *- Multiple definitions of the "saving-file" variable*
> ANSWER 3.1:
> We thank the reviewer for identifying this issue. We have corrected the script and verified that it now runs correctly. The revised version has been included in the updated Supplementary Dataset.
> We would also like to thank the reviewer once again for the detailed and careful assessment provided during the first round of review, which helped us to substantially strengthen the manuscript.

Reviewer 4

(Remarks to the Author):
This manuscript presents an innovative approach for the phase-difference base displacement estimation in optical coherence elastography. The usually approach considers a linear relationship between phase and displacement. Here, a novel expansion for phase difference is derived, which contains higher order terms, in addition to the currently applied term. Moreover, two principal phenomena which perturb the usual assumed linear relationship between displacement and phase difference, are investigated. This work seems original and represents promising advances for displacement estimation in OCE, which overcome the limitations of existing approaches. Several issues identified in the previous version of the manuscript have now been satisfactorily addressed. I have reviewed the authors' responses to the comments and appreciate their efforts in resolving all the my previous concerns.The article is of sufficient interest and quality and is recommended for publication in its current form.

(Remarks on code availability):
The results are reproducible.

References

1. R. A. Leitgeb, R. M. Werkmeister, C. Blatter, and L. Schmetterer, “Doppler Optical Coherence Tomography,” Prog. Retin. Eye Res. **41**, 26–43 (2014).
2. G. Liu, A. J. Lin, B. J. Tromberg, and Z. Chen, “A comparison of Doppler optical coherence tomography methods,” Biomed. Opt. Express **3**, 2669–2680 (2012).
3. B. F. Kennedy, K. M. Kennedy, and D. D. Sampson, “A review of optical coherence elastography: Fundamentals, techniques and prospects,” IEEE J. on Sel. Top. Quantum Electron. **20**, p. 17 (2014).
4. M. A. Kirby, I. Pelivanov, S. Song, L. Ambrozinski, S. J. Yoon, L. Gao, D. Li, T. T. Shen, R. K. Wang, and M. O’Donnell, “Optical coherence elastography in ophthalmology,” J. Biomed. Opt. **22**, p. 28 (2017).
5. B. F. Kennedy, S. H. Koh, R. A. McLaughlin, K. M. Kennedy, P. R. T. Munro, and D. D. Sampson, “Strain estimation in phase-sensitive optical coherence elastography,” Biomed. Opt. Express **3**, 1865–1879 (2012).
6. A. L. Matveyev, L. A. Matveev, A. A. Sovetsky, G. V. Gelikonov, A. A. Moiseev, and V. Y. Zaitsev, “Vector method for strain estimation in phase-sensitive optical coherence elastography,” Laser Phys. Lett. **15**, 065603 (2018).
7. V. Y. Zaitsev, A. L. Matveyev, L. A. Matveev, G. V. Gelikonov, V. M. Gelikonov, and A. Vitkin, “Deformation-induced speckle-pattern evolution and feasibility of correlational speckle tracking in optical coherence elastography,” J. Biomed. Opt. **20**, p. 12 (2015).
8. F. Zvietcovich, P. Pongchalee, P. Meemon, J. P. Rolland, and K. J. Parker, “Reverberant 3D optical coherence elastography maps the elasticity of individual corneal layers,” Nat. Commun. **10**, p. 13 (2019).
9. J. A. Izatt, M. A. Choma, and A.-H. Dhalla, “Theory of optical coherence tomography,” in *Optical Coherence Tomography: Technology and Applications, Second Edition,* vol. 1 Wolfgang Drexler and James G. Fujimoto, eds. (Springer International Publishing Switzerland, 2015), chap. 2, pp. 65–94, 2nd ed.
10. J. M. Schmitt and A. Knüttel, “Model of optical coherence tomography of heterogeneous tissue,” J. Opt. Soc. Am. A **14**, 1231–1242 (1997).
11. S. Azzollini, T. Monfort, O. Thouvenin, and K. Grieve, “Dynamic optical coherence tomography for cell analysis [Invited],” Biomed. Opt. Express **14**, 3362–3379 (2023).
12. H. Schulz-Hildebrandt, J. A. Gardecki, T. Miller, M. Avila-Wallace, E. Villareyna-Lopez, and G. Tearney, “Phase-sensitive dynamic micro-optical coherence tomography for high-speed intracellular motion imaging,” Opt. Lett. **50**, 4734–4737 (2025).
13. J. A. Bellesini, K. Y. Foo, J. Li, R. W. Sanderson, R. Zilkens, L. Gale, M. Hardie, S. Hamza, A. Rijhumal, C. M. Saunders, and B. F. Kennedy, “Three-dimensional dynamic optical coherence tomography for breast tumor margin assessment,” Biomed. Opt. Express **16** (2025).
14. B. F. Kennedy, R. A. McLaughlin, K. M. Kennedy, L. Chin, A. Curatolo, A. Tien, B. Latham, C. M. Saunders, and D. D. Sampson, “Optical coherence micro-elastography: mechanical-contrast imaging of tissue microstructure,” Biomed. Opt. Express **5**, 2113–2124 (2014).
15. M. S. Hepburn, K. Y. Foo, L. Chin, P. R. T. Munro, and B. F. Kennedy, “Measuring Deformation in Optical Coherence Elastography,” in *Optical Coherence Elastography,* (Springer, 2021), pp. 1–26.
16. R. K. Wang, Z. Ma, and S. J. Kirkpatrick, “Tissue Doppler optical coherence elastography for real time strain rate and strain mapping of soft tissue,” Appl. Phys. Lett. **89**, p. 3 (2006).
17. L. Chin, A. Curatolo, B. F. Kennedy, B. J. Doyle, P. R. T. Munro, R. A. McLaughlin, and D. D. Sampson, “Analysis of image formation in optical coherence elastography using a multiphysics approach,” Biomed. Opt. Express **5**, 2913–2930 (2014).
18. J. Li, E. Pijewska, Q. Fang, M. Szkulmowski, and B. F. Kennedy, “Analysis of strain estimation methods in phase-sensitive compression optical coherence elastography,” Biomed. Opt. Express **13**, 2224–2246 (2022).
19. V. Y. Zaitsev, A. L. Matveyev, L. A. Matveev, G. V. Gelikonov, A. A. Sovetsky, and A. Vitkin, “Optimized phase gradient measurements and phase-amplitude interplay in optical coherence elastography,” J. Biomed. Opt. **21**, p. 12 (2016).
20. V. Y. Zaitsev, S. Y. Ksenofontov, A. A. Sovetsky, A. L. Matveyev, L. A. Matveev, A. A. Zykov, and G. V. Gelikonov, “Real-Time Strain and Elasticity Imaging in Phase-Sensitive Optical Coherence Elastography Using a Computationally Efficient Realization of the Vector Method,” Photonics **8**, p. 21 (2021).
21. B. Dong, N. Huang, Y. Bai, and S. Xie, “Deep-learning-based approach for strain estimation in phase-sensitive optical coherence elastography,” Opt. Lett. **46**, 5914–5917 (2021).
22. Y. Bai, K. Zhang, R. Mo, Z. Ni, Z. He, S. Xie, and B. Dong, “Bayesian-neural-network-based strain estimation approach for optical coherence elastography,” Optica **11**, 1334–1345 (2024).
23. V. Y. Zaitsev, A. L. Matveyev, L. A. Matveev, A. A. Sovetsky, M. S. Hepburn, A. Mowla, and B. F. Kennedy, “Strain and elasticity imaging in compression optical coherence elastography: The two-decade perspective and recent advances,” J. Biophotonics **14**, p. 32 (2021).
24. S. Makita, F. Jaillon, I. Jahan, and Y. Yasuno, “Noise statistics of phase-resolved optical coherence tomography imaging: single-and dual-beam-scan Doppler optical coherence tomography,” Opt. Express **22** (2014).
25. M. S. Hepburn, K. Y. Foo, P. Wijesinghe, P. R. T. Munro, L. Chin, and B. F. Kennedy, “Speckle-dependent accuracy in phase-sensitive optical coherence tomography,” Opt. Express **29**, 16950–16966 (2021).
26. P. Dang and T. Qian, “Analytic phase derivatives, all-pass filters and signals of minimum phase,” IEEE Transactions on Signal Process. **59**, 4708–4718 (2011).
27. T. R. Hillman, S. G. Adie, V. Seemann, J. J. Armstrong, S. L. Jacques, and D. D. Sampson, “Correlation of static speckle with sample properties in optical coherence tomography,” Opt. Lett. **31** (2006).
28. A. Mazzolani, C. Macdonald, and P. R. T. Munro, “Fast and customizable image formation model for optical coherence tomography,” Biomed. Opt. Express **15**, 6783–6798 (2024).
